# Distinct genomic and immunologic tumor evolution in germline *TP53-*driven breast cancers

**DOI:** 10.1038/s41467-026-73163-4

**Published:** 2026-05-21

**Authors:** Nabamita Boruah, David Hoyos, Renyta Moses, Ryan Hausler, Heena Desai, Anh N. Le, Madeline Good, Gregory Kelly, Ashvathi Raghavakaimal, Maliha Tayeb, Mohana Narasimhamurthy, Abigail Doucette, Peter Gabriel, Michael J. Feldman, Jinae Park, Miguel Lopez de Rodas, Kurt A. Schalper, Shari B. Goldfarb, Anupma Nayak, Arnold J. Levine, Benjamin D. Greenbaum, Kara N. Maxwell

**Affiliations:** 1 Division of Hematology-Oncology, Department of Medicine, Perelman School of Medicine, University of Pennsylvania, Philadelphia, PA USA; 2 Computational Oncology, Department of Epidemiology & Biostatistics, Memorial Sloan Kettering Cancer Center, New York, NY USA; 3 Department of Pathology and Laboratory Medicine, Perelman School of Medicine, University of Pennsylvania, Philadelphia, PA USA; 4 Abramson Cancer Center, Perelman School of Medicine, University of Pennsylvania, Philadelphia, PA USA; 5 Department of Radiation Oncology, Perelman School of Medicine, University of Pennsylvania, Philadelphia, PA USA; 6 Departments of Medicine and Epidemiology and Biostatistics, Memorial Sloan Kettering Cancer Center, New York, NY USA; 7 Department of Pathology, Yale School of Medicine, New Haven, CT USA; 8 Department of Medicine, Weill Cornell Medical Center, New York, NY USA; 9 Institute for Advanced Study, Princeton, NJ USA; 10 Department of Physiology, Biophysics & Systems Biology, Weill Cornell Medical Center, New York, NY USA; 11 The Olayan Center for Cancer Vaccines, Memorial Sloan Kettering Cancer Center, New York, NY USA; 12 Corporal Michael Crescenz Veterans Affairs Medical Center, Philadelphia, PA USA

**Keywords:** Cancer genetics, Breast cancer, Cancer genetics, Cancer genomics, Tumour immunology

## Abstract

Pathogenic germline *TP53* alterations cause Li-Fraumeni Syndrome (LFS), and breast cancer (BC) is the most common cancer in LFS females. Here, we perform a multimodal analysis, comparing LFS-BC with sporadic premenopausal breast cancer which shows that nearly all LFS-BC undergo biallelic loss of *TP53*, with no recurrent oncogenic variants except *ERBB2* (HER2) amplification. Compared to sporadic BC, in situ and invasive LFS-BC exhibit a high burden of short amplified aneuploid segments. Pro-apoptotic p53 target genes *BAX* and *TP53I3* fail to be up-regulated in LFS-BC unlike in sporadic BC compared to normal breast tissue. LFS-BC has lower CD8 + T-cell infiltration compared to sporadic BC yet higher levels of proliferating cytotoxic T-cells. Within LFS-BC, progression from in situ to invasive BC is marked by an increase in chromosomal instability with a decrease in proliferating cytotoxic T-cells. Our study uncovers critical events in mutant p53-driven tumorigenesis in breast tissue.

## Abstract (web summary)

Breast cancer (BC) is the most common malignancy in female patients with Li-Fraumeni Syndrome (LFS). Here, the authors perform a multimodal analysis of LFS breast cancer (LFS-BC) compared to sporadic premenopausal BC, finding important differences in genomic alterations and instability, gene regulation, and the immune microenvironment.

## Introduction

Acquired somatic *TP53* alterations are the most common genomic alteration in cancer^1^. Oncogenic alterations in *TP53* lead to the loss of p53 tumor suppressive function, resulting in increased cellular proliferation, genomic instability, and therapy resistance^2,3^. Pathogenic germline variants (PGVs) in *TP53* occur in approximately 1:3000–1:10,000 people and lead to the Li Fraumeni syndrome (LFS) spectrum in which individuals have up to a 90% risk of developing cancer over their lifetimes^4–6^. The most common cancer in females with LFS is breast cancer (BC), which occurs at a median age of onset of 30–35, nearly 30 years younger than sporadic BC^7–11^.

Over 70% of sporadic BC are positive for the estrogen receptor (ER+) and negative for receptor tyrosine-protein kinase erbB-2 (HER2)^12,13^. Typically, LFS-BC are ER+; over half are HER2+ and triple negative BC (TNBC) is rarely found in LFS females^8,14–16^. In pre-invasive breast cancers in LFS patients, *TP53* mutations are associated with high-grade histology^14^. The hormone receptor distribution is peculiar, as somatically acquired *TP53* alterations are present in 80% of sporadic TNBCs versus only 10–15% of sporadic ER+ BC (with or without HER2+)^1^. This distribution of hormone receptor subtypes, along with the younger age of onset of BC in LFS patients compared to sporadic BC, suggests unique mechanisms of breast tumor formation driven by *TP53* mutations in the germline. Indeed, a recent study of pediatric LFS tumors showed that *TP53* PGVs are associated with earlier biallelic loss of *TP53* compared to sporadic *TP53* mutant tumors^17^.

Beyond genomic changes, breast tumor development depends on tumor microenvironment (TME) alterations that facilitate tumor growth. p53 dysfunction may reprogram the TME, leading to an altered immunological response, which aggravates tumor progression^18,19^. Prior work has suggested the importance of age, estrogen levels and p53 mutation status on immune infiltration in breast cancer; however, these studies only addressed sporadic p53 mutant tumors^20,21^.

In this study, we systematically compared LFS-BC to sporadic premenopausal breast cancers. LFS-BC undergo early biallelic *TP53* loss, harbor unique short amplified aneuploid segments, fail to up-regulate key p53 target genes, and display an altered immune microenvironment. Progression from in situ to invasive disease is accompanied by increased chromosomal instability and reduced proliferating cytotoxic T cells. The unique characteristics of LFS-BC suggest distinct mechanisms of tumorigenesis compared to sporadic breast cancers, providing a compelling opportunity to study mutant p53-driven breast tumor initiation and development in humans. These findings define critical genomic and immune features of germline *TP53*-driven tumorigenesis and have important implications for early detection and potential cancer interception strategies in LFS patients.

## Results

### Clinical characteristics of LFS and early-onset non-LFS breast cancer

The Penn Medicine LFS-BC cohort included 93 females with 130 BC cases (Table 1); 76% of patients were of self-identified white race/ethnicity, 9% were self-identified black, and 6% were Asian. The pre-menopausal nonLFS-BC cohort included 198 females with 209 BC cases; of these, 69% of patients self-identified as white race/ethnicity, 23% were self-identified black, and 4% were Asian. The median (IQR) age of first BC diagnosis was significantly younger in the LFS-BC compared to the non-LFS-BC cohort [36(29–45) vs. 43(39–47), *p* < 0.0001]. Fifteen percent of LFS-BC patients were from families who met classic LFS criteria, and 64% met Chompret criteria. LFS-BC patients were significantly more likely than non-LFS-BC patients to have a personal history of breast and another cancer, including another BC (64% vs. 18%, *p* < 0.0001). The majority of 74 LFS (92%) and 203 non-LFS (86%) invasive BC were ER+; however, LFS-BC were more likely to be HER2+ (51% vs. 20%, *p* < 0.0001). Invasive LFS-BC were more frequently high grade (67% vs. 47%, *p* = 0.01). The AJCC stage distribution of LFS-BC and non-LFS-BC was similar.

**Table 1:** Clinical and pathological characteristics of the cohort

	LFS-BC		Early onset non-LFS-BC		
	*N*	%	*n*	%	*p*-value
*N*/number of BC	*n* = 93/130		*n* = 198/209		
Age at 1st BC diagnosis	(*n* = 93)		(*n* = 198)		5.4e10^−^^10^
Age median (IQR)	36 (29–45)		43 (39–47)		
Self-reported race	(*n* = 93)		(*n* = 198)		0.0082
non-Hispanic white	71	76.3%	136	68.7%	
African American	8	8.6%	46	23.2%	
Asian	6	6.5%	7	3.5%	
Mixed race	2	2.2%	0	0.0%	
Other	6	6.5%	9	4.5%	
LFS classification	(*n* = 86)		n/a		
Classical	13	15.1%			
Chompret	55	64.0%			
LFL (Birch or Eeles)	48	55.8%			
Vital status	(*n* = 93)		(*n* = 198)		0.051
Deceased	19	20.4%	23	11.6%	
Personal cancer history	(*n* = 93)		(*n* = 198)		4.5e10^−14^
BC + non-breast	43	46.2%	26	13.1%	
Multiple BC	17	18.3%	10	5.1%	
Single BC	33	35.5%	162	81.8%	
Disease progression	(*n* = 91)		(*n* = 197)		0.5006
Local recurrence	4	4.4%	13	6.6%	
Distant metastasis	13	14.3%	37	18.8%	
Breast cancer characteristics					
Invasive histology	(*n* = 93)		(*n* = 209)		0.0015
IDC	75	80.6%	151	72.2%	
ILC and/or mixed	10	10.8%	53	25.4%	
Other/NOS	8	8.6%	5	2.4%	
Invasive HR status	(*n* = 74)		(*n* = 203)		1.5e10^−6^
ER+/HER2-	30	40.5%	136	67.0%	
HER2+	38	51.4%	40	19.7%	
TNBC	6	8.1%	27	13.3%	
Grade	(*n* = 60)		(*n* = 171)		0.0128
Low (1)	2	3.3%	23	13.5%	
Intermediate (2)	18	30.0%	68	39.8%	
High (3)	40	66.7%	80	46.8%	
Invasive stage	(*n* = 70)		(*n* = 125)		0.6538
I	31	44.3%	45	36.0%	
II	25	35.7%	53	42.4%	
III	11	15.7%	23	18.4%	
IV	3	4.3%	4	3.2%	

*BC* breast cancer, *ER* estrogen receptor, *IDC* invasive ductal carcinoma, *ILC* invasive lobular carcinoma, *LFL* Li-Fraumeni-like criteria including Birch and Eeles criteria, *LFS* Li Fraumeni syndrome, *NOS* not otherwise specified, *TNBC* triple negative breast cancer. Continuous variables were compared by an unpaired two-tailed Student’s *t* test. Rates in 2 subgroups between LFS-BC and non-LFS-BC were compared with a Fisher’s exact test. Rates of >2 sub-groups between LFS-BC and non-LFS-BC were compared with a chi-squared test.

### Multi-omic analysis of LFS breast cancer

Breast tumors, including both invasive ductal carcinoma (IDC) and ductal carcinoma in situ (DCIS), surrounding normal breast tissue (SNBT), contralateral normal breast tissue (CNBT), and blood specimens were obtained for 28 breast cancers from 22 patients with LFS and 29 breast cancers from 28 early-onset non-LFS patients and subjected to multi-omic studies (Supplementary Fig. 1 and Supplementary Data 1). Ten patients with 14 tissues had germline DNA binding domain mutations, including known dominant negative loss of function mutations (G245S, R248Q, and R110L) and putative hypomorphic mutations (T125M, R181H, P151S, P151T, and C141Y), eight patients with nine tissues had germline loss of function mutations (large deletions, c.993+1G > A, p.C123X, p.T125T, and p.R196X), and four patients with five tissues had germline tetramerization domain mutations (putative hypomorphic mutations, G334R, R337H and loss of function mutation, R337C) (Supplementary Fig. 1 and Supplementary Data 1).

### *TP53* status of LFS breast cancer

**Fig. 1:**
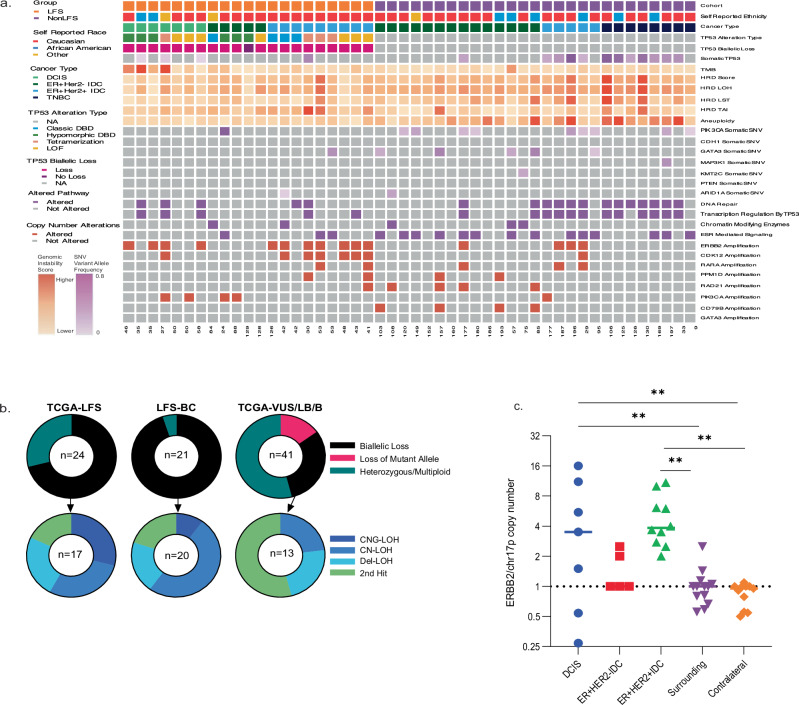
Genomic features of LFS-BC compared to early onset non-LFS-BC. **a** Heat-map showing overall clinical and genomic features of LFS-BC (*n* = 21) and nonLFS-BC (*n* = 27), including self-reported ethnicity, *TP53* germline and somatic variant status and variant alteration type, breast cancer histology, and hormone receptor class, *TP53* biallelic loss status, tumor mutational burden, overall homologous recombination deficiency (HRD) score, genomic loss of heterozygosity (LOH) score, large state transition (LST) score, non-telomeric allelic imbalance (TAI) score, aneuploidy score, oncogenic mutations, gene-level amplifications/deletions, and mutated gene oncogenic pathways. **b** State of the germline *TP53* locus in LFS-BC (*n* = 21), tumors from TCGA with germline *TP53* PGVs (*n* = 24) and tumors from TCGA with germline *TP53* LB/B-VUS (*n* = 41). In tumors with biallelic loss, the mechanism of biallelic loss is shown, including copy-neutral loss of heterozygosity (CN-LOH), copy-neutral gain loss of heterozygosity (CNG-LOH), deletion loss of heterozygosity (Del-LOH), and a second somatic mutation in *TP53* (2nd hit). **c** Amplification of *ERBB2* (HER2) in LFS-associated breast cancer and normal breast tissues. *ERBB2* copy number states were normalized to the copy number state of chromosome 17p in LFS-DCIS (*n* = 7), LFS normal surrounding tissue (*n* = 14), LFS normal contralateral tissue (*n* = 11), LFS-ER⁺HER2- IDC (*n* = 6) and LFS-ER⁺HER2⁺ IDC (*n* = 12) and shown as a dot plot with a line at the median. *P*-values were calculated using one-way ANOVA followed by Bonferroni’s multiple comparison corrections test, **p < 0.01. CN copy neutral, DBD DNA binding domain, DCIS ductal carcinoma in situ, Del deletion, ER estrogen receptor, HRD homologous recombination deficiency, HER2 receptor tyrosine-protein kinase erbB-2, IDC invasive ductal carcinoma, LB/B likely benign/benign variants, LFS Li Fraumeni syndrome, LOF loss of function, LOH loss of heterozygosity, LP/P likely pathogenic/pathogenic variants, LST large state transitions, SNV single nucleotide variant, TAI telomeric allelic imbalances, TMB tumor mutational burden, VUS variants of uncertain significance. Source data are provided as a Source data file.

Tumors with sufficient material for p53 immunohistochemistry (IHC) and targeted next-generation sequencing (NGS) analysis were available for 21 LFS-BC, 16 surrounding normal and 11 contralateral normal specimens (Fig. 1, Supplementary Fig. 1, and Supplementary Data 1). Sixteen (76%, 16/21) of LFS-BC had locus-specific loss of heterozygosity (LOH) and four (19%, 4/21) had an acquired *TP53* somatic mutation (Fig.1b, Table 2, and Supplementary Data 2). Our study identified acquired *TP53* mutations in three DCIS samples (43%, 3/7), specifically p.L265Rfs*80, p.G154V, and p.Y163S, while the one IDC sample exhibited a p.N247I *TP53* mutation (7%, 1/14). No surrounding and contralateral tissue had acquired *TP53* mutations. High p53 IHC nuclear expression (>50%) was seen in the majority of LFS-BC from patients with missense mutations, whereas no protein expression (<1%, null pattern) was seen in the majority of tumors from carriers of large exonic deletion, truncating, and splicing mutations in *TP53*. Combining p53 IHC and sequencing results, we found evidence of biallelic loss of *TP53* (fully mutant p53) in 20/21 (95%) LFS-BC, 2/15 SBNT (13%) and no CNBT (Fig. 1a and Table 2). Four cases (19%, two with T125M and two with R110L) were heterozygous diploid by genomic analysis but had greater than 50% of cells with 3+ p53 IHC staining and were called as biallelic loss, presumably due to LOH not detected by genomic means in tumors with admixture of normal cells.

**Table 2:** Summary of key genomic differences between LFS-BC and non-LFS-BC

	LFS-BC DCIS (*n* = 7)	non-LFS TNBC (*n* = 8)	LFS ER + Her2-IDC (*n* = 5)	non-LFS ER + Her2-IDC (*n* = 14)	*p*-value	LFS ER + Her2+ IDC (*n* = 9)	non-LFS ER + Her2+ IDC (*n* = 5)	*p*-value
Oncogenic alterations	*n*(%)	*n*(%)	*n*(%)	*n*(%)		*n*(%)	*n*(%)	
LOH germline mutation	7(100%)	n/a	4(80%)	n/a	n/a	9(100%)	n/a	n/a
Acquired *TP53* mutation	3(43%)	7(88%)	0(0%)	2(14%)		1(11%)	3(60%)	
Genes recurrent SNVs^a^	0(0%)	2(25%)	0(0%)	2(14%)		0(0%)	1(20%)	
Genes recurrent CNVs^a^	2(29%)	0(0%)	0(0%)	3(21%)		4(44%)	1(20%)	
Genomic instability								
MS status (%)	6.4 + 2.7	22.3 + 6.6	8.5 + 4.8	23.2 + 5.2	0.001	8.8 + 4.9	23.9 + 5.6	0.002
TMB score (Muts/Mbp)	6.0 + 1.6	1.1 + 1.3	2.0 + 2.2	0.9 + 0.8	0.584	0.3 + 1.2	0.9 + 1.1	0.360
AS score (count)^b^	2.0 + 0.9	8.6 + 6.5	0.3 + 0.2	9.7 + 5.1	0.020	2.1 + 2.3	10.3 + 5.6	0.034
CIN score (fraction)	0.2 + 0.1	0.4 + 0.2	0.3 + 0.1	0.3 + 0.1	0.872	0.3 + 1.2	0.3 + 0.2	0.994
HRD score (count)^b^	22.3 + 9.4	33.5 + 7.6	28.2 + 12.1	39.2 + 11.9	0.250	28.2 + 12.5	37.8 + 8.4	0.354
gLOH score (count)^b^	16.1 + 6.2	23.4 + 1.8	19.1 + 6.7	25.4 + 4.1	0.124	19.3 + 6.8	24.6 + 2.3	0.117
LST score (count)^b^	3.0 + 2.6	8.8 + 6.3	4.8 + 4.6	11.1 + 6.8	0.172	4.4 + 4.6	11.8 + 6.3	0.115
TAI score (count)^b^	3.1 + 1.1	1.4 + 1.4	4.3 + 2.5	2.4 + 2.6	0.117	4.5 + 2.5	1.5 + 1.4	0.011
AAS score (count)^b^	7.0 + 5.4	0.3 + 0.5	8.6 + 3.6	3.9 + 5.2	0.081	11.4 + 7.6	1.8 + 1.9	0.019
Average AAS size (Mbp)	2.2 + 1.1	nd	1.7 + 0.3	70.0 + 36.6	0.002	2.7 + 1.0	21.4 + 18.2	0.008

Continuous variables were compared by an unpaired two-tailed Student’s *t* test.

*AAS* amplified aneuploid segments (any size aneuploid segment with normalized copy number total ≥5), *AS* aneuploidy (number of aneuploid chromosome arms), *BC* breast cancer, *CIN* chromosomal instability (fraction of genome in a CNV), *CNV* copy number variant, *DCIS* ductal carcinoma in situ, *ER* estrogen receptor, *gLOH* genomic loss of heterozygosity (≥15Mbp segments of LOH not including centromere), *HRD* homologous recombination deficiency (sum of gLOH + LST + TAI), *LFS* Li Fraumeni syndrome, *LOH* loss of heterozygosity, *LST* large state transitions (2 adjacent segments each ≥10Mbp ≥3Mbp apart not including centromere), *MS* microsatellite status, *Muts/Mbp* mutations per mega base pair, *n/a* not applicable, *nd* not done, *SNV* single nucleotide variant, *TAI* telomeric allelic imbalance (≥11Mbp segments of aneuploidy excluding LOH not including centromere or telomere), *TMB* tumor mutational burden, *TNBC* triple negative breast cancer.

^a^Defined as >2 tumors with an alteration in a gene.

^b^Data presented is counts in tumor.

In order to compare the bi-allelic loss rate of LFS-BC to other LFS cancers, we identified germline *TP53* variants in the pan-cancer TCGA cohort^22^. Putative germline variants in *TP53* (VAF > 30%) were identified in 69 tumors; 24 were classified as PGVs (TCGA-LFS) and 45 as variant of uncertain significance (VUS) or likely benign/benign (LB/B) variants (TCGA-*TP53*-VUS + LB/B) (Fig. 1b and Supplementary Data 3). In the TCGA-LFS cohort, 58% (14/24) of tumors had LOH, and 13% (3/24) had an acquired *TP53* somatic mutation (71% biallelic loss rate). In contrast, only 32% (13/41) of cancers with *TP53* VUS/LB/B variants (*p* < 0.001) had LOH. Among tumors with biallelic loss, the mechanism of biallelic loss was similar in both LFS-BC cohorts and TCGA-LFS cohorts, with copy- neutral LOH being most common (Fig. 1b).

### Oncogenic alterations in LFS versus non-LFS breast cancer

In order to determine whether LFS-BC had any unique oncogenic alterations, we compared LFS-BC to early onset non-LFS-BC from patients negative for PGVs in any cancer risk gene (Supplementary Data 4) and also to non-LFS-BC from TCGA (Supplementary Data 5). Acquired *TP53* oncogenic variants were found in 19% (76/403) of TCGA-BC ER + Her2-, 37% (55/147) of HER2+ and 73% (82/113) TNBC (Supplementary Data 5). Two of 14 (14%) early onset non-LFS ER + Her2- BC had an acquired *TP53* oncogenic variant. The majority of HER2+ (60%, 3/5) and triple negative (88%, 7/8) early onset non-LFS-BC had an acquired *TP53* oncogenic variant (Table 2 and Supplementary Data 2).

The TCGA *TP53* mutant or wild-type *TP53* BC had recurrent oncogenic mutations in several genes including *PIK3CA, GATA3, CDH1, MAP3K1, KMT2C,* and *PTEN* (Supplementary Fig. 2a). In contrast, there were no recurrent oncogenic mutations in LFS-BC (Table 2 and Supplementary Fig. 2b). Early-onset nonLFS-BC had recurrent oncogenic mutations in *PIK3CA* and *GATA3* similar to TCGA; for example, 25–40% (9/27) of early onset nonLFS-BC depending on subtype versus 4% (1/21) of LFS-BC had an oncogenic mutation in *PIK3CA* (Fig. 1a, Table 2, and Supplementary Fig. 2c). We also found no common oncogenic pathways enriched in LFS-BC based on count of mutated genes from the molecular signatures database (MSigDB) (Fig. 1a). Interestingly, mutations in estrogen receptor (ESR) mediated signaling genes were found in 52% (14/27) of nonLFS-BC compared to 14% (3/21) of LFS-BC (*p* = 0.01).

In contrast to infrequent oncogenic mutations, recurrent focal amplifications (normalized CN state ≥4) were seen in LFS-BC, including *ERBB2* (33%, 7/21) and *CDK12* (29%, 6/21) (Fig. 1c, Table 2, and Supplementary Fig. 2d, e). The normalized copy number state of the *ERBB2* locus of all LFS invasive BC was ≥1, all clinically HER2+ LFS-BC had normalized copy number state ≥2 (Fig. 1c). LFS-DCIS ranged from 0.25 to 16. Normalized ratios were <1 in several normal LFS breast tissues, but in each case, this was due to amplification of the entire 17p arm with a diploid *ERBB2* locus. Examination of normalized copy number ratios showed that the majority of LFS-BC had co-amplification of *ERBB2* and *CDK12* (Supplementary Data 2). In summary, LFS-BC nearly always underwent biallelic loss of *TP53*, rarely had oncogenic mutations in other genes, but were enriched for focal amplifications, particularly in *ERBB2* (Table 2).

### Genomic instability in LFS breast cancer

**Fig. 2:**
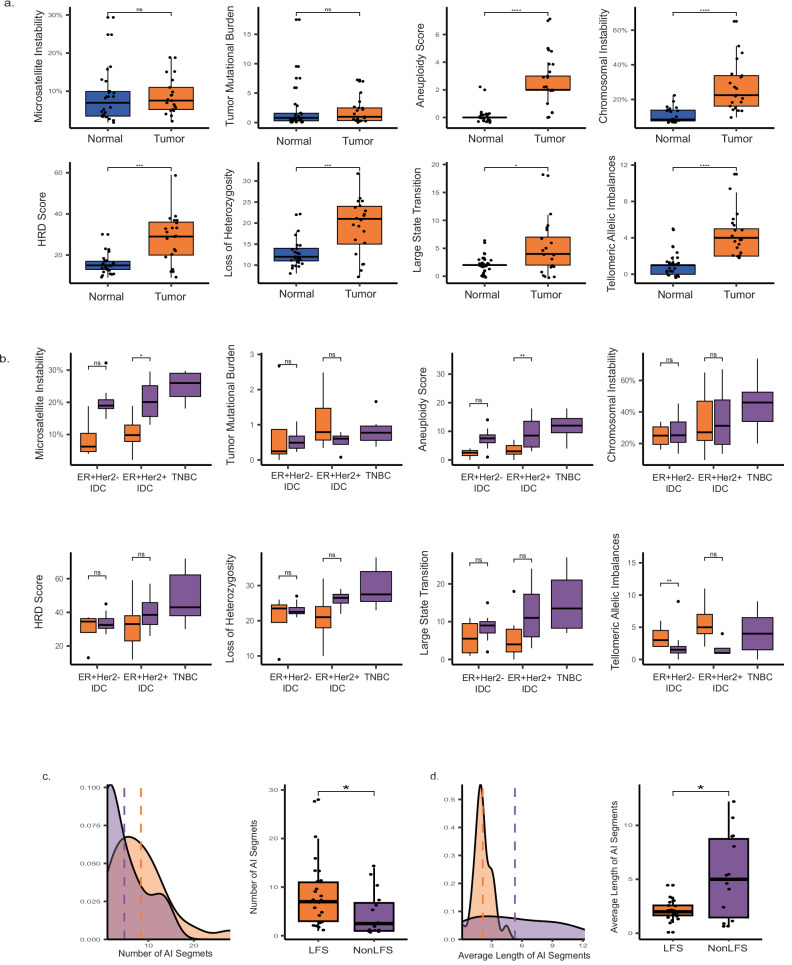
Copy number alterations in LFS-BC compared to early onset non-LFS-BC. **a** Genomic instability measure, including microsatellite instability (MSI), tumor mutational burden (TMB), aneuploidy score (AS), chromosomal instability (CIN) score, homologous recombination deficiency (HRD) score, genomic loss of heterozygosity (gLOH), large state transitions (LST), and non-telomeric allelic imbalances (TAI) comparing tumor from LFS-BC (*n* = 21) to normal LFS breast tissue (*n* = 26). **b** Genomic instability measures stratified by hormone receptor status in LFS-BC (ER + Her2- IDC, *n* = 4 and ER + Her2+ IDC, *n* = 9, orange bars) versus non-LFS-BC (ER + Her2- IDC, *n* = 10, ER + Her2+ IDC, *n* = 4, TNBC, *n* = 6, purple bars). **c** Distribution of the number of amplified (total copy number ≥5) segments of allelic imbalance, aka short amplified aneuploid segments (SAAS), across LFS-BC (*n* = 25, orange curve) and nonLFS-BC (*n* = 14, purple curve) and box plot of the number of amplified AI segments in LFS-BC (orange bars) versus nonLFS-BC (purple bars). **d** Distribution of the lengths of amplified AI segments across LFS-BC (*n* = 25, orange curve) and nonLFS-BC (*n* = 14, purple curve) and box plot of average length of amplified AI segments in LFS-BC (*n* = 25, orange bars) versus non-LFS-BC (*n* = 25, purple bars). AI allelic imbalance, Avg average, CNA copy number alteration, CNt copy number total. ER estrogen receptor, HRD homologous recombination deficiency, HER2 receptor tyrosine-protein kinase erbB-2, No number, IDC invasive ductal carcinoma, LFS Li Fraumeni syndrome, TNBC triple negative breast cancer. For all box plots, the center line is the median, the bounds of the box are the upper and lower quartiles, and the whisker bounds are defined as upper limit = Q3 + (1.5 × IQR), lower limit = Q1 − (1.5 × IQR). Statistical comparisons were performed by Welch’s t-test, ns, non-significant; *p < 0.05; **p < 0.01; ***p < 0.001; and ****p < 0.0001. Source data are provided as a Source data file.

Given p53’s role in maintaining genome integrity, we next compared various measures of genomic instability in LFS-BC to normal tissues. Microsatellite instability (MSI) and tumor mutational burden (TMB) were not significantly different between LFS-BC and normal breast tissues (Fig. 2a); whereas measures of chromosomal-level genomic instability, including whole arm aneuploidy (aneuploidy score), overall chromosomal instability (CIN score), and homologous recombination deficiency (HRD) scores were significantly higher in LFS-BC compared to normal breast tissues. HRD is a composite measure of genomic LOH, large state transitions (LSTs) and segmental allelic imbalances (nTAI); all of which were increased in LFS-BC compared to normal breast tissue. Indeed, we determined the change in genomic instability measures between twenty six pairs of LFS-BC tumors and their normal tissue (Supplementary Fig. 3a), showing that only 27% (7/26) of tumors had higher TMB compared to their matched normal tissue; whereas chromosomal instability measures were uniformly higher in tumors, for example 92% (24/26) and 100% (26/26) of tumor-normal pairs showed enrichment of allelic imbalance segments and aneuploid chromosome arms, respectively (Supplementary Fig. 3b).

We next determined whether these genomic instability measures differed between LFS-BC and early onset non-LFS-BC by hormone receptor (HR) subtype. All genomic instability measures showed the expected trend of increased levels of instability in triple negative versus ER+ nonLFS-BC (Fig. 2b). Interestingly, nearly all genomic instability measures were similar or lower in LFS-BC compared to early onset nonLFS-BC (Fig. 2b and Table 2). In ER + Her2- BC, MSI and aneuploidy scores were significantly lower in LFS-BC versus non-LFS-BC and CIN, HRD and its components were similar across HR subtypes between LFS-BC and non-LFS-BC. Only segmental allelic imbalances (≥15Mbp) were higher in LFS-BC, particularly HER2+ subtypes, compared to non-LFS-BC. The per tumor average number of segments of allelic imbalance (AI) of any size with at least five-fold amplification (amplified allelic segments, AAS) were significantly higher in LFS-BC versus non-LFS-BC (Fig. 2c and Table 2). Interestingly, these aneuploid segments were significantly shorter in LFS-BC than in non-LFS-BC (Fig. 2d and Table 2). and included many oncogenes, such as *ERBB2* (Supplementary Fig. 2). These findings were numerically recapitulated when number of allelic imbalances and AAS were compared between LFS-BC to nonLFS-BC with an acquired *TP53* mutation, and the size of AAS was also significantly smaller in LFS-BC compared to nonLFS-BC with an acquired *TP53* mutation (Supplementary Fig. 4a). In contrast, in sporadic BC from TCGA, aneuploidy score and all HRD measures were higher across all HR subtypes in BC with acquired *TP53* mutations compared to *TP53*-WT tumors (Supplementary Fig. 4b). Comparing premenopausal HER2+ BC with and without acquired *TP53* mutations, aneuploidy score, HRD score and LST score were similar, but segmental allelic imbalances and LOH were higher in p53 mutant compared to p53 WT HER2+ premenopausal BC (Supplementary Fig. 4c). These findings demonstrate that enrichment of all levels of genomic instability occurs with acquired *TP53* mutations across BC; however, there is a specific enrichment of aneuploid segments in premenopausal p53 mutant tumors, specifically short AAS in LFS-BC tumors (Table 2).

### Mutational processes in LFS breast cancer

In order to further explore the copy number signatures unique to LFS-BC, we performed whole genome sequencing of LFS DCIS (*n* = 5), ER + Her2- BC (*n* = 4) and HER2+ BC (*n* = 10) (Supplementary Fig. 5a). Consistent with the enrichment of short aneuploid segments, the most prominent copy number signatures in LFS-BC were chromosomal instability and chromothripsis (CN9/CN5); only two LFS-BC showed evidence of whole genome doubling and diploidy. We next examined the single-nucleotide substitution mutational processes seen in LFS-BC (Supplementary Fig. 5b). All samples had SBS1, and 75% had SBS5 signatures (clock-like signature associated with spontaneous 5-methylcytosine deamination and unknown etiology, respectively). In addition, nearly all LFS-BC had SBS3 (HRD, 10/12) and SBS18 (ROS, 11/12). Other BC associated signatures, SBS2 and SBS13 (APOBEC activity), were not seen in LFS-BC.

### Gene expression patterns in LFS breast cancer

**Fig. 3:**
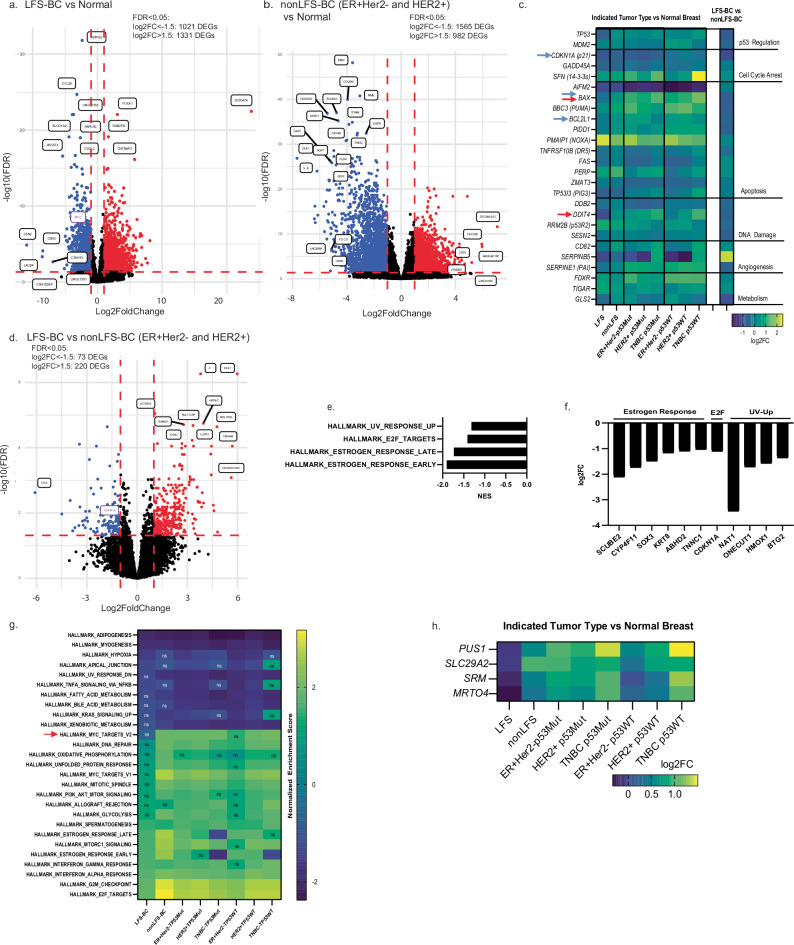
Gene expression profiling in LFS-BC compared to early onset non-LFS-BC. **a** Volcano plot showing differentially expressed genes (DEGs) in LFS-BC (*n* = 16) compared to normal LFS breast tissue (*n* = 12). **b** Volcano plot showing DEGs in nonLFS-BC (*n* = 25) compared to nonLFS normal breast tissue (*n* = 25). **c** Differential expression of canonical p53 targets in the indicated tumor types versus normal breast tissue; LFS-BC (*n* = 16), nonLFS-BC (*n* = 25), TCGA p53mut ER + Her2- nonLFS-BC (*n* = 76), TCGA p53WT ER + Her2- nonLFS-BC (*n* = 327), TCGA p53mut HER2+ BC (*n* = 55), TCGA p53WT HER2+ BC (*n* = 92), TCGA p53mut TNBC (*n* = 82), TCGA p53WT TNBC (*n* = 31). **d** Volcano plot showing DEGs in LFS-BC (*n* = 16) compared to non-LFS-BC (*n* = 25). **e** Hallmark pathways significantly differentially regulated between LFS-BC (*n* = 16) and nonLFS-BC (*n* = 25) (NES < − 2 and FDR < 0.05). **f** Log2 fold change (FC) of significantly regulated genes in these pathways in LFS-BC compared to non-LFS-BC. **g** Normalized enrichment scores for Hallmark pathways in differential gene expression analyses between the indicated tumor type compared to normal breast tissue. Pathways shown were significantly enriched in at least one comparison; nonsignificantly enriched pathways are indicated by “ns”; the red arrow indicates pathway significantly regulated in all comparisons except LFS-BC versus normal. **h** Log2 fold change (FC) of significantly regulated genes in the MYC targets pathway in the indicated tumor type compared to normal breast tissue. BC breast cancer, DEG differentially expressed genes, ER estrogen receptor, FC fold change, FDR false discovery rate, HER2 receptor tyrosine-protein kinase erbB-2, LFS Li Fraumeni syndrome, p53Mut positive for somatic *TP53* mutation, p53WT negative for somatic *TP53* mutation, TNBC triple negative breast cancer. Source data are provided as a Source data file.

To examine whether LFS-BC demonstrates a pattern of gene expression reflective of p53 functional loss, we performed differential expression analysis on bulk RNA-seq from LFS-BC versus LFS normal breast tissues and compared this to differential expression analysis of non-LFS-BC versus non-LFS normal breast tissues from the early onset non-LFS-BC cohort and TCGA-BC, stratified by hormone receptor subtype and somatic *TP53* mutation status. Principal component analysis plots of LFS-BC RNAseq data demonstrated clustering of normal (contralateral and surrounding) and tumor tissue (Supplementary Fig. 6a) and clustering of TNBCs, although other BC subtypes showed less clear clustering (Supplementary Fig. 6b); in addition, no batch effects were seen between LFS-BC and non-LFS-BC data except for non-LFS-TNBC (Supplementary Fig. 6c, d), which were then excluded from downstream differential expression comparisons. We observed 2352 significantly differentially expressed genes (DEGs) in LFS-BC compared to LFS normal breast tissues (adjusted *p*-value < 0.05 and log2fold change > ± 1.5) (Fig. 3a and Supplementary Data 6). Similarly, we observed 2547 DEGs in nonLFS-BC compared to nonLFS normal breast (Fig. 3b and Supplementary Data 7). More DEGs were identified in TCGA-BC hormone receptor subtypes compared to normal breast tissue as expected with larger numbers of samples, but similar numbers of DEGs were identified regardless of somatic *TP53* mutation status (Supplementary Fig. 7 and Supplementary Data 8–13).

We next examined differential expression of a well-curated list of 326 p53 target genes^23^ in LFS-BC, nonLFS-BC and each TCGA subset compared to normal breast tissue, hypothesizing that *TP53* target genes important for BC development would be significantly up-regulated in nonLFS-BC and TCGA-BC versus normal breast tissue but not regulated or down-regulated in LFS-BC versus normal breast tissue. Most p53 target genes were regulated in similar directions and magnitude; notably, however, *BAX* and *DDIT4* were significantly up-regulated in all non-LFS-BC cohorts versus normal breast tissues but either down-regulated or not regulated in LFS-BC versus normal breast tissue (Fig. 3c, red arrows). We next examined these genes in a comparison of LFS-BC versus non-LFS-BC tumors (Fig. 3c and Supplementary Data 14). Expression of p53 apoptosis targets *BAX* and *BCL2L1,* and cell cycle arrest gene *CDKN1A* (p21) were significantly lower in LFS-BC versus non-LFS-BC (blue arrows).

Overall, comparing LFS-BC to non-LFS-BC in a tumor-to-tumor comparison, we found 73 genes that were significantly down-regulated and 220 significantly up-regulated in LFS-BC versus non-LFS-BC (Fig. 3d). MSigDB gene set enrichment analysis (GSEA) identified estrogen response, E2F and UV response as the most significant hallmark pathways that were negatively regulated in LFS-BC vs. non-LFS-BC (Fig. 3e and Supplementary Data 15). The genes that were significantly down-regulated in LFS-BC versus non-LFS-BC in these pathways included tumor suppressors, such as *CDKN1A* and *BTG2*. (Fig. 3f). We next performed GSEA on the LFS-BC, non-LFS-BC and TCGA cohort BC versus normal breast tissues (Supplementary Fig. 8 and Supplementary Data 16). Most significantly up and down regulated Hallmark pathways in LFS-BC were similarly regulated in nonLFS-BC and all TCGA-BC (ER + Her2- and HER2+) subtypes (Fig. 3g and Supplementary Fig. 8). Notably, however, MYC pathway was significantly up-regulated in most nonLFS-BC and TCGA cohorts and not up-regulated in LFS-BC, four genes from this pathway were significantly downregulated in LFS-BC versus normal breast tissue whereas those genes were significantly upregulated in non-LFS BC versus normal (Fig. 3h). In order to incorporate the directionality of RNA expression differences between LFS-BC and nonLFS-BC, we conducted an analysis with ingenuity pathway analysis (IPA) software (QIAGEN Inc., https://digitalinsights.qiagen.com/IPA). This analysis demonstrated that the p53 pathway was significantly altered in LFS-BC, with key genes regulating cell cycle arrest, *CDKN1A*, and apoptosis, *BAX*, being down-regulated in LFS-BC and genes involved in suppressing angiogenesis being up-regulated (Supplementary Fig. 9).

### Tumor microenvironment in LFS breast cancers

**Fig. 4:**
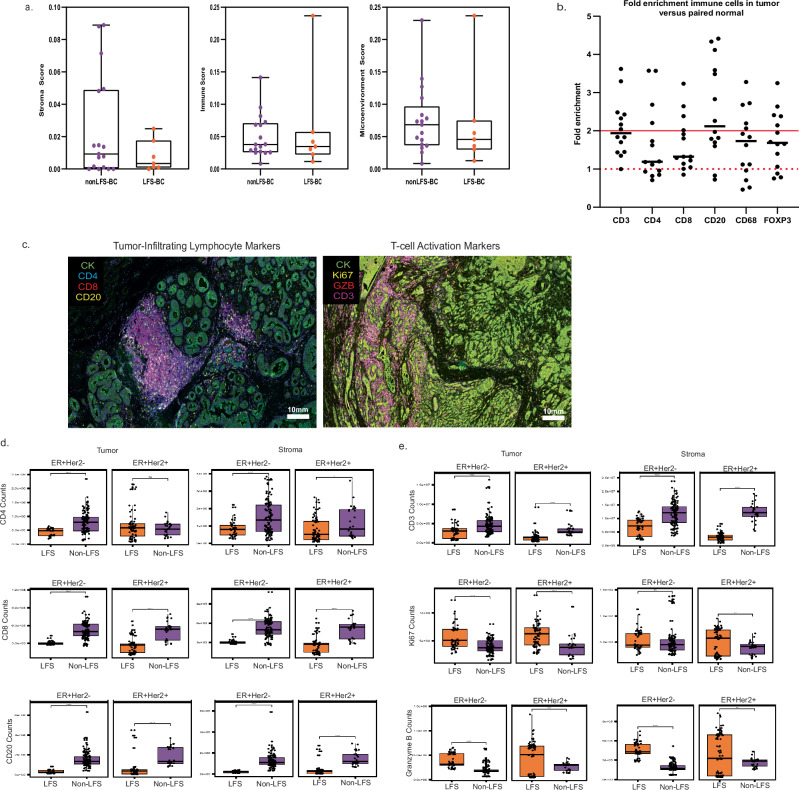
Immunological profiling in LFS-BC compared to early onset non-LFS-BC. **a** xCell analysis of RNASeq data showing stroma, immune and microenvironment scores from invasive cancers from LFS-BC (*n* = 7) and early-onset non-LFS (*n* = 17). **b** Single stain IHC in LFS-BC compared to normal LFS breast tissue pairs (*n* = 14), fold enrichments in tumor versus normal are shown as a dot plot with a line at median. **c** Representative images of multiplex immunohistochemistry (IHC) results staining for tumor- infiltrating lymphocyte (TIL) and T-cell activation (ACT) panels. Scale bar = 100 microns. **d** Quantification of CD4+, CD8+, and CD20+ cell counts within tumor and stromal compartments from individual fields-of-view in LFS-BC (ER + HER2- IDC, *n* = 40; ER + HER2+ IDC, *n* = 76) and non-LFS BC (ER + HER2- IDC, *n* = 128; ER + HER2+ IDC, *n* = 28). **e** Quantification of granzyme B+, Ki67+, and CD3+ cell counts within tumor and stromal compartments from individual fields-of-view in LFS-BC (ER + HER2- IDC, *n* = 40; ER + HER2+ IDC, *n* = 76) and non-LFS BC (ER + HER2- IDC, *n* = 128; ER + HER2+ IDC, *n* = 28). ACT T-cell activation, CK cytokeratin, BC breast cancer, ER estrogen receptor, HER2 receptor tyrosine-protein kinase erbB-2, LFS Li Fraumeni syndrome, TIL tumor-infiltrating lymphocytes, TNBC triple-negative breast cancer. For all box plots (**a**, **d**, **e**) the center line is the median, the bounds of the box are the upper and lower quartiles, and the whisker bounds are defined as upper limit = Q3 + (1.5 × IQR), lower limit = Q1 − (1.5 × IQR). Statistical comparisons were performed by Welch’s t-test, ns, non-significant; *p < 0.05; **p < 0.01; ***p < 0.001; and ****p < 0.0001. Source data are provided as a Source data file.

In BC, the presence and activity of tumor-infiltrating lymphocytes have significant implications for prognosis and treatment^24^. We therefore investigated the tumor immune microenvironment (TIME) in LFS-BC compared to non-LFS-BC and the normal breast tissues from each group using the RNASeq data. CIBERSORT (Supplementary Data 17) was used to determine differences in the proportion of immune cell populations. MCPCounter (Supplementary Data 18) and xCell (Supplementary Data 19) were used to estimate relative abundances of immune cell populations. We observed prominent variation within each group for all TIME cell types (Supplementary Fig. 10). We observed no differences in composite stroma, microenvironment or immune scores in invasive LFS-BC compared to early onset non-LFS-BC (Fig. 4a).

We next performed single immune cell marker IHC on the LFS-BC compared to the surrounding normal tissue. Comparing an LFS breast tumor to its surrounding normal demonstrated that CD3+, FoxP3+ Tregs, CD68+ and CD20+ cells were approximately two-fold enriched in the majority of tumor-normal pairs compared to SNBT (Fig. 4b).

We assayed our LFS-BC versus early-onset non-LFS-BC samples using two mIF panels, which tested the levels of specific immune cell subpopulations (tumor infiltrating lymphocyte [TIL], T-cell activation [ACT]) (Fig. 4c). The levels of the markers were selectively measured in three different spatial tumor tissue compartments using fluorescence co-localization strategies^25^ including: the cancer-cell nests (cytokeratin [CK]+ pixels/area or tumor compartment), the non-tumor/stromal cells (CK- pixels/area or stromal compartment) and the entire tumor tissue, including both malignant and non-malignant cells (dilated DAPI+ pixels/area).

We first examined whether our method recapitulated a difference in immune cell presence in non-LFS. We found early onset non-LFS TNBC had significantly higher densities of CD8+, CD4+, and CD20+ cells in their stroma compared to ER + Her2- and HER2+ non-LFS-BC with similar levels intratumorally (Supplementary Fig. 11a–c). We discovered that compared to early onset non-LFS-BC, LFS-BC had significantly lower tumor-infiltrating CD8+ and CD20+ cells in both HER2- and HER2+ cancers (Fig. 4d). In addition, CD4+ TIL levels were also lower in LFS-BC than in non-LFS but only in HER2-tumors. These differences occurred throughout compartments (Supplementary Fig. 12a).

We next assayed for T-cell activation using CD3, granzyme B, and Ki-67. As expected, CD3 and granzyme B levels were correlated, and Ki67 was higher in tumor than stroma (Supplementary Fig. 12b). While LFS-BC had lower densities of CD3+ cells compared to early-onset non-LFS-BC, LFS-BC had significantly higher levels of granzyme B and Ki-67 (Fig. 4e), indicating higher levels of activated cytotoxic T-cells in LFS-BC. This was found in both HER2- and HER2+ comparisons and across compartments (Supplementary Fig. 12c).

In order to validate these results in a larger cohort, we additionally evaluated 17 LFS-BC and 24 nonLFS-BC from a second institution, Memorial Sloan Kettering Cancer Center (MSKCC). The MSKCC LFS-BC cohort was older, and tumors were lower stage compared to the Penn LFS-BC cohort; the distribution of hormone receptor status and grade were similar (Supplementary Data 20). The non-LFS-BC cohorts had similar clinical and pathological features (Supplementary Data 20). Combining these cohorts recapitulated the findings of lower CD8+ and CD20+ cells and higher levels of Ki67 and granzyme B in LFS-BC versus non-LFS-BC tumors (Supplementary Fig. 13a–d).

### Evolution of genomic, transcriptomic, and immune cell changes in DCIS compared to invasive LFS breast cancer

**Fig. 5:**
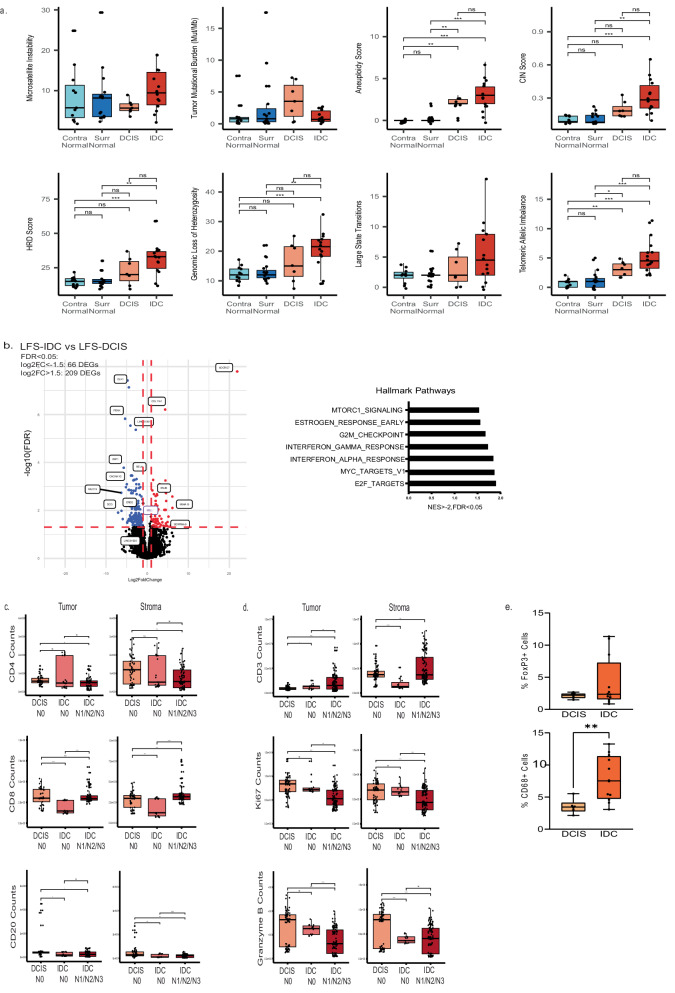
Genomic, transcriptomic, and immunological features of LFS-DCIS versus LFS-IDC. **a** Genomic instability measures including microsatellite instability (MSI), tumor mutational burden (TMB), aneuploidy score, chromosomal instability (CIN) score, homologous recombination deficiency (HRD) score, genomic loss of heterozygosity (gLOH), large state transitions (LST) and non-telomeric allelic imbalances (TAI) in normal LFS breast tissue (contralateral, *n* = 11; surrounding, *n* = 15), LFS-DCIS (*n* = 7), and LFS-IDC (*n* = 14). *p*-values in Source data file. **b** Differentially expressed genes (DEGs) in LFS-DCIS (*n* = 8) versus LFS-IDC (*n* = 15). Red, FDR < 0.05 and log2(FC) < − 1.5 or >1.5; blue, FDR < 0.05 and log2(FC) = −1.5–1.5. Involvement of these DEGs in Hallmark biological processes. **c** Quantification of CD4+, CD8+, and CD20+ cell counts within tumor and stromal compartments from individual fields-of-view in LFS-DCIS (*n* = 53) and LFS-IDC (node-negative, N0, *n* = 21; node-positive, N1/N2/N3, *n* = 78) represented as median **±** SD. **d** Quantification of granzyme B+, Ki67+, and CD3+ cell counts within tumor and stromal compartments from individual fields-of-view in LFS-DCIS, *n* = 53 and LFS-IDC; node-negative, N0, *n* = 21; node-positive, N1/N2/N3, *n* = 78). **e** Quantification of single-stain IHC for FoxP3⁺ and CD68⁺ cells in LFS-associated DCIS (*n* = 7) and IDC (*n* = 12). ACT T-cell activation, CK cytokeratin, DCIS ductal carcinoma in situ, ER estrogen receptor, FC fold change, HER2 receptor tyrosine-protein kinase erbB-2, IDC invasive ductal carcinoma, LFS Li Fraumeni syndrome, SD standard deviation, TIL tumor-infiltrating lymphocytes, TNBC triple-negative breast cancer. For all box plots (**a**, **c**–**e**), the center line is the median, the bounds of the box are the upper and lower quartiles, and the whisker bounds are defined as upper limit = Q3 + (1.5 × IQR), lower limit = Q1 − (1.5 × IQR). Statistical comparisons were performed by Welch’s t-test in (**a**–**d**) and two-tailed unpaired Student’s *t* test in (**e**), ns, nonsignificant; *p < 0.05; **p < 0.01; *** *p* < 0.001; and ****p < 0.0001. Source data are provided as a Source data file.

Invasive breast cancers are thought to arise through a phase of DCIS; therefore, we examined the genomic, transcriptomic and immune cell differences between DCIS and IDC in LFS-BC. When LFS-BC were stratified into DCIS versus IDC, all chromosomal-level genomic instability measures, except LSTs, were statistically higher in both DCIS and IDC compared to normal tissue. Genomic instability was similar although trended towards an increase in the IDC versus DCIS tumors (Fig. 5a). LFS DCIS and IDC had significant differences in gene expression profile (Supplementary Data 21). We observed 275 DEGs in LFS-IDC compared to LFS-DCIS (Fig. 5b); with a number of oncogenic pathways significantly up-regulated in LFS-IDC compared to LFS-DCIS (Fig. 5b and Supplementary Data 22).

When we compared the TIL panel in LFS DCIS versus LFS IDC stratified into tumors that were node negative versus node positive, there were no consistent differences in T or B-cells (Fig. 5c). LFS-DCIS had significantly higher CD8+ T cell levels compared to node negative IDC but not node positive (Fig. 5c). These results were recapitulated with single immune marker IHC for CD3, CD4, CD8, and CD20 in a subset of samples (Supplementary Fig. 14a), and results were similar in the DAPI compartment (Supplementary Fig. 14b). We next quantified the activity of the infiltrating immune compartments using the ACT activity assay in LFS-DCIS versus LFS-IDC. We found that there were significantly higher Ki67+ and granzyme B in LFS-DCIS as compared to LFS-IDC but fewer CD3+ cells (Fig. 5d). Furthermore, single immune marker staining in a subset of samples demonstrated a nominal increase in tumor supportive FoxP3+ T-regulatory cells and a significant increase in CD68+ macrophages in LFS-DCIS versus LFS-IDC (Fig. 5e). These findings suggest that LFS-IDC develop mechanisms for adaptive immune evasion during progression.

## Discussion

In this study, we leveraged patients with germline *TP53* PGVs to identify the unique genomic, transcriptomic and immunologic characteristics of breast tumor formation induced by mutant p53. Breast tumors in LFS patients uniformly underwent biallelic loss of *TP53* and, compared to age-matched early onset non-genetically driven BC, rarely accumulated other oncogenic mutations. Instead, LFS-BC accumulated short amplified aneuploid segments, which we term “SAAS”. LFS breast tumors specifically lost transactivation of apoptosis genes versus other p53 target genes when compared to normal breast tissue. Further, LFS-BC had a unique immune infiltrate with overall lower CD8+ T-cells, but higher activated cytotoxic T-cells. Finally, progression of *TP53* mutant in situ to invasive tumors was not associated with significant genomic changes but was associated with reduced TILs, loss of cytolytic T cells and prominent upregulation of CD68+ tumor-associated macrophages.

Our data suggest that *TP53*-related breast tumor formation in the germline state requires biallelic loss of *TP53* as a first step in tumor formation. This is consistent with data from pediatric tumors in LFS patients^17^, which showed that *TP53* biallelic loss occurs early in tumor formation. Our comprehensive analysis of oncogenic mutations, copy number changes and mutational signatures further shows that biallelic *TP53* loss does not lead to the accumulation of single-nucleotide variants nor is marked by specific single-base pair substitution signatures, likely explaining the absence of other oncogenic mutations in LFS-BC. Instead, we observed that while all levels of genomic instability accumulated in LFS-BC compared to normal LFS breast tissue, only SAAS events were higher in LFS-BC compared to non-LFS-BC. Our copy number mutational signature results support the hypothesis that development of aneuploid segments is an important early step in tumor development, as we found that chromothripsis (i.e., CN5) and focal LOH-diploid or chromosomal Instability (CN9) were common in LFS-BC. This contrasts with somatic *TP53* mutations, which are associated with increases in all levels of genomic instability compared to BC without acquired *TP53* mutations. Other types of genomic-level alterations, such as genome doublings, were not observed. Taken together, mutant p53-driven SAAS formation is likely responsible for the observation that HER2 amplification is a common feature of LFS-BC.

In contrast to our results in BC, a pancreatic cancer *TP53* mutant model demonstrated that *TP53* LOH led first to deletion events followed by genome doubling and amplifications^26^. We found no evidence of genome doubling by copy number signature analysis in LFS-BC. It is known that estrogen stimulates DNA double-strand breaks in cells as part of its metabolism and proliferative effect^27^ and can lead to translocation-bridge mediated focal amplifications^28^. Genomic and expression profiling in our analysis showed that LFS-BC had less frequent activating alterations in the *ESR1* pathway and, consistently, failed to upregulate estrogen-responsive pathways as occurred in non-LFS-BC. Therefore, it is possible that p53 dysfunction does not rely on aberrant estrogen signaling as would be consistent with analyses in sporadic metastatic breast cancers^29^. In addition, it is possible that LFS-BC have a different program of genome instability in estrogen-responsive tissues compared to other tissues. Other groups have shown that HRD occurs in *TP53* mutated samples irrespective of HR gene alterations^30^; however, we did not observe higher HRD in LFS-BC compared to non-LFS-BC, and the DNA damage response pathway were not dysregulated. This again shows that tumorigenesis follows distinct, likely tissue-specific, pathways when p53 dysfunction is the initiating event versus a later event in tumor evolution.

Another intriguing finding of our work is that LFS-BC have lower lymphocyte infiltration as compared to non-LFS BC, a finding that is consistent with prior studies showing reduced TILs in LFS-BC^9,14^, likely due to higher levels of aneuploidy in LFS-BC^31–33^. In addition, patients with LFS may have a higher germline epitope burden, which has recently been suggested to lead to increased immunoediting and immune-depleted tumors^34^. Our finding of enrichment of FoxP3+ Tregs and CD68+ macrophages is consistent with a prior CIBERSORT analysis of TCGA data showing that acquired *TP53* mutations promoted an immune suppressive environment with infiltration of Tregs, T helper cells and M0-type macrophages^35^. Further, our finding that immune infiltration decreases with progression from DCIS to IDC is consistent with prior work in sporadic BC^36,37^, suggesting the necessity of promoting an immune suppressive environment is consistent between LFS and non-LFS patients. It is possible that these observations are related to copy number burden or aneuploidy; however, our sample size limited this analysis.

Given a lack of other oncogenic processes, outside of reactivating p53 function in breast tissues, genomic sequencing alone does not suggest clear pathways for specific targeted treatment. However, our analyses of mutational signatures and immunological differences in LFS-BC versus non-LFS BC may suggest some potentially targetable avenues for treatment. For example, nearly all LFS-BC show signatures of HRD (10/12), which suggests that treatment with PARP inhibitors could be beneficial in LFS-BC patients^38^. In addition, our previous work on modeling mutant *TP53* fitness predicted that selective pressure from T-cells may exist in LFS due to intrinsic trade-offs, with the selective benefit mutant p53 provides cancer cells, implying immunotherapeutic interventions may be possible^39^. Indeed, TNBC, which typically has mutant p53, is partially sensitive to immune checkpoint blockade^40^. While LFS-BC have lower CD8 infiltration compared to non-LFS-BC, markers of T-cell activation were higher, suggesting a possible benefit for an anticancer vaccine to boost tumor-specific antigen responses.

There are limitations to our data in that, while the largest described cohort of LFS-BC from a genomics and immunological perspective, this sample set is still small, limiting comparisons within hormone receptor and stage subtypes. In addition, genomic analysis is limited from FFPE tissues, and additional studies, particularly at the single-cell level from fresh tissues, will be necessary to confirm these findings and explore the cell intrinsic and extrinsic mechanisms of tumor formation initiated by p53 loss.

In conclusion, breast cancer is the most common cancer in females with LFS, occurring 30 years earlier than sporadic breast cancers. Our data suggest that breast tissues with a germline *TP53* pathogenic variant first undergo biallelic loss of *TP53* to a fully mutant p53 state, resulting in accumulation of SAAS, possibly selecting for *ERBB2* amplification. Mutant p53 leads to transcriptional reprogramming away from an apoptotic state. As LFS-BC progresses from DCIS to IDC, a tumor-promoting immune evasive or tolerogenic environment is established. Our study represents a comprehensive characterization of human p53-driven breast tumors and suggests unique mechanisms of treatment.

## Methods

### Cohort ascertainment

Patients with *TP53* PGVs were ascertained from the Penn/CHOP Li Fraumeni syndrome/TP53 Biobank (NCT04367246). Acquisition of patient blood and tumor samples was approved by the Institutional Review Boards of the University of Pennsylvania and Children’s Hospital of Philadelphia (CHOP) (Penn IRB#834147/CHOP IRB#18-015810). Informed consent was obtained from each participant for the use of their samples and clinical data in genetic and immunologic studies. Inclusion criteria for LFS-BC were (1) clinically confirmed *TP53* PGV as per ClinVar assessment, including those with conflicting interpretations; (2) available pathology slides and/or tumor blocks; and for sequencing (3) available germline blood DNA; (4) assigned female at birth (AFAB). Only AFAB individuals were studied, as there is no known association of male breast cancer with LFS. Only primary breast tumors were included. Chart review was performed via the associated research database and the Penn Medicine electronic health record for clinical characteristics, tumor information, and treatment information. Ninety-three LFS-BC patients with 130 primary breast tumors were identified in the Penn Medicine cohort. Sixty-one cases had available slides for review, 56 cases (43%) had identifiable material and a matched germline blood specimen. Specimens from 28 LFS-BC (50%) from 22 patients were obtained, of which SNBT and CNBT were available for analysis in 16 cases and 11 cases, respectively (Supplementary Fig. 1 and Supplementary Data 1). All human tissue samples have been conserved for further analysis in the laboratory of the corresponding author at the University of Pennsylvania.

An early-onset non-LFS-BC cohort was created from patients who have provided written consent to Penn Medicine Biobank (PMBB), under IRB protocol #813913 and #817977 (Penn Medicine BioBank: Tissue and/or Blood Collection)^41,42^. Acquisition of patient blood and tumor samples was approved by the Institutional Review Board of the University of Pennsylvania (Penn IRB#832122). Patients were included if they had (1) BC diagnosed age < 50, (2) undergone germline whole exome sequencing (WES) and had no identified PGV in *TP53* or any of 44 other autosomal dominant cancer susceptibility genes (Supplementary Data 4) and (3) cancer clinical data present in the Penn Medicine Cancer Registry. One hundred ninety-eight early-onset non-germline genetic-driven BC patients with 209 primary breast tumors were identified in the PMBB cohort. Seventy-four cases (37%) had not received pre-operative chemotherapy, had identifiable pathological specimens and a matched germline blood sample. Twenty-nine breast tumors (39%) from 28 patients were obtained for analysis (Supplementary Fig. 1 and Table 1). All human tissue samples have been conserved for further analysis in the Penn Medicine Biobank.

Tissue and plasma samples from LFS-BC and non-LFS-BC at Memorial Sloan Kettering Cancer Center (MSKCC) were identified for the validation cohort. Patients provided written informed consent to either Genomic Profiling in Cancer Patients (MSKCC IRB#12-245) or Storage and Research Use of Human Biospecimens (MSKCC IRB#06-107), which allows for the collection of patient blood and tissue samples. Analysis of these samples was reviewed and approved under A Translational Research Program to Test the Immunological Recognition of Tumors Produced in Li-Fraumeni Patients (MSKCC IRB#21-232). These tumors were examined by a trained pathologist team under research protocols 12-245 or 06-107. Inclusion criteria for the LFS-BC cohort were individuals with germline mutations in *TP53* with a diagnosis of breast cancer. Inclusion criteria for the non-LFS-BC cohort were individuals without germline *TP53* mutations with a diagnosis of breast cancer.

For both cohorts, positivity for estrogen receptor (ER), progesterone receptor (PR) and erbb-2 (HER2) were obtained from clinical pathological reports, which follow the College of American Pathologists (CAP) reporting^43,44^.

### Sample preparation of sequencing libraries

For the Penn Medicine cohorts, formalin-fixed paraffin-embedded (FFPE) tumor blocks from LFS and non-LFS-BC cases were sectioned and stained with hematoxylin and eosin (H&E) to ensure that sections of over 70% of in situ or invasive tumor were used for DNA and RNA extraction. Selected tumor areas of slides were macro-dissected and prepared for sequencing as previously described^45^. Contralateral and surrounding normal tissue sections were stained with H&E to ensure sections of normal tissue were used for DNA and RNA extraction. Germline DNA from blood or saliva was extracted using standard protocols. Libraries of tumor, normal tissue and matched germline DNA were prepared using KAPA Hyper Prep Kit (Roche Diagnostics, Branchburg, NJ). Libraries were subjected to targeted next-generation sequencing (NGS) using a custom targeted hybrid capture- based probe set including 512 cancer genes^46^ along with the xGen CNV Backbone Panel using xGen Hybridization Protocol (Integrated DNA Technologies, Coralville, IA). Tumors were sequenced on an Illumina Nova-Seq (Illumina, Madison, WI). LFS breast tumors were additionally subjected to whole genome sequencing on an Illumina Nova-Seq (Illumina, Madison, WI). RNA from LFS-BC, surrounding and contralateral normal LFS breast, non-LFS-BC, and surrounding normal non-LFS breast samples were prepared using the Stranded Total RNA Prep with Ribo-Zero Plus (Illumina, Madison, WI), creating cDNA libraries. cDNA libraries were sequenced on an Illumina Nova-Seq (Illumina, Madison, WI). For whole genome sequencing, LFS-BC tumor DNA underwent Library Preparation EF 2.0 with Enzymatic Fragmentation and Twist Universal Adapter System (Twist Bioscience, San Francisco, CA). DNA is stored at −20 °C, RNA at −80 °C, and cDNA libraries at −20 °C. Samples were not destroyed during analysis and are retained in the laboratory for future research. Availability of samples is subject to institutional approval; requests should be directed to the corresponding author.

### Bioinformatics for targeted next-generation sequencing data

FASTQ files from sequencing of 22 LFS-BC, 26 LFS normal breast tissues, 27 non-LFS-BC, and patient-matched blood or saliva normal were aligned to human genome version 19 (hg19) using BWA-mem version 0.7.17^47^. Sequencing quality control removed one LFS-BC and one LFS surrounding normal tissue for a total of 21 LFS-BC, 15 surrounding normal LFS breast tissue, 11 contralateral normal LFS breast tissue, and 27 non-LFS-BC with targeted NGS data (Supplementary Fig. 1 and Supplementary Data 1). Germline variants were called from BAM files using the genome analysis toolkits (GATK) HaplotypeCaller version 3.7^48^. Individual tumor sequencing data and normal breast tissue sequencing data were matched to sequencing data from the same patient’s lymphocytes. Somatic copy number variations were called using both Sequenza version 3.0.0^49^ and CNVKit version 0.9.9^50^. Using CNVKit copy number segments as input, HRDex version 0.0.0.9^51^ was used to determine HRD scores, including segments of LSTs, genomic loss of heterozygosity (LOH) and non-telomeric allelic imbalance (nTAI) and aneuploidy scores. Copy number burden as a measure of overall chromosomal instability (CIN) was calculated as the percentage of bases measured that were in an altered copy number state. Somatic single-nucleotide variants (SNVs) were called using Mutect2 version 4.1.2^52^. Somatic SNVs were annotated using ANNOVAR version 2018-04-16^53^ and OncoKB^54^. TMB was calculated as the number of non-oncogenic SNVs with a depth greater than 20 and an allelic balance greater than 0.05 divided by the number of bases captured and multiplied by 100000. MSI scores were calculated using MSISensor version 0.6^55^.

### Determination of locus-specific loss of heterozygosity for *TP53*

The presence of genomic locus-specific LOH in *TP53* was determined for each LFS-BC and normal breast tissue sample using a combination of somatic variant allele frequency (VAF) of the PGV as determined by Varscan2, tumor purity as determined by Sequenza, and copy number at the genomic locus as determined by Sequenza. To correct for tumor purity, a minimum expected LOH VAF was calculated as a function of the tumor purity: E = P + (1 − P)/2, where E = expected LOH VAF of 50% and P = tumor purity. If the observed somatic VAF exceeded the expected LOH VAF and/or the “B allele” copy number was zero, the variant was classified as having locus-specific LOH. The presence of second somatic hits in *TP53*, depending on germline PGV, were identified by Mutect2 and Varscan2. Locus-specific LOH genomic calls in *TP53* were harmonized with p53 immunohistochemistry (IHC) (below) to determine overall bi-allelic loss status for each LFS-BC and normal breast tissue sample.

### Bioinformatics for whole genome sequencing (WGS)

FASTQ files of 19 LFS-BC WGS were aligned to hg19 using BWA-mem^47^. Sequencing quality control removed three tumors. Somatic copy number variations were called using CNVKit version 0.9.9^50^. A panel of ethnicity and sex matched normal tissues sequenced on the same machine served as a reference set for CNVKit. Using CNVKit copy number segments as input, HRDex was used to determine HRD scores and aneuploidy scores. Copy number burden was calculated as the percentage of bases measured that were in an altered copy number state. Somatic single-nucleotide variants were called using Mutect2. A panel of ethnicity and sex matched normals sequenced on the same machine was used to filter out suspected germline variants and technical artifacts. Gnomad was additionally used to filter for common germline variants. TMB was calculated as the number of non-oncogenic SNVs with a depth greater than 20 and an allelic balance greater than 0.05 divided by the number of bases captured and multiplied by 100,000. FitMS version 2.3.0^56^ was used to determine the proportion of single-nucleotide and copy-number variants attributable to known COSMIC mutational signatures.

### Bioinformatics for RNA sequencing

FASTQ files from 16 LFS-BC, 12 LFS normal breast tissue, 25 non-LFS BC, and 25 non-LFS normal breast tissue were aligned to hg19 using STAR Align version 2.7.8a^57^. Genes were counted using HTSeq-Count version 2.0.3^58^. Differential expression was performed using DESeq2 version 1.38.3^59^. GSEA was performed using Broad GSEA version 4.2.3^60^. Immune cell type proportions were estimated using CIBERSORTx^61^, MCPCounter^62^ and xCell^63^.

### Analysis of The Cancer Genome Atlas (TCGA) cohorts

To create a TCGA-LFS cohort, carriers of putative germline *TP53* variants were identified from the TCGA pan-cancer analysis^22^. Tumor and matched normal DNA BAM files were downloaded from the Genomic Data Commons (GDC) using a National Center for Biotechnology Information Genotypes and Phenotypes Database (NCBI dbGaP phs000178) approved protocol (#21931) and underwent quality control to ensure the *TP53* variant was found at the expected heterozygous frequency in the normal BAM file and variant classification was performed using the American College of Medical Genetics (ACMG) specific guidelines^64^.

Analysis of TCGA non-LFS-BC whole exome sequencing (WES) was performed as previously described^45^; briefly, all tumor/normal BAM files were downloaded from GDC and underwent the same variant calling and classification pipeline as above; samples with cancer risk gene PGVs (Supplementary Data 4) were excluded. For the TCGA non-LFS-BC RNAseq analysis, FASTQ files were downloaded from GDC and underwent the same bioinformatics analysis as above. Clinical data were downloaded from cbioportal^65^. TCGA nonLFS-BC were categorized as ER + Her2-, HER2+, and TNBC based on TCGA clinical data and sub-grouped by presence or absence of *TP53* acquired somatic mutation (Supplementary Data 5). The TCGA nonLFS-BC cohort consisted of ER + Her2- (*n* = 263 wild type *TP53* (*TP53* WT), *n* = 82 mutant *TP53* (*TP53* Mut)), HER2+ (*n* = 68 *TP53* WT, *n* = 56 *TP53* Mut) and TNBC (*n* = 12 *TP53* WT, *n* = 78 *TP53* Mut), and *n* = 113 normal breast tissue samples for RNAseq.

### p53 and immune marker immunohistochemistry

For single marker immunohistochemistry (IHC), five-micron sections of formalin-fixed paraffin-embedded tissue were stained using the following anti-human antibodies: DO-7 (p53), C8 (CD8), L26 (CD20), KP1 (CD68) (Dako, Carpinteria, CA); LN10 (CD3) (Leica Microsystems, San Francisco, CA); RM (CD4) (Biocare Medical, Pacheco, CA), 206D (FoxP3) (Biolegend Antibodies, San Diego, CA). Antibody dilutions were as follows: DO-7 (1:60), LN10 (1:1), RM (1:1), C8/144B (1:40), L26 (1:1), KP1 (1:1), 206D (1:100). Staining was performed on a Leica Bond-IIITM instrument using the Bond Polymer Refine Detection DS9800 System (Leica Microsystems, San Francisco, CA. Heat-induced epitope retrieval was done for 20 min with ER1 solution (Leica Microsystems, San Francisco, CA). All experiments were done at room temperature. Slides were washed three times between each step with bond wash buffer or water. To quantify the single stain IHC, we used QuPath v0.4.3^66–68^ on high-resolution IHC brightfield images. We selected the regions of interest within the tissue (normal versus tumor) based on the pathologist-marked H&E slide, QuPath algorithms to identify single cells and quantification of staining intensity and calculation of percent positive cells within each area of tissue. In QuPath, we first annotated the main region of interest, tumor/normal, as annotated by Breast pathologists. Then we used the cell detection command to identify cells within the tumor boundary. Next, we annotated regions containing different cell types (tumor/normal cells/stroma), trained a cell classifier based on these annotations, saved the classifier, and applied it to the cells. Finally, we applied intensity classification to assess staining and view the results (Supplementary Fig. 15).

### Multiplex immunofluorescence (mIF)

Slides for mIF were cut at 5 microns from LFS-BC and non-LFS-BC samples. Each tissue slide was curated by a pathologist to determine if the slide contained tumor tissue. We conducted two independent mIF assays on tumor slides: TIL (tumor infiltrating lymphocytes) and ACT (activation). The mIF panels were previously standardized for FFPE samples and performed as previously reported^25,69,70^. TIL assay queried for CD8+, CD4+, CD20+, cytokeratin (CK), and DAPI. Cancer cells were determined by CK and DAPI. The ACT assay queried for granzyme B (GrzmB), Ki67+, CD3+, CK, and DAPI. Granzyme B positivity indicates cytolytic activity and active T-cell-mediated killing, Ki67+ is a marker for cell proliferation, and CD3+ tests for the presence of T-cells. A total of 57 samples were tested with the TIL and ACT assays (comprising 7 spontaneous ER+/PR+, 16 spontaneous TNBC, and 33 LFS-BC).

Whole-tissue sections slides were deparaffinized and subjected to antigen retrieval using EDTA buffer (Sigma-Aldrich) pH = 8.0, and boiled for 1 h at 96 °C in a pressure-boiling container (PT module, Lab Vision). Slides were then incubated with dual endogenous peroxidase block (#S2003: Dako) for 10 min at room temperature. Non-specific antigens were blocked by a 30-min incubation in 0.3% BSA in TBST. The sequential multiplexed immunofluorescence protocol was performed using isotype-specific primary antibodies. The primary antibodies for the tumor infiltrating lymphocytes was performed to detect epithelial tumor cells (Cytokeratin Alexa-488 conjugated, clone AE1/AE3, eBioscience), helper T-cells (CD4 Rabbit monoclonal IgG, 1:100, clone SP35, SpringBio), cytotoxic T-cells (CD8 Mouse monoclonal IgG1k, 1:250, clone C8/144B, Dako), and B-cells (CD20 Mouse monoclonal IgG2a, 1:150, clone L26, Dako) as reported^71,72^. The activation panel was performed to detect epithelial tumor cells (Cytokeratin Alexa-488 conjugated, clone AE1/AE3, eBioscience), T-cells (CD3 Rabbit monoclonal IgG, 1:100, clone SP7, Novus), cell proliferation (Ki-67 Mouse Monoclonal IgG1, 1:100, clone MIB-1, Dako) and T-cell activation (granzyme B mouse monoclonal IgG2a, clone 4E6, Abcam as reported^25^. Secondary antibodies and reagents used were anti-rabbit Envision (K4003, Dako), with biotinylated tyramide/Streptavidine-Alexa750 conjugate (PerkinElmer), anti-mouse IgG1k antibody (1:100, eBioscience) with Cy3-tyramide (PerkinElmer) and anti-mouse IgG2a antibody (1:200, Abcam) with Cy5-tyramide (PerkinElmer). Nuclei were highlighted using 4’,6-diamidino-2-phenylindole (DAPI). All multiplexed panels included elimination of residual horseradish peroxidase activity between incubations with secondary antibodies by exposing the slides twice for 7 min to a solution containing benzoic hydrazide (0.136 mg) and hydrogen peroxide (50 μl). Finally, slides were mounted with ProlongGold. Both panels have been previously validated^25,69,70^.

### Image acquisition and analysis using quantitative immunofluorescence (QIF)

The entire tumor and surrounding stromal areas were scanned using a Vectra Polaris multispectral imaging station (Akoya Biosciences). Depending on the biopsy size, each patient’s sample was represented between 4 and 409 independent ×20 magnification fields of view (FOVs). These images were later analyzed by quantitative measurement of the fluorescent signal using the AQUA® QIF platform (Navigate BioPharma) that enables objective and sensitive measurement of targets within user-defined tissue compartments. The QIF score of each marker was calculated by dividing the marker pixel intensity by the area of the corresponding compartment defined by the cytokeratin positivity (e.g., tumor compartment), absence of cytokeratin (e.g., stroma compartment) of the DAPI-positive cells (e.g., total tissue including tumor and stroma compartments). Scores were normalized to the exposure time at which the images were captured, allowing scores collected at different exposure times to be comparable. Finally, all the stained slides were examined, and cases with staining artifacts were excluded. This data was used to measure the levels of all markers in LFS vs non-LFS and to compare DCIS vs. IDC.

### Image analysis using single-cell phenotyping data

The images obtained from the Vectra Polaris scan were analyzed using InForm (V.2.6.0, PerkinElmer), an automated image analysis software. For each case, a tissue/cell segmentation algorithm was created using 5–10 representative FOVs selected by a pathologist. These images were used to train the software to define background, tumor and stromal compartments based on the marker staining of these areas. Then, cell segmentation parameters were selected to identify and segment individual cells based on nuclear DAPI staining. The cell segmentation performance was controlled by direct visualization of samples by a trained observer. Next, the software was trained to recognize cell subtypes based on marker expression (CD8 for effector T cells, CD4 for helper T cells, CD20 for B-lymphocytes, cytokeratin for tumor epithelial cells and absence of these markers for stromal cells). Once the algorithm was created, it was applied to the rest of the images of each individual case. Finally, the images were reviewed using visual inspection to ascertain reproducibility and modify or exclude those with suboptimal phenotyping. Once all the images were analyzed, the information was processed using Akoya’s R package PhenoReports (https://akoyabio.github.io/phenoptrReports/index.html). This tool merges the data from all the individual field of view images of a particular case and creates a data file containing information related to individual cell events, density in each tissue compartment (number of positive cells per mm2), marker-based phenotype and spatial location within the specimen (X and Y coordinates).

### Statistical analysis

Genomics statistical analyses were completed in R. Comparison of continuous variables were completed using a two-tailed Student’s *t* test. Comparison of alteration rates was done using Fisher’s exact or chi-squared tests. For the mIF analyses, statistical comparisons were conducted with the Mann–Whitney *U*-tests. Linear and rank correlations were conducted with the Pearson and Spearman correlations.

### Reporting summary

Further information on research design is available in the Nature Portfolio Reporting Summary linked to this article.

## Supplementary information

Supplementary Information

Description of Additional Supplementary Files

Supplementary Data 1–22

Reporting Summary

Transparent Peer Review File

## Source data

Source Data

## Data Availability

The individual-level clinical data are available under restricted access due to IRB constraints, access can be obtained by contacting the authors. The data generated from our analyses are included in the manuscript main text, tables, and figures and online Supplementary Materials (available online). The raw RNA sequencing data is deposited in the Gene Expression Omnibus (GEO) repository under accession number GSE306117. Raw tumor genomic sequencing data is deposited in the dbGaP database entitled LFS Genomics under accession number phs003348. We also analyzed data from the TCGA through dbGaP Project #21931, from dataset phs000178.v10.p8. Source data are provided with this paper.

## Code availability

All computer code used in this paper has been previously reported and is available at the listed references.
