# Supplementary material for: Distinct genomic and immunologic tumor evolution in germline TP53-driven breast cancers
Source: Nat Commun. 2026 May 21;17:7300. doi: 10.1038/s41467-026-73163-4 (PMC13402668; doi:10.1038/s41467-026-73163-4)
Supplement: Supplementary file 1 — Supplementary Information [file 41467_2026_73163_MOESM1_ESM.pdf]

a.

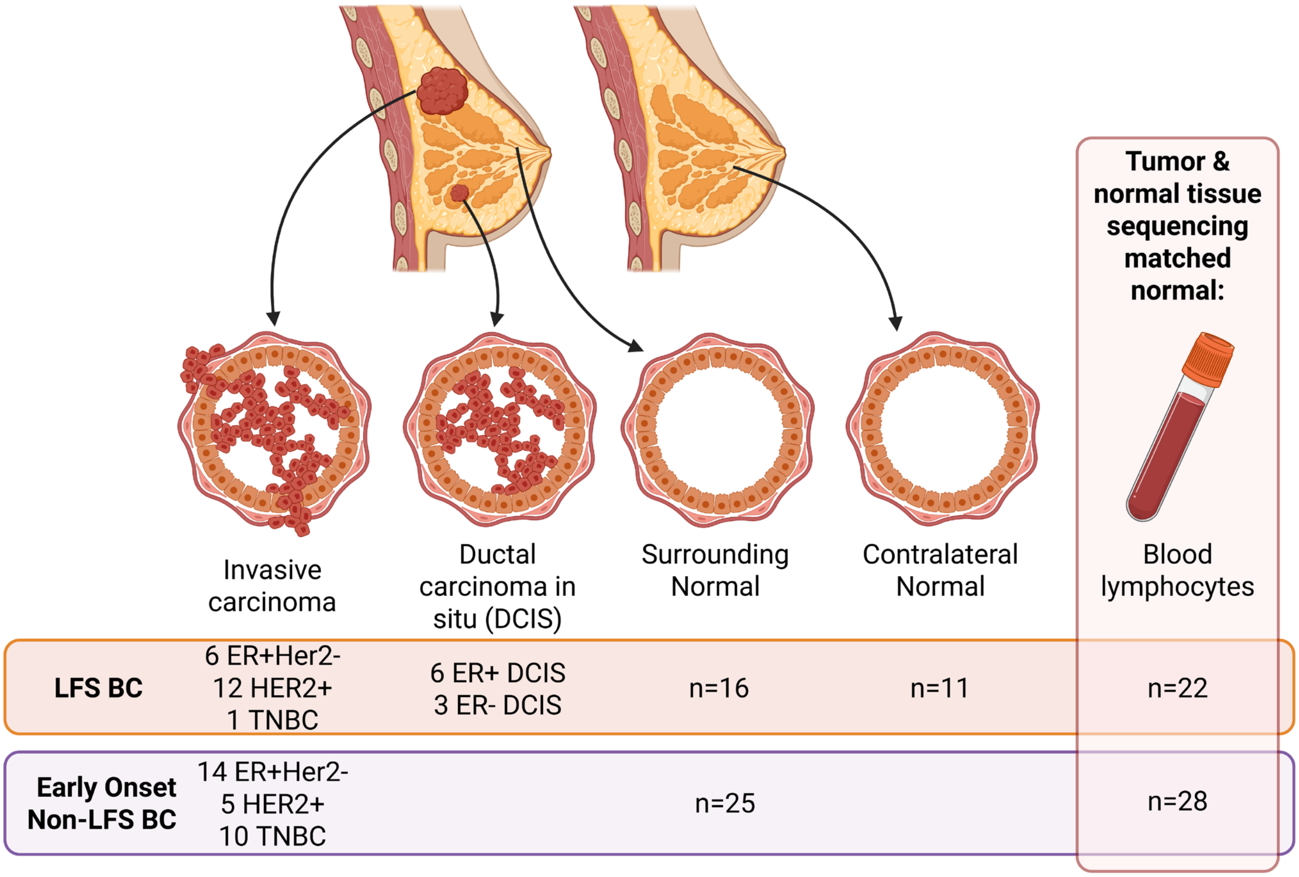

b.

	LFS-BC		LFS-CNBT		LFS-SNBT		NonLFS-BC		NonLFS-SNBT	
	Patients	Tissue	Patients	Tissue	Patients	Tissue	Patients	Tissue	Patients	Tissue
Total	22	28	11	11	13	16	28	29	24	25
Targeted Sequencing	17	21	11	11	12	15	26	27	-	-
RNASeq	15	16	5	5	6	7	24	25	24	25
WGS	16	19	-	-	-	-	-	-	-	-
p53 IHC	16	20	-	-	-	-	-	-	-	-
mIF	20	25	-	-	-	-	27	28	-	-

Supplementary Fig.1: Tissues analyzed in this study from Penn Medicine. a. Breast tumors including both invasive ductal carcinoma (IDC) and ductal carcinoma in situ (DCIS), surrounding normal breast tissue, contralateral normal breast tissue, and blood specimens were obtained with informed consent via a Penn/CHOP IRB approved protocol (LFS/TP53 Biobank) for 28 breast cancers from 22 patients with Li Fraumeni Syndrome (LFS) or the Penn Medicine Biobank (PMBB) for 29 breast cancers from 28 patients from early-onset non-LFS patients. This figure was created in BioRender **b.** Breakdown of number of tissues from number of patients that were subjected to targeted sequencing, transcriptomic (RNASeq), whole genome sequencing (WGS), and Single stain p53 immunohistochemistry (IHC) and Multiplex immunofluorescence (mIF) analyses. BC, breast cancer; LFS, Li Fraumnei Syndrome; CNBT, Contralateral Normal Breast Tissue; SNBT, Surrounding Normal Breast Tissue; ER: Estrogen Receptor; HER2: receptor tyrosine-protein kinase erbB-2; TNBC: triple negative breast cancer. Source data are provided as a Source Data file.

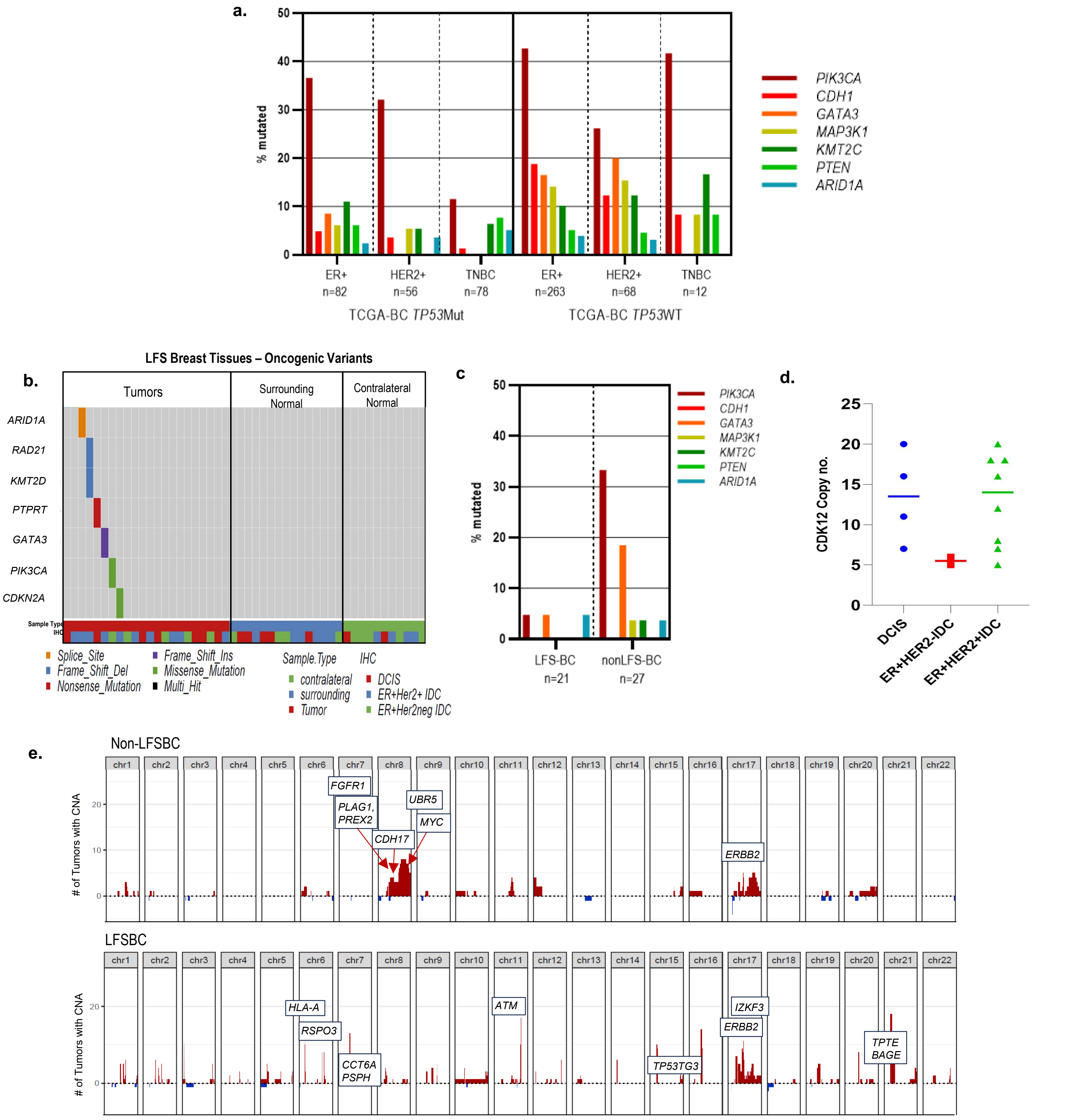

Supplementary Fig.2: Rates of common breast cancer oncogenic alterations stratified by p53 status. **a.** Fraction of tumors from TCGA-BC cohort with oncogenic mutations in the indicated genes. Tumors are stratified by somatic *TP53* mutation status. **B.** Common breast cancer oncogenic alterations identified by Mutect in LFS-BC and normal tissues. **c.** Fraction of LFS-BC and early onset nonLFS-BC with oncogenic mutations in the indicated genes. **d.** Amplification of *CDK12* in LFS-BC. Copy number state for *CDK12* was normalized to the copy number state of chr17p and shown as dot plot with line at median. **e.** Chromosomal plot of amplifications in nonLFS-BC and LFS-BC. Callouts represent oncogenes from Cancer Gene Census. Del, deletion; DCIS, ductal carcinoma in situ; ER, estrogen receptor; HER2, erbb2 receptor; IDC, invasive ductal carcinoma; LFS-BC, Li Fraumeni Syndrome breast cancer; Ins, insertion; TNBC, triple negative breast cancer; *TP53*Mut, positive for somatic *TP53* mutation; *TP53*WT, negative for somatic *TP53* mutation. Source data are provided as a Source Data file.

a.

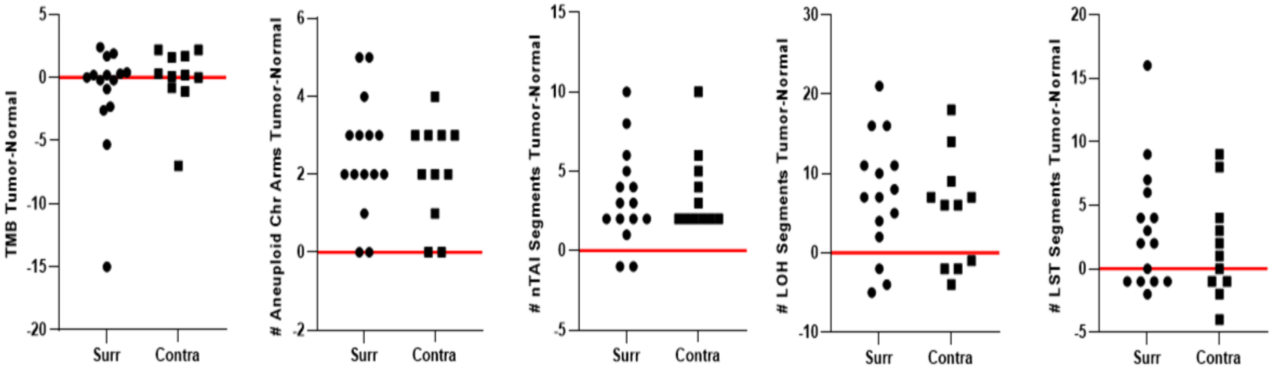

b.

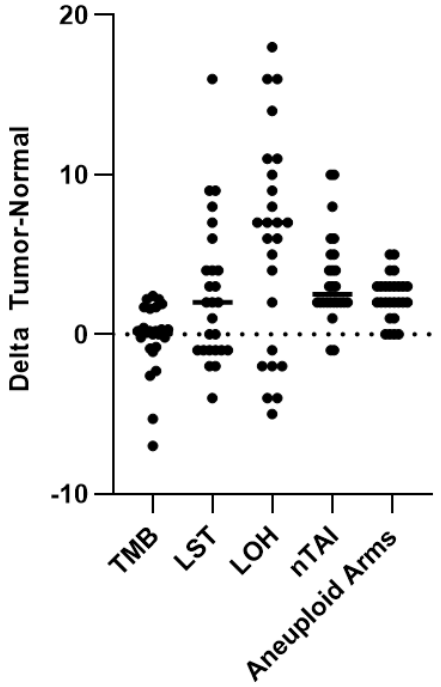

Tumor - Normal Delta (26 comparisons)	# tumor enriched (delta>1)	frac tumor enriched
Mutations	7	27%
LOH segments	19	73%
LST segments	17	65%
nTAI segments	24	92%
Aneuploid chr arms	26	100%

Supplementary Fig.3: Genomic instability in pairs of LFS-BC versus LFS normal breast tissues. **a.** Ratio of TMB, number of aneuploid (Chr) arms, number of nTAI segments, number of genomic LOH segments and number of LST segments in individual LFS-BC sample versus its own matched surrounding (Surr, circles) or contralateral (Contra, squares) normal breast tissue sample. Ratios above the red line (0) indicate the genomic instability measure was higher in the tumor than the normal breast tissue. Genomic instability measures were derived from matched analyses of tumor versus the patient's blood data and normal breast tissue data versus the patient's blood data. Each data point represents one tumor-normal pair. **b.** Delta of the TMB, LST, LOH, nTAI, aneuploid arms measures comparing individual LFS-BC sample versus its own normal (26 comparisons) and fraction of tumors with enrichment of the genomic instability measure. Chr, chromosome; LFS-BC, Li Fraumeni Syndrome breast cancer; LOH, loss of heterozygosity; LST, large state transitions; nTAI, non-telomeric allelic imbalance; TMB, tumor mutation burden. Source data are provided as a Source Data file.

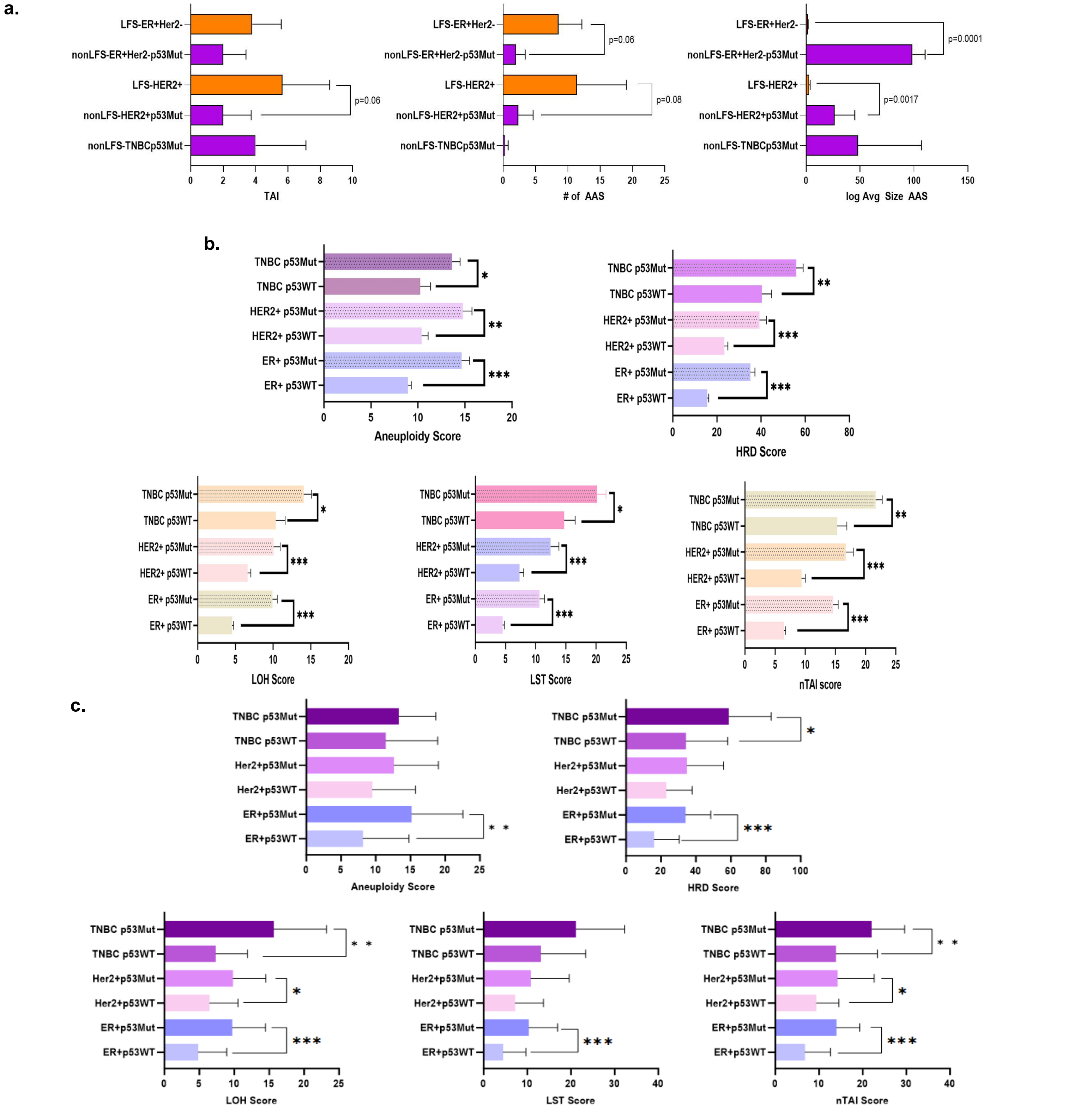

Supplementary Fig.4: Chromosomal instability stratified by *TP53* mutation status. a. Telomeric allelic imbalance, amplified aneuploid segments and average size of AAS in LFS_BC versus nonLFS-BC with acquired p53 mutations stratified by hormone receptor subtype. **b.** Average aneuploidy score, average HRD score, and average number of segments of genomic LOH, LST, and nTAI in ER+Her2-, HER2+ and TNBC from TCGA with or without a somatic *TP53* mutation. **c.** Average aneuploidy score, average HRD score, and average number of segments of genomic LOH, LST, and nTAI in ER+Her2-, HER2+ and TNBC from premenopausal women in TCGA with or without a somatic *TP53* mutation. All data represented as Mean ± SEM. AAS: amplified aneuploid segments; ER: Estrogen Receptor; HER2: receptor tyrosine-protein kinase erbB-2; HRD, homologous recombination score; LOH, loss of heterozygosity; LST, large state transitions; TAI, non-telomeric allelic imbalance; TNBC: triple negative breast cancer. Source data are provided as a Source Data file. Statistical comparisons between p53WT and p53mut of the indicated tumor types were performed with a two-tailed unpaired Student's t-test, *$p<0.05$; **$p<0.01$; *** $p<0.001$; ****$p<0.0001$.

a.

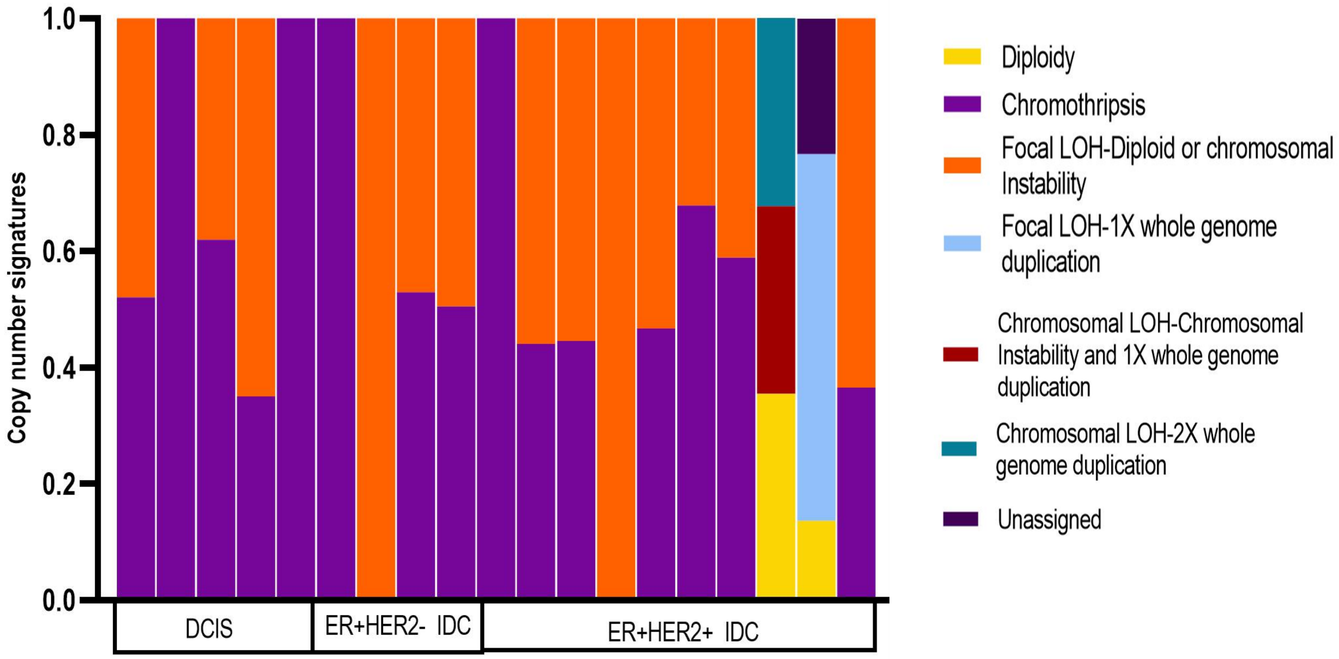

b.

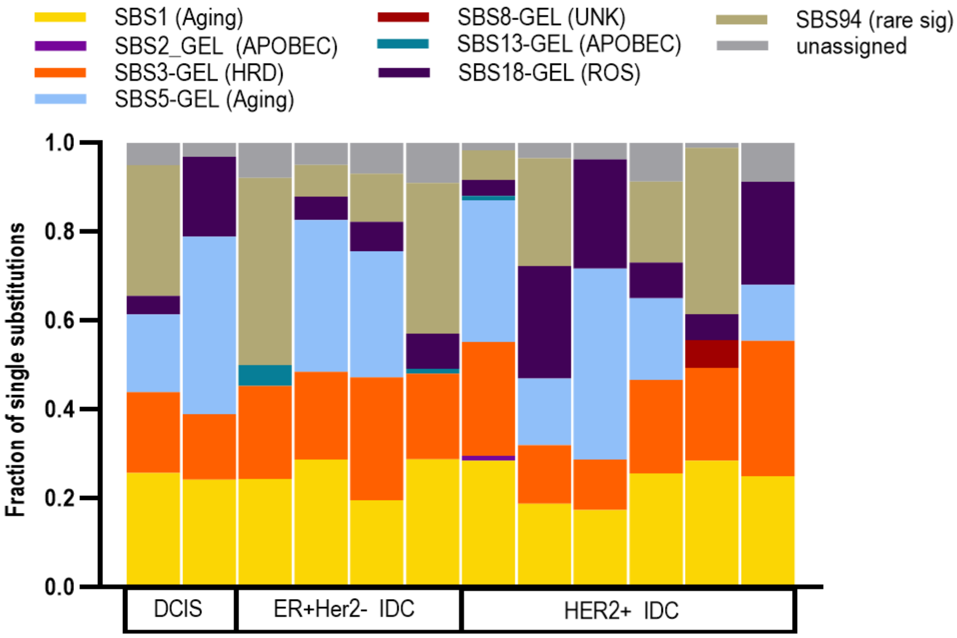

Supplementary Fig.5: Mutational processes in LFS-BC. a.Copy number signature profile of LFS-BC from whole genome sequencing. b. Single base pair mutational signature profiles derived from FitMS of LFS-BC by whole genome sequencing. LOH, loss of heterozygosity; HRD, homologous recombination deficiency; ROS, reactive oxygen species/ SBS, single base substitution; UNK, unknown. Source data are provided as a Source Data file.

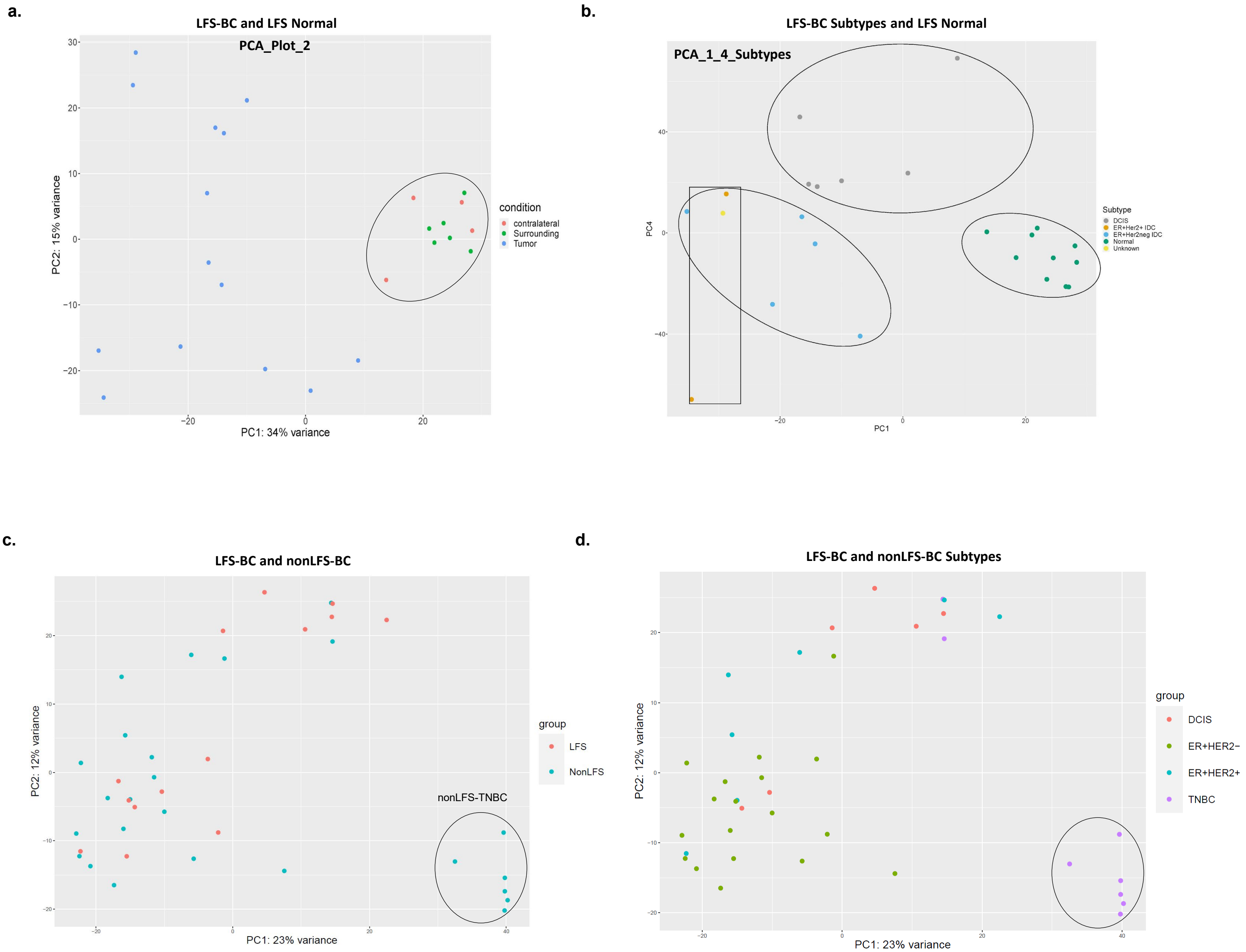

Supplementary Fig.6: PC plots showing expression data subgroupings. **a.** PC plot of RNAseq data from LFS-BC and LFS normal breast tissue showing clustering of tumors separate from both contralateral and surrounding normal breast tissue. **b.** PC plot of RNAseq data from LFS-BC stratified by subtype (DCIS, ER+Her2- and HER2+ IDC) and LFS normal breast tissue showing clustering of DCIS separate from invasive tumors and separate from normal breast tissue. **c.** PC plot of RNAseq data from LFS-BC and non-LFSBC showing intermixing of most tumors except one grouping (circle). **d.** PC plot of RNAseq data from LFS-BC and nonLFS-BC stratified by subtype (DCIS, ER+Her2-, HER2+ IDC and TNBC) showing clustering of TNBC tumors separate from invasive tumors and separate from normal breast tissue. BC, breast cancer; DCIS, ductal carcinoma *in situ*; ER: Estrogen Receptor; HER2: receptor tyrosine-protein kinase erbB-2; IDC, invasive ductal carcinoma; LFS, Li Fraumeni Syndrome; TNBC: triple negative breast cancer. Source data are provided as a Source Data file.

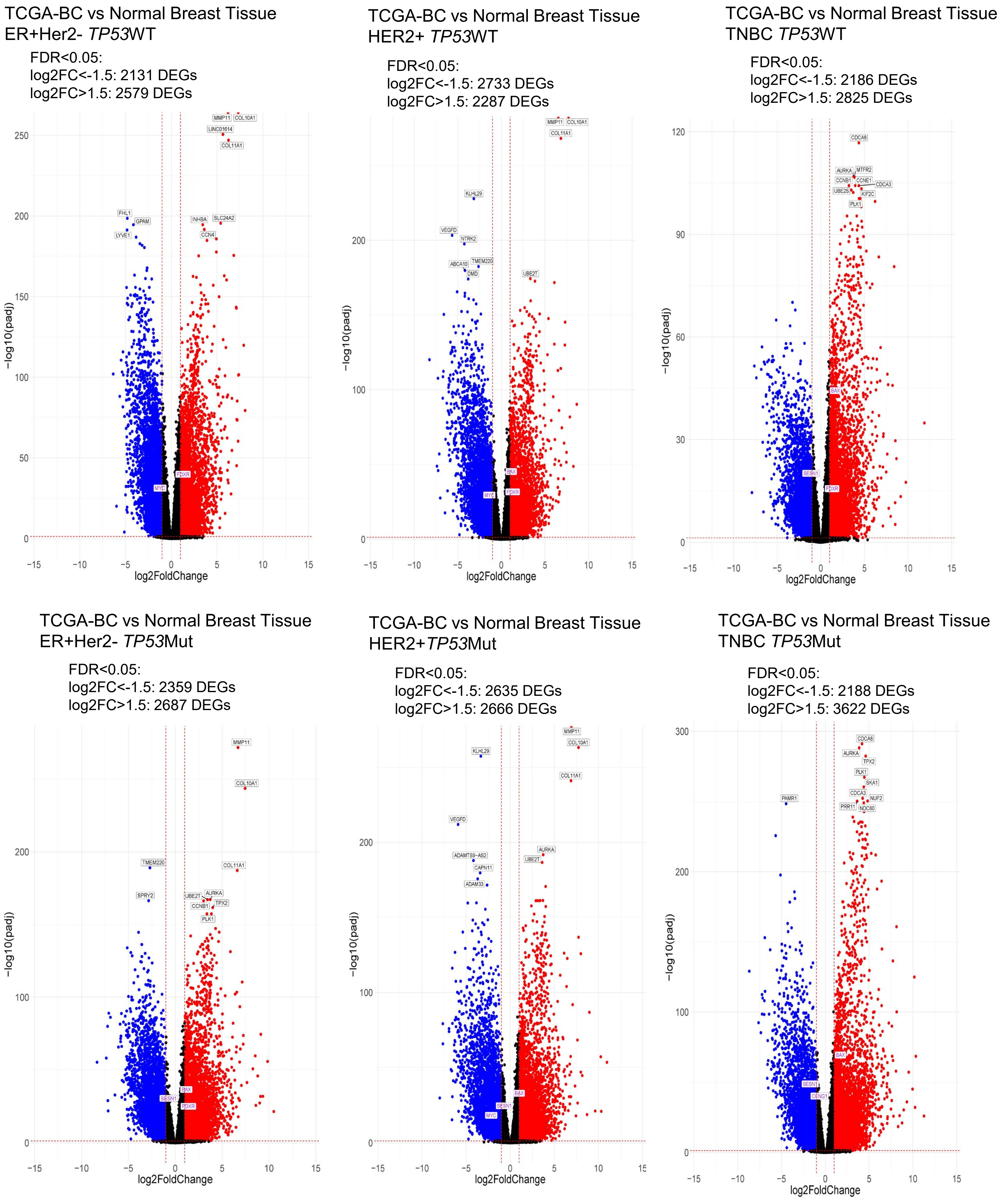

Supplementary Fig.7: Differential gene expression analysis in TCGA-BC cohort stratified by somatic *TP53* mutation status and hormone receptor subtype. ER: Estrogen Receptor; HER2: receptor tyrosine-protein kinase erbB-2; TNBC: triple negative breast cancer (Blue: Down, Red: Up and Black: No change). Source data are provided as a Source Data file.

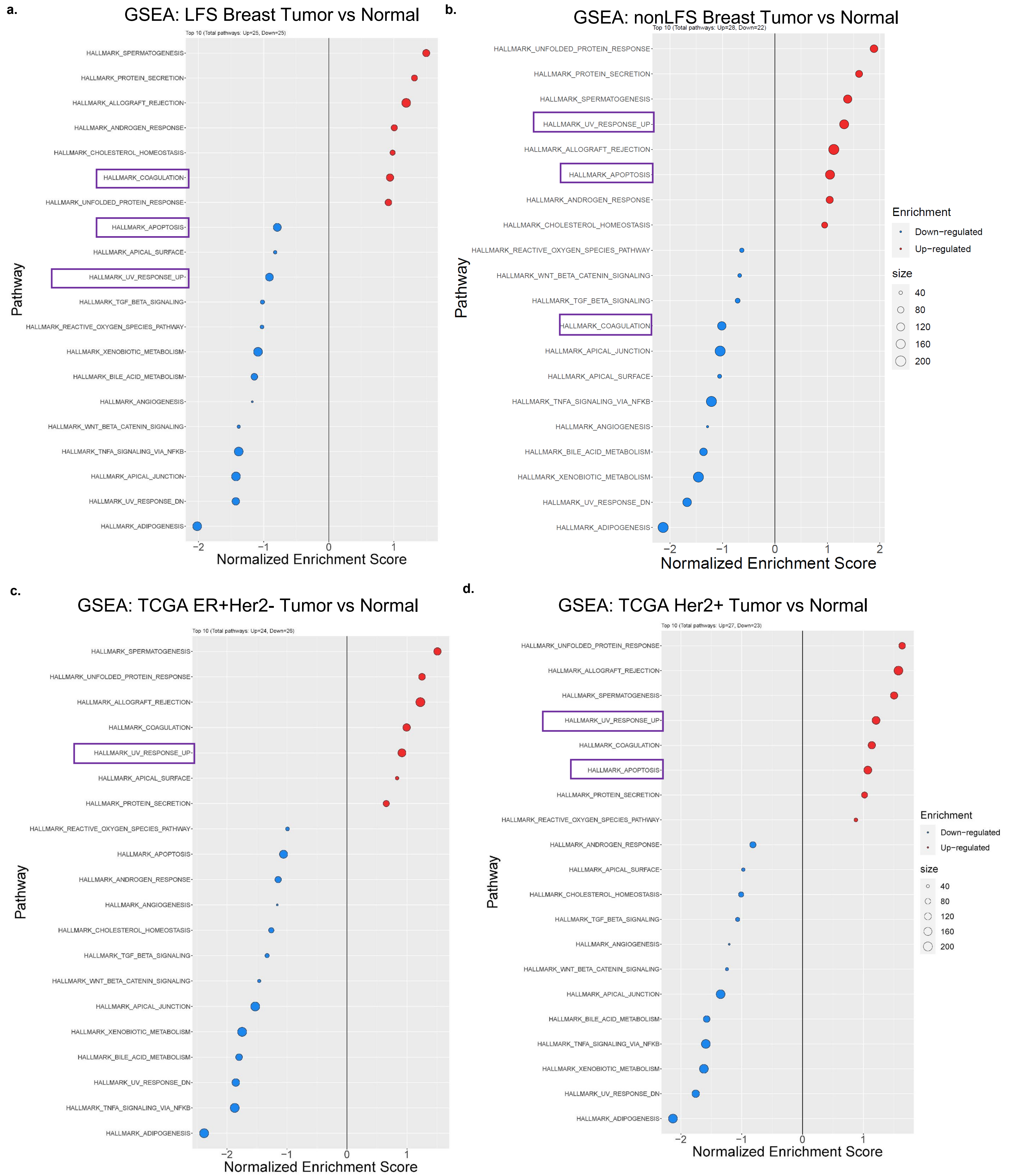

Supplementary Fig.8: Gene set enrichment analysis (GSEA) of LFS-BC and non-LFS BC. **a.** GSEA of LFS-BC compared to LFS normal breast tissue. **b.** GSEA of nonLFS-BC (ER+) compared to nonLFS normal breast tissue. **c.** GSEA ER+Her2- nonLFS-BC compared to normal breast tissue from TCGA. **d.** GSEA Her2+ nonLFS-BC compared to normal breast tissue from TCGA. BC: Breast cancer; ER: Estrogen Receptor; HER2: receptor tyrosine-protein kinase erbB-2; LFS: Li Fraumeni Syndrome. Source data are provided as a Source Data file.

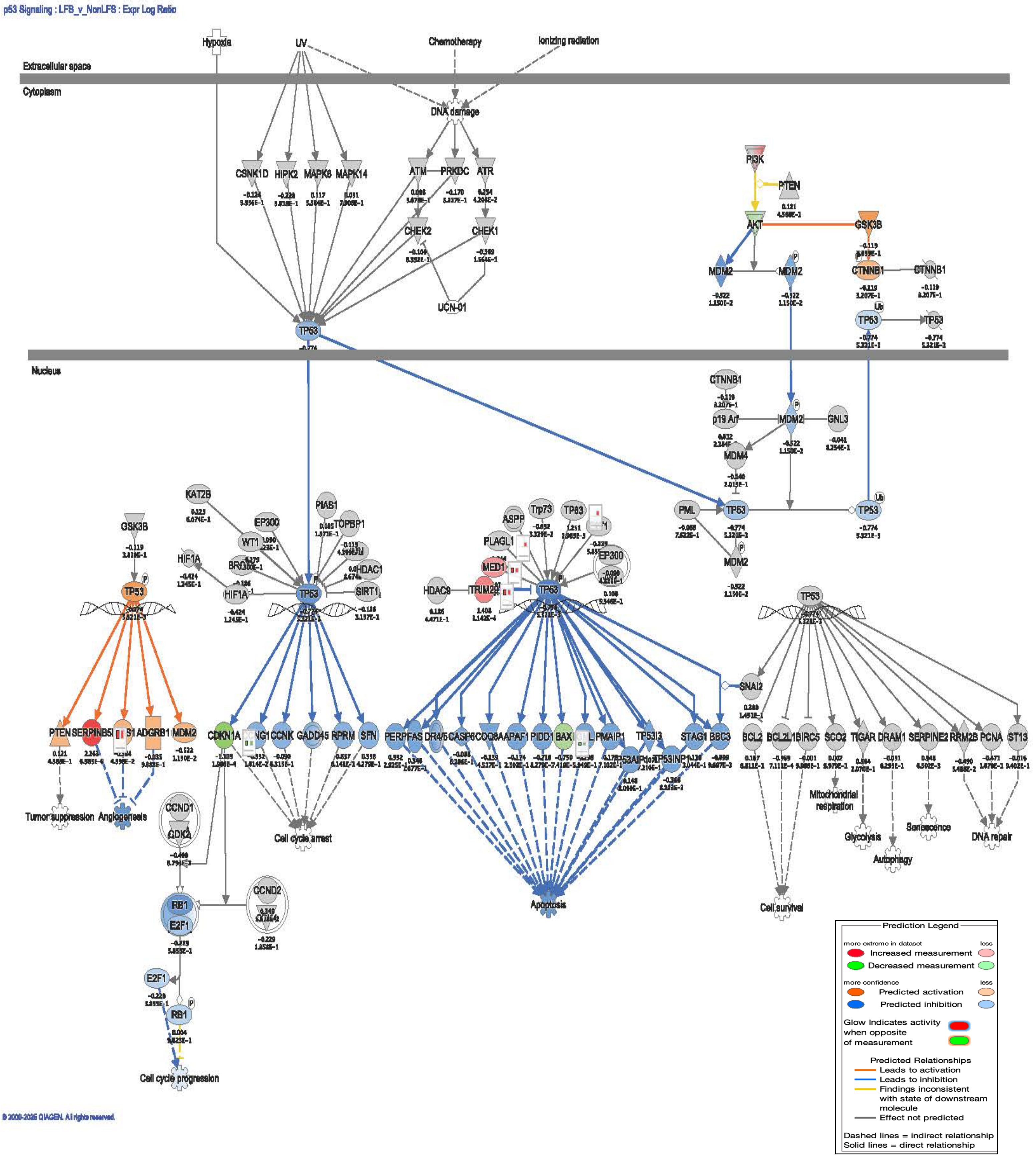

Supplementary Fig.9: Ingenuity Pathway Analysis (IPA) of LFS-BC versus nonLFS-BC tissues. Data were analyzed with the use of QIAGEN IPA (QIAGEN Inc.).Canonical pathways identified by IPA are shown, ranked by activation z-score. Positive z-scores (orange) indicate predicted pathway activation in LFS-BC relative to nonLFS-BC controls, while negative z-scores (green) indicate predicted inhibition. The magnitude of the z-score reflects the strength and direction of pathway regulation, with pathways with |z| ≥ 2 considered significantly activated or inhibited. Source data are provided as a Source Data file.

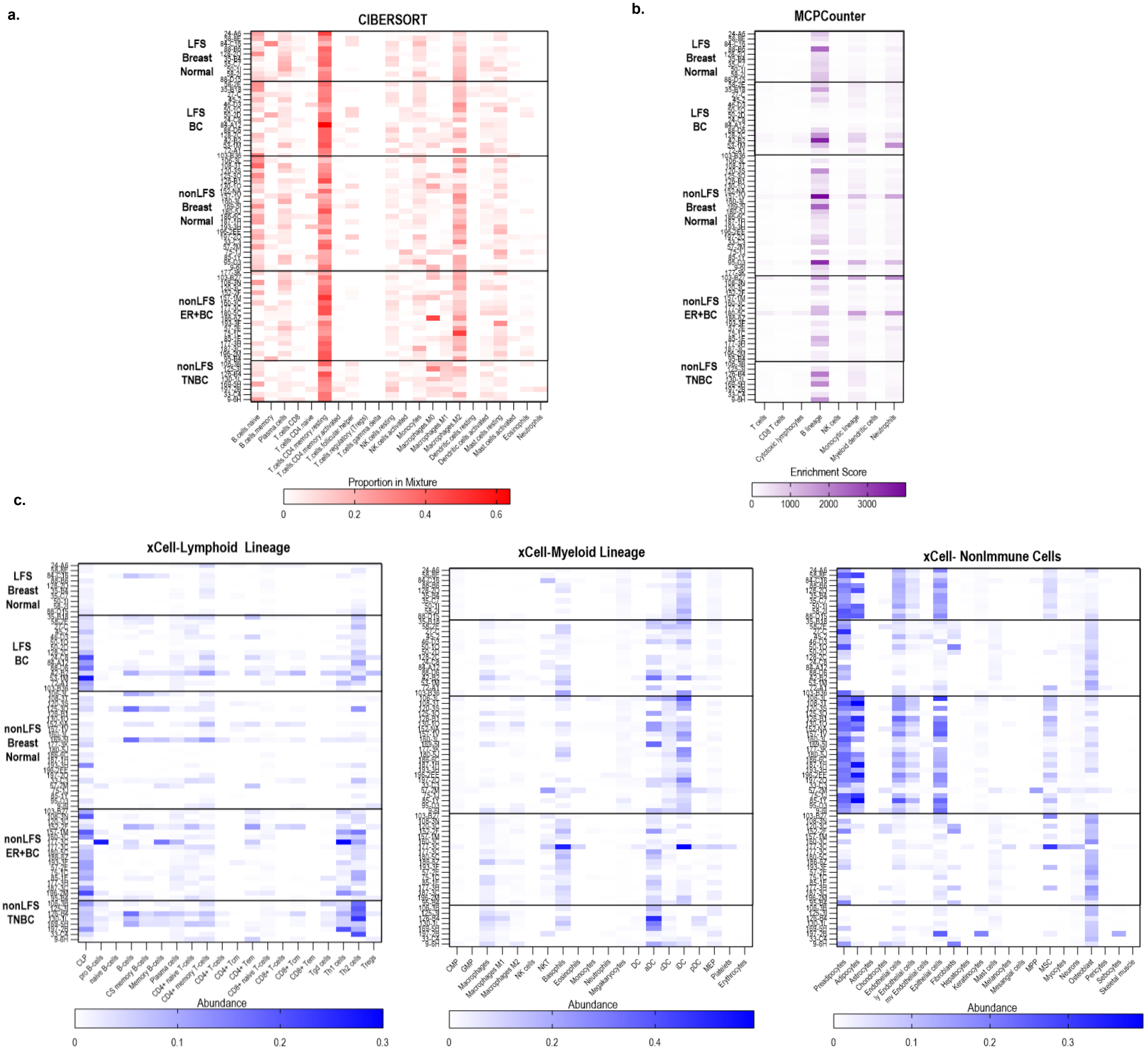

Supplementary Fig.10: Analysis of tumor microenvironment . a**.** CIBERSORT analysis of RNAseq data estimating immune cell proportions in LFS and nonLFS-BC and normal breast tissues. **b.** MCPCounter analysis of RNASeq data estimating enrichments scores of immune cell proportions in LFS and nonLFS-BC and normal breast tissues. **c.** xCell analysis of RNASeq data estimating abundance of lymphoid lineage immune cells, myeloid lineage immune cells and non-immune cells in LFS and nonLFS-BC and normal breast tissues. Breast cancer; ER: Estrogen Receptor; HER2: receptor tyrosine-protein kinase erbB-2; LFS: Li Fraumeni Syndrome; TNBC: Triple negative breast cancer. Source data are provided as a Source Data file.

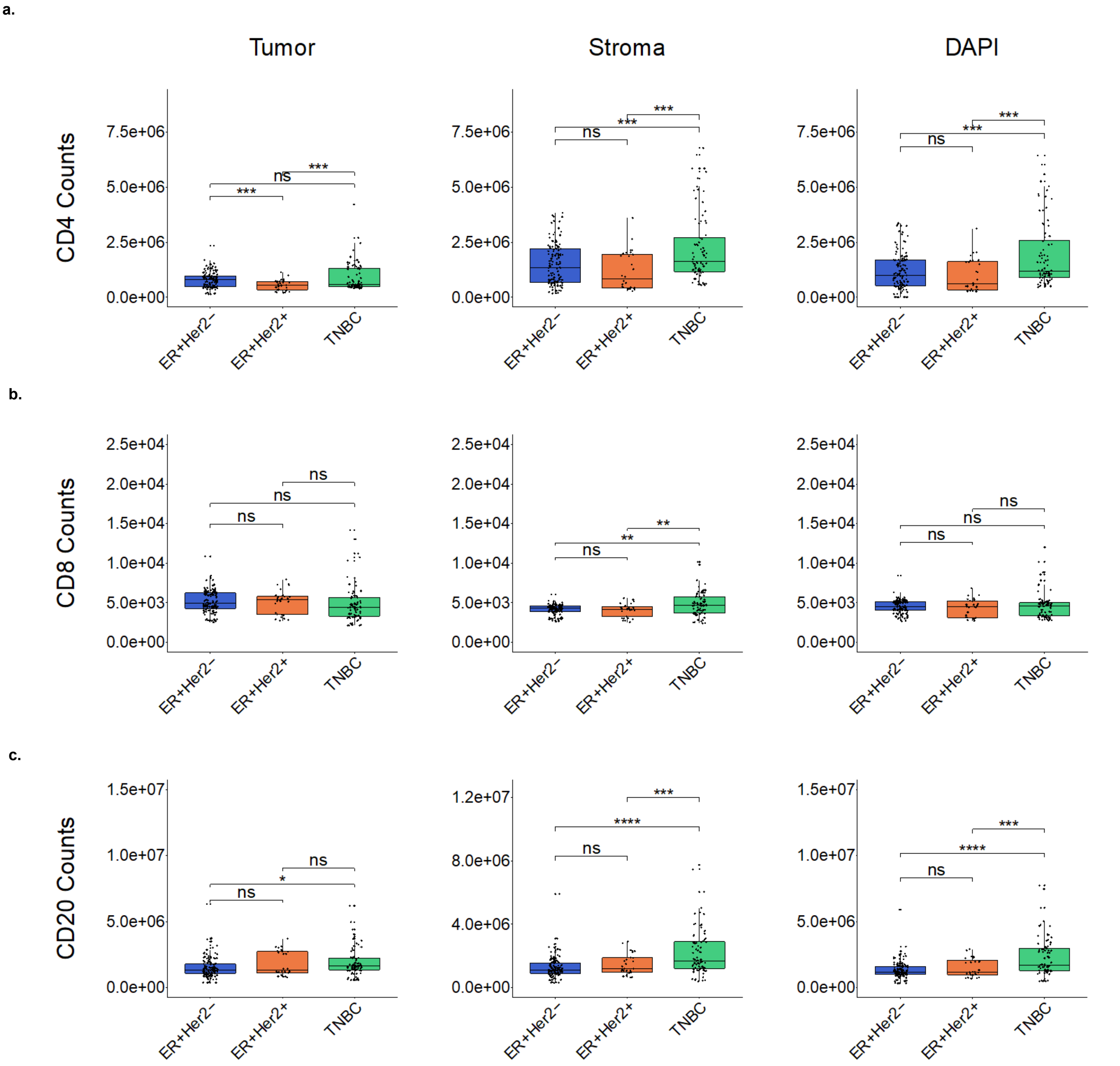

Supplementary Fig.11: Immune infiltration in ER+Her2-BC (n=14), HER2+-BC (n=5) and TNBC (n=10) in premenopausal nonLFS-BC. a. CD4+ cells in tumor, stromal and DAPI channel using multiplex immunohistochemistry. **b.** CD8+ cells in tumor, stromal and DAPI channel using multiplex immunohistochemistry. **c.** CD20+ cells in tumor, stromal and DAPI channel using multiplex immunohistochemistry. ER: Estrogen Receptor; HER2: receptor tyrosine-protein kinase erbB-2; LFS: Li Fraumeni Syndrome; TNBC: Triple negative breast cancer. For all box plots centre line is median, the bounds of the box are the upper and lower quartiles, and the whisker bounds are defined as upper limit=Q3+(1.5xIQR), lower limit=Q1-(1.5xIQR). Statistical comparisons were performed by Welch's t-test, ns, non-significant; *p<0.05; **p<0.01; *** p<0.001; ****p <0.0001. Source data are provided as a Source Data file.

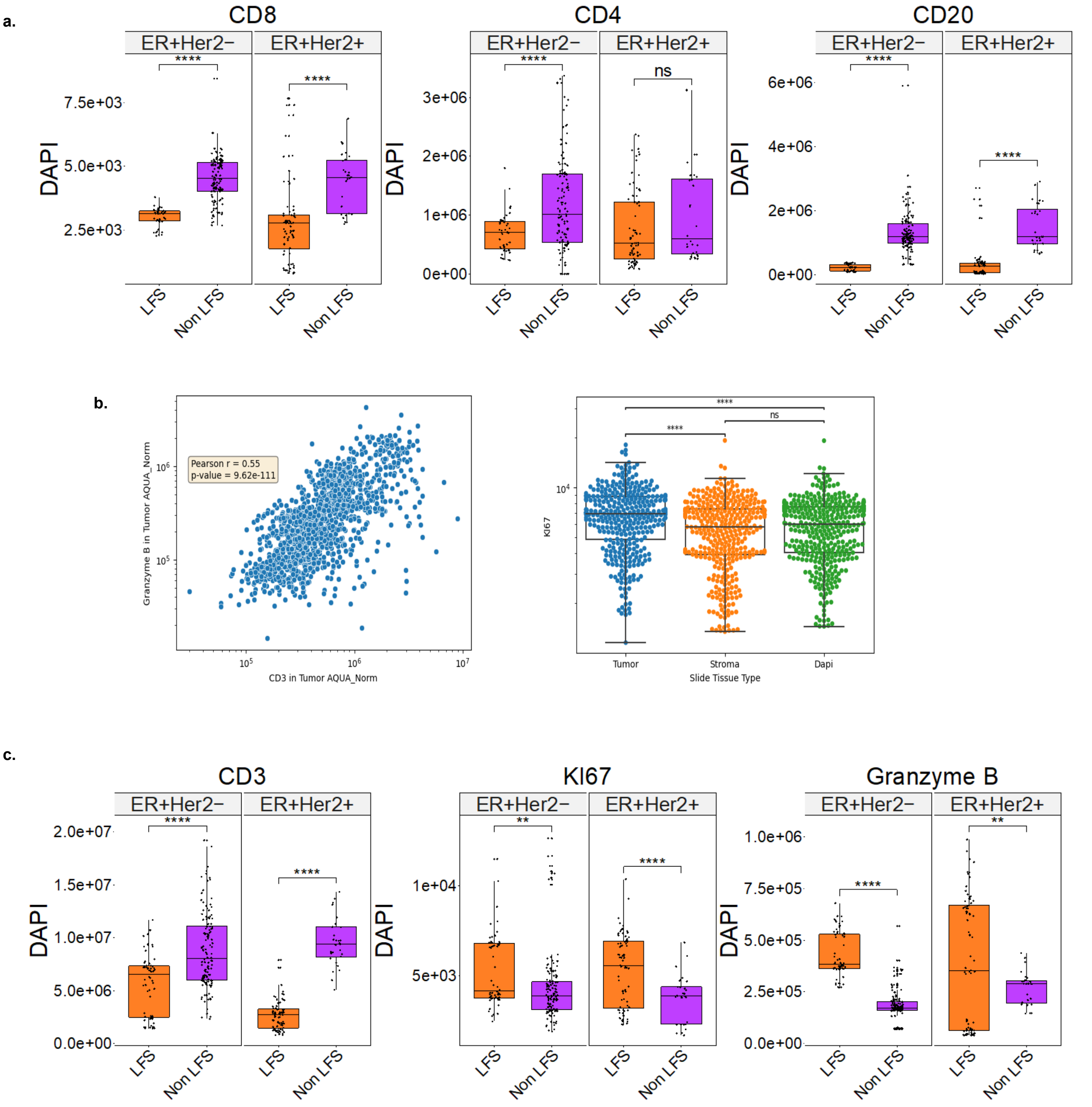

Supplementary Fig.12: Multiplex Immunohistochemistry to study the tumor immune microenvironment in LFS-BC versus nonLFS-BC. a. Levels of CD8+, CD4+ and CD20+ cells in the DAPI compartment in LFS-BC versus nonLFS-BC. **b.** Correlation of Granyzme B and CD3 staining in tumor and demonstration of higher Ki67 staining in tumor versus stroma and DAPI. **c.** T-cell activation panel in LFS-DCIS compared to LFS-IDC. ACT, T-cell activation panel; BC, breast cancer; CK, cytokeratin; ER: Estrogen Receptor; GrzmB, granzyme B; LFS: Li Fraumeni Syndrome; TNBC: Triple negative breast cancer. For all box plots centre line is median, the bounds of the box are the upper and lower quartiles, and the whisker bounds are defined as upper limit=Q3+(1.5xIQR), lower limit=Q1-(1.5xIQR). Dot plots are shown with line at median. Statistical comparisons were performed by Welch's t-test, ns, non-significant; *p<0.05; **p<0.01; *** p<0.001; ****p <0.0001. Source data are provided as a Source Data file.

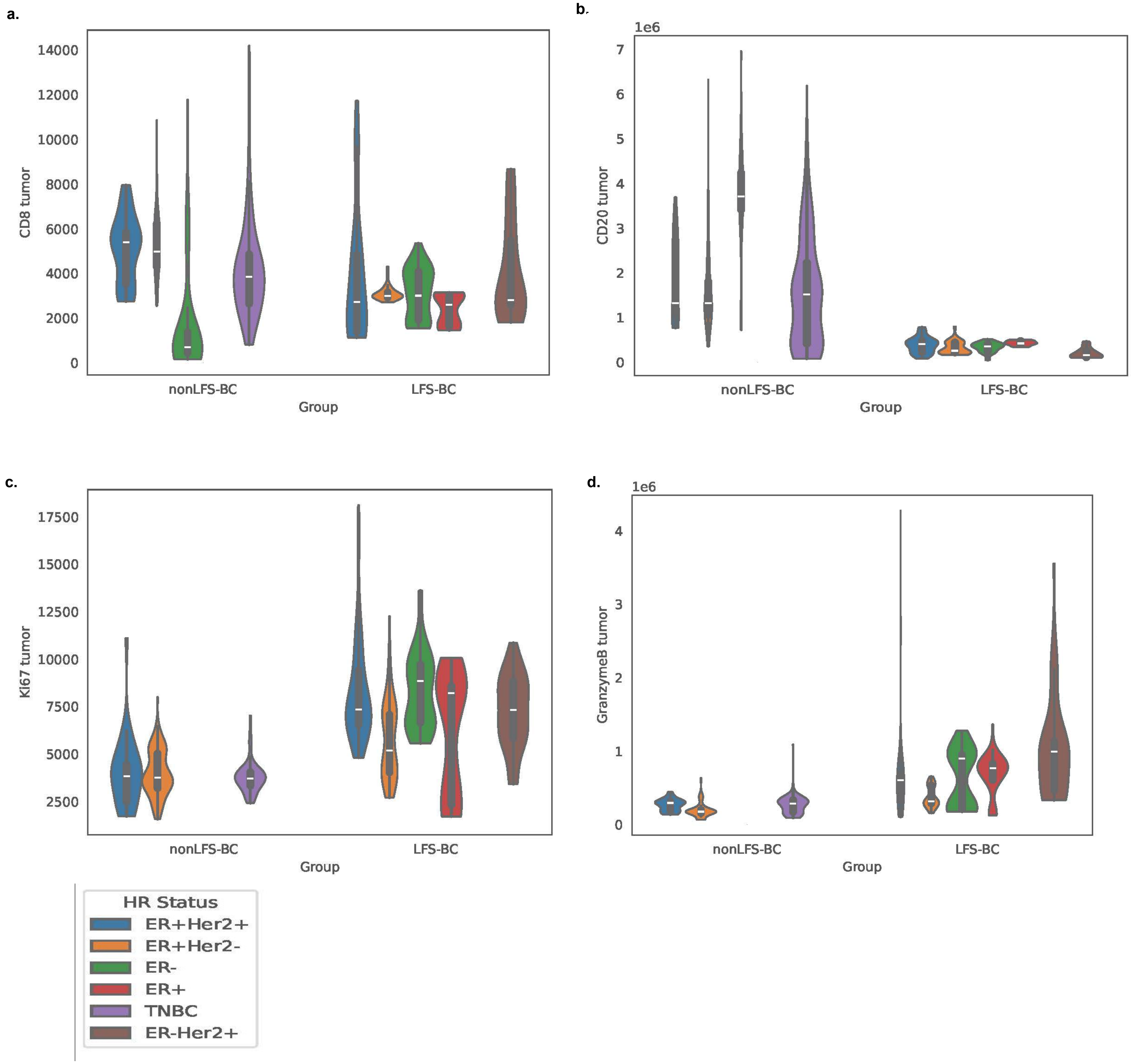

Supplementary Fig.13: Multiplex immunohistochemistry analysis in expanded cohort of LFS-BC versus nonLFS-BC. a. Tumor infiltration of CD8+ cells in HER2+ BC, ER+Her2- BC, ER+ DCIS, ER- DCIS, and TNBC in nonLFS-BC and LFS-BC from both pre-menopausal and post-menopausal patients. **b.** Tumor infiltration of CD20+ cells in HER2+ BC, ER+Her2- BC, ER+ DCIS, ER- DCIS, and TNBC in nonLFS-BC and LFS-BC from both pre-menopausal and post-menopausal patients. **c.** Tumor infiltration of Ki67+ cells in HER2+ BC, ER+Her2- BC, ER+ DCIS, ER- DCIS, and TNBC in nonLFS-BC and LFS-BC from both pre-menopausal and post-menopausal patients. **d.** Tumor infiltration of GranzymeB+ cells in HER2+ BC, ER+Her2- BC, ER+ DCIS, ER- DCIS, and TNBC in nonLFS-BC and LFS-BC from both pre-menopausal and post-menopausal patients. DCIS, ductal carcinoma in situ; ER: Estrogen Receptor; HER2: receptor tyrosine-protein kinase erbB-2; LFS: Li Fraumeni Syndrome; TNBC: Triple negative breast cancer. Violin plots display the distribution of the data, with density estimated only within the observed data range. The white spot indicates the median. Source data are provided as a Source Data file.

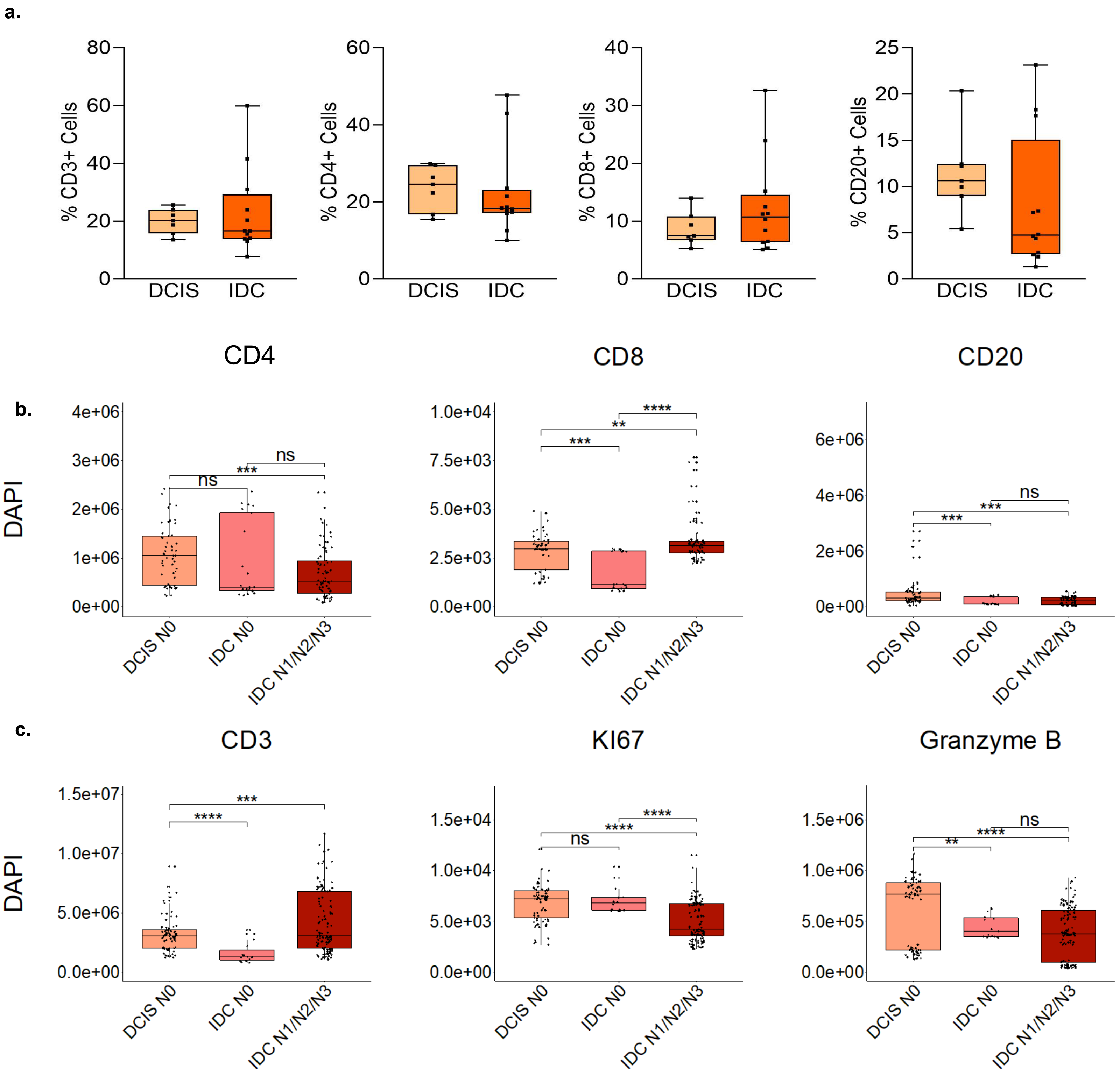

Supplementary Fig.14: Immune staining in LFS-BC stratified by DCIS and IDC. a. Single immune staining in LFS DCIS versus LFS IDC for CD3+ T cells, CD4+ T cell, CD8+ T cells and CD20+ T cells. **b.** Levels of CD8+, CD4+ and CD20+ cells in the DAPI compartment in LFS-DCIS vs LFS-IDC. **c.** Levels of CD3+, granzymeB and Ki67+ cells in the DAPI compartment in LFS-DCIS vs LFS-IDC. DCIS, ductal carcinoma in situ; IDC, invasive ductal carcinoma; LFS, Li Fraumeni Syndrome. For all box plots centre line is median, the bounds of the box are the upper and lower quartiles, and the whisker bounds are defined as upper limit=Q3+(1.5xIQR), lower limit=Q1-(1.5xIQR). Dot plots are shown with line at median. Statistical comparisons were performed by Welch's t-test, ns, non-significant; *p<0.05; **p<0.01; *** p<0.001; ****p <0.0001. Source data are provided as a Source Data file.

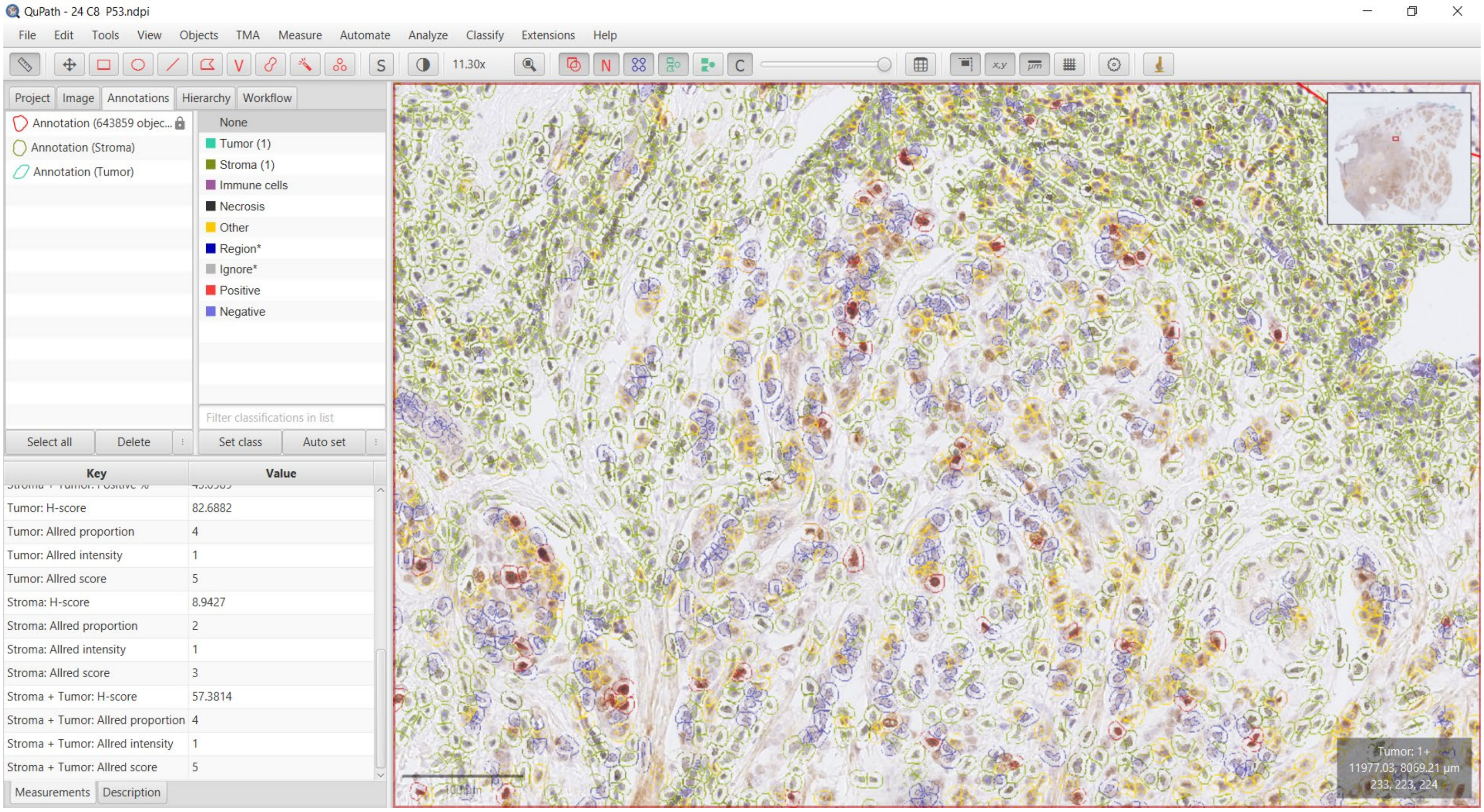

Supplementary Fig.15. Quantification of p53 Immunohistochemistry using QuPath. Example image of p53 immunohistochemistry quantification methodology in QuPath v0.5.0 for tumor 24-C8. In areas marked as tumor and/or normal, a cell detection command was used to identify cells, annotated by different cell types (tumor, stroma, immune, necrosis, and other). We then trained a cell classifier based on these annotations, saved the classifier, and applied it to the cells in the image. Source data are provided as a Source Data file.
