# Supplementary material for: Distinct genomic and immunologic tumor evolution in germline TP53-driven breast cancers
Source: Nat Commun. 2026 May 21;17:7300. doi: 10.1038/s41467-026-73163-4 (PMC13402668; doi:10.1038/s41467-026-73163-4)
Supplement: Supplementary file 2 — Description of Additional Supplementary Files [file 41467_2026_73163_MOESM2_ESM.pdf]

<u>Description of Additional Supplementary Files</u>

Supplementary Data 1: Clinical Data for Sequenced LFS-BC, LFS breast tissues, and non-LFS BC from Penn Medicine

Supplementary Data 2: Genomic data from LFS-BC, LFS breast tissues, and nonLFS-BC from Penn Medicine

Supplementary Data 3: Genomic data from TCGA tumors with germline TP53 variants

Supplementary Data 4: Genes for which germline mutation carriers were excluded from TCGA-BC and PMBB nonLFS-BC analyses

Supplementary Data 5: TCGA Breast cancers used in analysis

Supplementary Data 6: Differential expression gene analysis of LFS BC-versus LFS normal breast tissue

Supplementary Data 7: Differential expression gene analysis of ER+Her2- and HER2+ nonLFS-BC vs normal breast tissue

Supplementary Data 8: Differential expression gene analysis of TCGA ER+Her2-/*TP53*WT-BC vs normal breast tissue

Supplementary Data 9: Differential expression gene analysis of TCGA ER+Her2-/*TP53*mut-BC vs normal breast tissue

Supplementary Data 10: Differential expression gene analysis of TCGA HER2+/*TP53*WT-BC vs normal breast tissue

Supplementary Data 11: Differential expression gene analysis of TCGA HER2+/*TP53*mut-BC vs normal breast tissue

Supplementary Data 12: Differential expression gene analysis of TCGA TNBC/*TP53*WT-BC vs normal breast tissue

Supplementary Data 13: Differential expression gene analysis of TCGA TNBC/*TP53*mut-BC vs normal breast tissue

Supplementary Data 14: Differential expression gene analysis of LFS-BC vs nonLFS-BC (ER+Her2- and HER2+ only)

Supplementary Data 15: Hallmark Gene Set Enrichment Analysis (GSEA) of LFS-BC vs nonLFS-BC (ER+Her2- and HER2+ only)

Supplementary Data 16: Hallmark Gene Set Enrichment Analysis (GSEA) of LFS-BC, nonLFS-BC (ER+Her2- and HER2+ only), TCGA BC cohorts versus normal breast tissue

Supplementary Data 17: CIBERSORT analysis of tumor microenvironment in LFS-BC, nonLFS-BC and corresponding normal breast tissue

Supplementary Data 18: MCPCounter analysis of tumor microenvironment in LFS-BC, nonLFS-BC and corresponding normal breast tissue

Supplementary Data 19: xCell analysis of tumor microenvironment in LFS-BC, nonLFS-BC and corresponding normal breast tissue

Supplementary Data 20: Clinical characteristics of discovery versus validation cohort for LFS-BC and nonLFS-BC

Supplementary Data 21: Differential expression gene analysis of LFS-IDC versus LFS-DCIS
Supplementary Data 22: Hallmark Gene Set Enrichment Analysis (GSEA) of LFS-DCIS vs LFS-IDC
