# Supplementary material for: Distinct genomic and immunologic tumor evolution in germline TP53-driven breast cancers
Source: Nat Commun. 2026 May 21;17:7300. doi: 10.1038/s41467-026-73163-4 (PMC13402668; doi:10.1038/s41467-026-73163-4)
Supplement: Supplementary file 4 — Reporting Summary [file 41467_2026_73163_MOESM4_ESM.pdf]

nature portfolio

Corresponding author(s): Kara N. Maxwell

Last updated by author(s): Apr 24, 2024

Reporting Summary

Nature Portfolio wishes to improve the reproducibility of the work that we publish. This form provides structure for consistency and transparency in reporting. For further information on Nature Portfolio policies, see our Editorial Policies and the Editorial Policy Checklist.

Statistics

For all statistical analyses, confirm that the following items are present in the figure legend, table legend, main text, or Methods section.

n/a	Confirmed	
☐	☒	The exact sample size (*n*) for each experimental group/condition, given as a discrete number and unit of measurement
☐	☒	A statement on whether measurements were taken from distinct samples or whether the same sample was measured repeatedly
☐	☒	The statistical test(s) used AND whether they are one- or two-sided<br>*Only common tests should be described solely by name; describe more complex techniques in the Methods section.*
☒	☐	A description of all covariates tested
☐	☒	A description of any assumptions or corrections, such as tests of normality and adjustment for multiple comparisons
☐	☒	A full description of the statistical parameters including central tendency (e.g. means) or other basic estimates (e.g. regression coefficient) AND variation (e.g. standard deviation) or associated estimates of uncertainty (e.g. confidence intervals)
☐	☒	For null hypothesis testing, the test statistic (e.g. *F*, *t*, *r*) with confidence intervals, effect sizes, degrees of freedom and *P* value noted<br>*Give P values as exact values whenever suitable.*
☒	☐	For Bayesian analysis, information on the choice of priors and Markov chain Monte Carlo settings
☐	☒	For hierarchical and complex designs, identification of the appropriate level for tests and full reporting of outcomes
☐	☒	Estimates of effect sizes (e.g. Cohen's *d*, Pearson's *r*), indicating how they were calculated

Our web collection on statistics for biologists contains articles on many of the points above.

Software and code

Policy information about availability of computer code

Data collection	BWA-mem version 0.7.17, STAR Align version 2.7.8a
Data analysis	Genome Analysis Toolkits (GATK) HaplotypeCaller version 3.7, Sequenza version 3.0.0, CNVKit version 0.9.9, HRDex version 0.0.0.9, Mutect2 version 4.1.2, ANNOVAR version 2018-04-16, OncoKB, MSISensor version 0.6, FitMS version 2.3.0, HTSeq-Count version 2.0.3, DESeq2 version 1.38.3, GSEA version 4.2.3, CIBERSORTx, MCPCounter, xCell

For manuscripts utilizing custom algorithms or software that are central to the research but not yet described in published literature, software must be made available to editors and reviewers. We strongly encourage code deposition in a community repository (e.g. GitHub). See the Nature Portfolio guidelines for submitting code & software for further information.

Data

Policy information about availability of data

All manuscripts must include a data availability statement. This statement should provide the following information, where applicable:

- Accession codes, unique identifiers, or web links for publicly available datasets
- A description of any restrictions on data availability
- For clinical datasets or third party data, please ensure that the statement adheres to our policy

The individual-level clinical data are available under restricted access due to IRB constraints, access can be obtained by contacting the authors. The data generated from our analyses are included in the manuscript main text, tables, and figures and online Supplementary Materials (available online). The RNA sequencing data is

deposited in the Gene Expression Omnibus (GEO) repository under accession number GSE306117 (https://www.ncbi.nlm.nih.gov/geo/query/acc.cgi?acc=GSE306117). Tumor genomic sequencing data is deposited in the dbGaP database entitled LFS Genomics under accession number phs003348 (https://dbgap.ncbi.nlm.nih.gov/beta/study/phs003348.v1.p1/#study).

Research involving human participants, their data, or biological material

Policy information about studies with human participants or human data. See also policy information about sex, gender (identity/presentation), and sexual orientation and race, ethnicity and racism.

Reporting on sex and gender	We have used the termionology “female” to refer to female sex as a biological attribute. Gender identity was not reported for these patients.
Reporting on race, ethnicity, or other socially relevant groupings	We report our patient cohort “Self-identified Race” using the categories Caucasian, African American, Asian, Mixed Race, Other from the electronic health record. Our genomic analyses were not controlled for self-identified race.
Population characteristics	Table 1 includes population characteristics of our cohort.
Recruitment	Patients were recruited at the University of Pennsylvania either in the Cancer Risk Evaluation Program LFS Biobank (NCT04367246) (LFS cohort) or any clinical location consenting to the Penn Medicine Biobank (non-LFS cohort).
Ethics oversight	Acquisition of LFS patient blood and tumor samples was approved by the Institutional Review Boards of the University of Pennsylvania and Children’s Hospital of Philadelphia (CHOP) (Penn IRB#834147/CHOP IRB#18-015810). Acquisition of patient blood and tumor samples (PMBB) was approved by the Institutional Review Board of the University of Pennsylvania (Penn IRB#832122).. Informed consent was obtained from each participant for use of their samples and clinical data in genetic and immunologic studies.

Note that full information on the approval of the study protocol must also be provided in the manuscript.

Field-specific reporting

Please select the one below that is the best fit for your research. If you are not sure, read the appropriate sections before making your selection.

☒ Life sciences ☐ Behavioural & social sciences ☐ Ecological, evolutionary & environmental sciences

For a reference copy of the document with all sections, see nature.com/documents/nr-reporting-summary-flat.pdf

Life sciences study design

All studies must disclose on these points even when the disclosure is negative.

Sample size	As the study reports on a rare genetic condition, sample size of the LFS cohort was defined by the number of patient samples we could obtain. Sample size for the control non-LFS cohort was defined to match the LFS cohort size.
Data exclusions	No data exclusions.
Replication	Each group consisted of multiple "replicates" with regards to patients. Give the expense, sequencing runs were not repeated.
Randomization	Patients with LFS defined by having a specific inherited genetic change in TP53 comprised the LFS cohort; the control nonLFS cohort was defined as patients with no genetic change in 44 cancer risk gene.s
Blinding	Bioinformaticians were blinded to sample cohort during initial data analysis (alignment, variant calling, variant classification).

Reporting for specific materials, systems and methods

We require information from authors about some types of materials, experimental systems and methods used in many studies. Here, indicate whether each material, system or method listed is relevant to your study. If you are not sure if a list item applies to your research, read the appropriate section before selecting a response.

Materials & experimental systems

n/a	Involved in the study
☐	☒ Antibodies
☒	☐ Eukaryotic cell lines
☒	☐ Palaeontology and archaeology
☒	☐ Animals and other organisms
☐	☒ Clinical data
☒	☐ Dual use research of concern
☒	☐ Plants

Methods

n/a	Involved in the study
☒	☐ ChIP-seq
☒	☐ Flow cytometry
☒	☐ MRI-based neuroimaging

Antibodies

Antibodies used

P53: Clone DO-7, Dako, Carpinteria, CA, Catalog No: M7001
CD8; Clone C8, Dako, Carpinteria, CA, Catalog No: M7103
CD20: Clone L26, Dako, Carpinteria, CA, Catalog No: M0755
CD68: Clone KP1, Dako, Carpinteria, CA, Catalog No: M0814
CD3: Clone LN10, Leica Microsystems, San Francisco, CA, Catalog No: PA0553
CD4: Clone RM/EP204, Biocare Medical, Pacheco, CA, Catalog No: 3209
FoxP3: Clone 206D, Biolegend Antibodies, San Diego, CA, Catalog No: 20102
Cytokeratin Alexa-488 conjugated: Clone AE1/AE3, ThermoFisher Catalog No: 53-9003-82
CD4 Rabbit monoclonal: Clone SP35, SpringBio Catalog No: M3350
CD8 Mouse monoclonal: Clone C8/144B, Dako Catalog No: IR62361-2
CD20 Mouse monoclonal: Clone L26, Dako Catalog No: GA60461-2
CD3 Rabbit monoclonal: Clone SP7, Novus Catalog No: NB600-1441SS
Ki-67 Mouse Monoclonal: Clone MIB-1, Dako Catalog No: GA62661-2
Granzyme B Mouse Monoclonal: Clone 4E6, Abcam Catalog No: ab139354

Validation

All antibodies were validated in the laboratory against known positive and negative control tissues.

Clinical data

Policy information about clinical studies
All manuscripts should comply with the ICMJE guidelines for publication of clinical research and a completed CONSORT checklist must be included with all submissions.

Clinical trial registration

NCT04367246

Study protocol

Study protocol is available upon request.

Data collection

Sequencing data were generated from archival tumor blocks and germline blood obtained from patients recruited in clinic.

Outcomes

N/A

Plants

Seed stocks

N/A

Novel plant genotypes

N/A

Authentication

N/A
