# Supplementary material for: Distinct genomic and immunologic tumor evolution in germline TP53-driven breast cancers
Source: Nat Commun. 2026 May 21;17:7300. doi: 10.1038/s41467-026-73163-4 (PMC13402668; doi:10.1038/s41467-026-73163-4)
Supplement: Supplementary file 5 — Transparent Peer Review File [file 41467_2026_73163_MOESM5_ESM.pdf]

nature portfolio

Peer Review File

Distinct genomic and immunologic tumor evolution in germline TP53-driven breast cancers

Corresponding Author: Dr Kara Maxwell

This file contains all reviewer reports in order by version, followed by all author rebuttals in order by version.

Version 0:

Reviewer comments:

Reviewer #1

(Remarks to the Author)
The authors report a comprehensive analysis of gTP53 related breast cancers compared to selected controls. They report that loss of the second functional copy of TP53 appears to be a very early event leading to TP53 being the driver behind these tumors in contrast to somatic TP53 mutated breast cancers. Overall however, this comes across as a study where lots of things have been added on as time has gone on and then a story has tried to be made out of rather than coming across as a coherent study. There is an awful lot of padding out with extraneous information about VUS/LB/B patients and all that work up making it very hard to work out what is going on. In the end, the authors have 21 actual LFS breast tumours, 2 from TCGA (only two in Table S3 from TCGA actually had TP53 PGV and breast cancer), with validation in 17 from MSK.

I would suggest stripping this paper right back - here is our genomic and immunology study of 21 (or add in the MSK) LFS associated breast tumours and this is what was found. The way it is currently presented is a soup of information a lot of which does not add to the coherence of the message and the reader is left trying to piece it together. They have done loads and loads of work and I just wonder if the overall strategy and 'why' had enough attention paid to it. Eg in all that TCGA work - there were two breast cancers

Specific points

1. 'Pathogenic 5 germline variants (PGVs) in TP53 occur in approximately 1:5000 people and lead to the Li 6 Fraumeni Syndrome (LFS) spectrum in which individuals have an up to 90% risk of developing cancer over their lifetimes4' =please provide original source reference for 1 in 5000. Ref 4 gives a much higher estimate based on a permissive assessment of gTP53
2. 'Typically, LFS-BC are ER+; over half are HER2+ and triple negative BC (TNBC) is rarely found in LFS females6,9' The authors should also reference the original publication confirming the typical triple positive pathology (Wilson et al 2010) as well as the high rate of high grade comedo DCIS
3. The authors have assessed acquired somatic TP53 variants in the non LFS cohorts but do not appear to report this in the germline TP53 cohort. Previous work in childhood ACC showed multiple acquiredTP53 variants often with loss of germline. This analysis should also be reported on the TP53 cohort rather than just staining and LOH. It looks like the data is available in (Table S1/S2) as results are given in those tables.
4. A lot of the results are presented as percentages - should give the actual numerator and denominator as well so you know exactly how many tumours were analysed
5. There is a lot of referring to their own group's papers or papers where authors are co-authors on those papers, rather than to more pertinent studies.

Reviewer #2

(Remarks to the Author)
This manuscript presents fundamental and first of its kind data on LFS-associated, TP53-driven BC compared to sporadic early-onset BC. Even though LFS-associated BC only represents a small fraction of overall BC burden, this analysis potentially yields overall significance to the field of cancer biology, TP53 biallelic loss being one of the most unifying events

in tumor evolution. The manuscript is well-written, the methods look rigorous, the quality of experiments and data analyses is sound, and figures and tables are superbly presented. The small sample size limiting statistical power is a primary weakness of this manuscript; however, the authors acknowledge this and note that additional studies will be required to confirm the findings.

Major comments

Currently, the G334R variant – although located in the tetramerization domain – is a VUS by TP53-VCEP assessment (BS2, BS3, PM2_supporting, PS4, PP1_moderate), and it will likely stay so for the time being. To keep the data clean and refering to P/LP variants only, I would suggest removing this variant and its associated tumors from the dataset. Could you please also comment on how the pathogenicity status of TP53 germline variants was assessed (Fortuno criteria? ClinVar?)?

Minor comments

The numbers of tumors analyzed in the manuscript (p.5 l.20) don't match up with Fig. S1 (how many tumors in how many patients, 15 or 16 surrounding normal tissues?). The S1 legend states that the LFS BC cohort contained nine HER2+ IDC, whereas the Figure states 12 HER2+ IDC. Please check and verify.

P.6 l.14: In the TCGA-LFS cohort, 58% of tumors 'with TP53 P/LP variants' had LOH and 13% 15 had an acquired TP53 somatic mutation (71% biallelic loss rate).

P.7 l.17: Please check the normalized ratios 0.25-16 and <1 vs. >1

Additionally, the following spelling errors were noted:
P.8 l.14: nonLFS-BC
P.9 l.1: acquired TP53 mutations

After revision, I strongly recommend this article for acceptance and look forward to reading the final version.

Reviewer #3

(Remarks to the Author)
Review:
In the manuscript entitled 'Distinct genomic and immunologic tumor evolution in germline TP53-driven breast cancers'

LF – li Fraumeni syndrom harbors germline pathogenic TP53 variant and increases risk of developing cancer with 90%. The authors aim to address an important and potentially clinically interesting question: is Li Fraumeni syndrome (LFS-BC) TP53 associated breast cancer different phenotypically from early onset sporadic breast cancer? The authors point to a particularly interesting observation, namely that despite carrying TP53 alterations, which is often associated with triple negative breast cancer (TNBC), the LFS-BC are almost exclusively ER positive.

Did the authors reply to these questions?
If the aim is to study the impact of germline TP53 pathogenic variants on breast cancer vs acquired TP53 mutations, then the authors should have compared the LF-BC to only those sporadic cases that also harbored somatically acquired nutation and used the rest as some type of wt control. I do not see such analysis, all sporadic cancers are analyzed in one group.

The manuscript is well-organized with clear sections. The introduction provides solid background, but some key references are missing (PMID: 29147603). For example the association of TP53 to immune infiltration has been addressed in the context of breast cancer directly PMID: 25351767 and the significance of immune infiltration in TP53mut cases on ER+ and ER- background is specifically addressed. The methods employed for genomic, transcriptomic, and immune profiling are robust and well-detailed, ensuring reproducibility.

Readability can be improved by summarizing key findings in tables or by highlighting the most significant findings and their implications more clearly. Some sections could benefit from a brief introductory sentence to set the context. For example my understanding is that the key findings are that while LFS-BC harbor structural losses (biallelic) in the TP53 gene, while point mutations are more often observed in the sporadic cases (looks like from figure 1). The biallelic losses are associated with specific genomic rearrangements and maybe specific tumor infiltration, both quantitatively and QUALITATIVELY. If this is the main finding the paper should be written to highlight this line of observations. The question of the ER positive however remains unanswered.

Apart from that it points to that structural losses (bialletic) in the TP53 gene, while point mutations are more often observed in the sporadic cases, I do not see the value of Figure 1: it creates an illusion of great dichotomy, but in fact it is mostly contributed by the supervised division of cases (LFS-BC vs sporadic) and the biallelic loss, exclusively in LFS-BC. All other differences are difficult to catch (too many colors) and estimate the significance of: are these unsupervised clusters? Is there indeed significance in distribution of pathways? As I said at the start, interesting to highlight the mutated sporadic breast tumors compared to those arising from the germ-line. Also, many of the suggested trends are in fact not significant (Figure 2).

The discussion can be improved by reflecting more of the results presented. Currently, some critical findings are not adequately discussed. For instance, the unique oncogenic alterations identified in LFS-BC, including the lower frequency of recurrent oncogenic mutations and the specific focal amplifications observed (ERBB2, CDK12) can be discussed to clearly underline the relevance of these findings. Additionally, the prevalence of specific mutational signatures (SBS1, SBS3, SBS5, SBS18) in LFS-BC and their potential impact on tumor biology and treatment response should be included in the discussion section. Why are these mutational signatures not investigated and compared to none-LFS BC? Furthermore, the differentially expressed genes and pathways , particularly for the downregulated p53 target genes (eg BAX) and MYC pathway in LFS-BC should be elaborated in the context of the p53 biallelic loss. Mutations in ESR signaling pathway was found in more than 50% of non-LFS compared to 14% in LFS BC. This finding also warrants more in-depth discussion. Is the ESR signaling associated to germline TP53 mutation driving the early onset of BC in LFS?

Specific points / corrections:

Page 7, line6: Non LFS BC reported to have recurrent oncogenic mutations in PIK3CA, GATA3 and MAP3K1. MAP3K1 is not to be seen as recurrent in figure S2C / figure 1a

Page 7, line 14 : Authors report amplifications in LFS-BC for ERBB2 and CDK12 and point to figure S2d. These amplifications in LFS BC are nicely presented in figure 1, but not in supplementary figure S2d?
Page 7 line 19: Authors claim that all LFS BC has co-amplification of ERBB2 and CDK12 (Table S2). According to supplementary table 2 this is true for 12 out of 21 LFS-BC. CDK12 is listed as amplified for 12 LFS-BC, not all in the table S2.

In figure 2b, panel chromosomal instability scores the lines indicating ‘ns’ are misplaced
Gene expression – bulk RNA seq

Page 9 line 24/25: authors claim clustering based on hormonal receptor subtypes. True for normal and DCIS, however, this is not apparent for HER2+/- . Authors should consider to re-phrase

Reviewer #4

(Remarks to the Author)
I co-reviewed this manuscript with one of the reviewers who provided the listed reports. This is part of the Nature Communications initiative to facilitate training in peer review and to provide appropriate recognition for Early Career Researchers who co-review manuscripts.

Reviewer #5

(Remarks to the Author)
The authors of the manuscript entitled "Distinct genomic and immunologic tumor evolution in germline TP53-driven breast cancers" performed a multimodal analysis of LFS breast cancer (LFS-BC) compared to sporadic premenopausal BC and evaluated the landscape of mutant p53-driven tumorigenesis in breast tissue using a variety of methods. They revealed that nearly all LFS-BC underwent biallelic loss of TP53 without recurrent oncogenic variants (except ERBB2 amplification) and that compared to sporadic BC, in situ and invasive LFS-BC exhibited a high burden of short amplified aneuploid segments. Besides, the role of the tumor microenvironment has not been studied previously in detail in this population and this aspect on this study provides additional insights. The study is novel and is of significance to the field, since it is the largest LFS-BC cohort being described in such a comprehensive manner. However the small sample size, which precludes additional subtype analysis, remains a limitation of the study. Overall, the following points need to be addressed:

Methods
1. The authors used multiplex IF panels in order to further characterize the immune profile and lymphocytic activation status. They briefly describe the methodology but they need to expand more on some details in the methods part. How was the imaging and signal analysis was performed? How many FOVs were analysed for each patient/slides? For which analyses have the authors used the inForm software and how did they combine with AQUA? How was the expression in the different tissue compartments analyzed (DAPI for all cells/whole tissue expression or only for tumor tissue as it is stated)?
2. The authors need to provide details on the primary and secondary antibodies used (clones, dilution, fluorophores) in the text and also in the reporting summary.
3. The authors need to provide more details on how they performed the immune cell quantification (e.g. which areas) in LFS-DCIS. How did this quantification related/correlate (if it does) to the recommendations from the International Immuno-Oncology Biomarker Working Group on Breast Cancer?
4. For the IHC quantification analyses, the authors could provide the specific QuPath parameters and possibly some representative images in the Supplementary Material, as well as some previous reference on quantifying these markers with this software.

Results:
5. The authors demonstrated that LFS-BC had lower CD8+ T-cell infiltration compared to sporadic BC yet higher levels of proliferating cytotoxic T-cells. They can also test the transcriptomic immune profiling (using e.g. CIBERSORT, MCPCounter)

in tumors with TP53 germline vs somatic TP53 mutations in the TCGA.
6. Given the previously described role of the somatic TP53 mutations and tumor microenvironment in breast cancer (Zhou X et al. Br J Cancer 2022), the authors could consider demonstrate the immune response and components in TCGA (using e.g. CIBERSORT, MCPcounter) in patients with TP53 mut vs TP53 wt tumors. The authors could also consider investigate the correlation of CNA burden and immune response in those tumors (although it is will be in a small number of patients).
7. The observation that LFS-BC -compared to sporadic BC- had higher levels of proliferating cytotoxic T-cells, despite a lower CD8+ T-cell infiltration is interesting, and the authors can further explore if any other (immune-related) genes contribute to this phenotype (e.g. GSEA in these tumors).
8. When evaluating the tumor microenvironment profiling in DCIS, why did the authors choose to analyze separately the node-positive versus node-negative IDC?
9. Given that the authors have confined the DCIS analysis in LFS, they could consider comparing the TME between LFS-DCIS and non-LFS DCIS (and probably also paired analyses with non-LFS IDC).
10. The authors need to provide a summary sentence of the main findings at the end of each part, since this is going to improve the flow and clarity of the text

Discussion:
11. The authors need to discuss the results on the immunological aspects and microenvironment in light of the recently recognized role of germline alterations in immunoediting (Houlahan et al. Science 2024, Samayan Immunity) and relate these aspects to their findings (e.g. germline variants could be also a source of immunogenic epitopes).
12. The authors could consider discuss more on the role of CNA burden, aneuploidy and immune response, as they briefly touch upon this aspect when discussing that LFS-BC had lower CD8+ T-cells than non-LFS-BC (Davoli T et al Science 2017). Indeed, a growing body of literature support the inverse link of higher somatic CNA with dampened immune response in BC (Karn T et al. JAMA Oncology 2017; Zerdes I et al. npc Breast Cancer 2021).
13. The authors could also add on the aspects of the immune microenvironment on progression from DCIS to invasive cancer in sporadic BC and the immunosuppressive TME (Gil Del Alcazar CR et al. Cancer Discovery 2017 , Strell C. et al. Eur J Cancer 2022)

Version 1:

Reviewer comments:

Reviewer #1

(Remarks to the Author)
the authors have addressed my specific points well but ignored my general point about reorganising the manuscript. as there are 4 other reviewers and these have not picked up on this point i am prepared to let this ride.

Reviewer #2

(Remarks to the Author)
Remarks to be addressed:

As the quality of the cohort and, thus, the entire study depends on the actual pathogenicity of the detected germline TP53 variants, it is essential for the manuscript to add the information that pathogenicity status of TP53 germline variants was assessed via ClinVar and that variants with conflicting interpretations were included. You could easily add this information in the methods section - Cohort ascertainment (line 447) - "Inclusion criteria for LFS-BC were 1) clinically confirmed TP53 PGV as per ClinVar assessment, including those with conflicting interpretations;...".

lines 126-131: "Tumors with sufficient material for p53 IHC and targeted NGS analysis were available for 21 LFS-BC, 15 surrounding normal and 11 contralateral normal specimens (Figure S1). Among these, eleven cases had DNA binding domain mutations, including known dominant negative loss of function mutations (G245S, R248Q, R110L) and putative hypomorphic mutations (T125M, R181H, P151S, C141Y), seven had loss of function mutations, and four had tetramerization domain mutations (G334R, R337C, R337H) (Figure 1A, Table S1). ----
1) Please specify in this section that all tumor samples were derived from LFS individuals with verified GERMLINE TP53 variant, e.g. "Among these, eleven cases had confirmed germline DNA binding domain mutations...".
2) line 131: "...and four had tetramerization domain mutations (G334R, R337C, R337H)" ----Please add that two of the latter variants are also putative hypomorphs (in addition to the previously named ones in the DBD).
3) 21 LFS-BC --> 11 + 7 + 4 = 22 variants (unless one individual had two germline variants, this doesn't add up -- if this should be the case, please specify somewhere in the manuscript). Figure 1A suggests that 10 (instead of 11) cases had DBD variants. Please check.
4) Please also detail in this section the number of patients from which the tumors were acquired. Please clarify if by "cases" you refer to tumor samples or to patients.

lines 454-456: "Specimens from twenty-six LFS-BC (46%) from 19 patients were obtained, of which surrounding normal breast tissue (SNBT) and contralateral normal breast tissue (CNBT) were available for analysis in 15 cases and 11 cases respectively (Figure S1, Table S1)."
--- These numbers don't match up with Table S1 and Figure S1. Table S1 relates to 28 LFS-BC from 22 patients, and only

19 LFS-BC from 16 patients seem to have had sufficient material for IHC and targeted NGS analysis as per columns O and R. Figure S1 relates to 27 LFS-BC from 21 patients. Figure 1A and the results section line 127 refers to 21 LFS-BC.

Figure S1 legend: "The LFS BC cohort contained six ER+Her2- IDC, 12 HER2+ IDC, nine DCIS, 16 (!!) surrounding normal, 11 contralateral normal tissue samples from 21 patients; blood specimens were available from all 22 (!!) patients. The early onset non LFS BC cohort contained 14 ER+Her2 BC, five HER2+ BC and 10 TNBC, and 25 surrounding normal tissue samples from 29 (!!) patients with matched normal blood samples."
---- Numbers included in Figure S1 don't match up with numbers in the legend below. Legend still states 16 (not 15) surrounding normal samples. 21 vs. 22 patients with available blood specimens and PGV in LFS group. 27 vs. 29 patients in control group. Also discrepancies with the main manuscript results and methods section (see above).

Altogether, the numbers urgently need to be clarified and to be consistent throughout the manuscript, which could potentially necessitate reassessment of statistical evaluation.
It needs to become clear to the reader 1) how many LFS-BC from 2) how many individuals with 3) how many different TP53 variants were part of the final analysis. Please also indicate in Table S1 which of the LFS-BC samples were included in the final analysis and which were left out due to insufficient material.

Reviewer #4

(Remarks to the Author)
We jointly agree that the authors have replied satisfactory to the raised questions, both by us and the other reviewers.

Reviewer #5

(Remarks to the Author)
The authors have successfully addressed my comments and provided additional information that overall improved the manuscript, results' presentation and readability.

Version 2:

Reviewer comments:

Reviewer #2

(Remarks to the Author)
The authors have addressed my comments and provided clarifying information as requested.

Open Access This Peer Review File is licensed under a Creative Commons Attribution 4.0 International License, which permits use, sharing, adaptation, distribution and reproduction in any medium or format, as long as you give appropriate credit to the original author(s) and the source, provide a link to the Creative Commons license, and indicate if changes were made.
In cases where reviewers are anonymous, credit should be given to 'Anonymous Referee' and the source.

The images or other third party material in this Peer Review File are included in the article's Creative Commons license, unless indicated otherwise in a credit line to the material. If material is not included in the article's Creative Commons license and your intended use is not permitted by statutory regulation or exceeds the permitted use, you will need to obtain permission directly from the copyright holder.
To view a copy of this license, visit https://creativecommons.org/licenses/by/4.0/

Response to Reviewers

We would like to thank the Editors and Reviewers of *Nature Communications* for their enthusiastic comments and thoughtful review of our manuscript NCOMMS-24-23316. We address each of the comments and suggestions in point by-point responses below. Page (P) and Line numbers (L) refer to the Tracked Changes version of the manuscript. We feel that the suggested changes have considerably improved the paper.

<u>A. Reviewer #1 (Remarks to the Author)</u>

1. 'Pathogenic 5 germline variants (PGVs) in TP53 occur in approximately 1:5000 people and lead to the Li 6 Fraumeni Syndrome (LFS) spectrum in which individuals have an up to 90% risk of developing cancer over their lifetimes4' =please provide original source reference for 1 in 5000. Ref 4 gives a much higher estimate based on a permissive assessment of gTP53

We appreciate the clarification and have changed the text to provide the exact figures from Reference 4 instead of an estimation and have referenced prior publications on *TP53* population frequency estimates (Gonzalez et al 2009 and Lalloo et al 2003, Reference #5-6) on Page 4 Lines 5-7:

> "Pathogenic germline variants (PGVs) in *TP53* occur in approximately 1:3000 to 1:10000 people and lead to the Li Fraumeni Syndrome (LFS) spectrum in which individuals have an up to 90% risk of developing cancer over their lifetimes[4–6]."

2. 'Typically, LFS-BC are ER+; over half are HER2+ and triple negative BC (TNBC) is rarely found in LFS females6,9' The authors should also reference the original publication confirming the typical triple positive pathology (Wilson et al 2010) as well as the high rate of high grade comedo DCIS

We thank the reviewer for suggesting these helpful references. We have added these references to the Introduction, specifically we added the Wilson et al 2010 (Reference #15) to the references cited on Page 4, Line 12. We have also added a line to highlight the association of germline *TP53* mutations with high grade DCIS on Page 4., Line 13-14.:

> "In pre-invasive breast cancers in LFS patients, *TP53* mutations are associated with high-grade histology[14]."

3. The authors have assessed acquired somatic TP53 variants in the non LFS cohorts but do not appear to report this in the germline TP53 cohort. Previous work in childhood ACC showed multiple acquiredTP53 variants often with loss of germline. This analysis should also be reported on the TP53 cohort rather than just staining and LOH. It looks like the data is available in (Table S1/S2) as results are given in those tables.

We have added more details to the section of the Results where we describe that four of the LFS-BC did have an acquired *TP53* mutation as the second hit. Specifically on Page 6, Lines 7-11:

> "Sixteen (76%,16/21) of LFS-BC had locus-specific LOH and four (19%,4/21) had an acquired *TP53* somatic mutation (Figure 1b, Table 2, Table S2). Our study identified acquired *TP53* mutations in the DCIS samples (n=3), specifically p.L265Rfs*80, p.G154V, and p.Y163S, while the one IDC sample exhibited a p.N247I *TP53* mutation. No surrounding and contralateral tissue had any acquired *TP53* mutation."

4. *A lot of the results are presented as percentages - should give the actual numerator and denominator as well so you know exactly how many tumours were analysed*

Thank you for this suggestion as this does increase the accuracy of the result reporting. Throughout the Results section, we have added actual numerator and denominators.

5. There is a lot of referring to their own group's papers or papers where authors are co-authors on those papers, rather than to more pertinent studies.

Thank you for your observation. In our revised version, we have made sure to incorporate a broader range of important papers. We hope this addresses your concern.

Reviewer #2 (Remarks to the Author)

Major comments
1. Currently, the G334R variant – although located in the tetramerization domain – is a VUS by TP53-VCEP assessment (BS2, BS3, PM2_supporting, PS4, PP1_moderate), and it will likely stay so for the time being. To keep the data clean and refering to P/LP variants only, I would suggest removing this variant and its associated tumors from the dataset. Could you please also comment on how the pathogenicity status of TP53 germline variants was assessed (Fortuno criteria? ClinVar?)?

While we are aware that the p.G334R variant is considered a VUS by the *TP53* VCEP, our eligibility criteria were individuals who had a *TP53* pathogenic germline variant as per Clinvar, including those with conflicting interpretations. The *TP53* p.G334R variant is considered Likely Pathogenic by Ambry, Invitae and Myriad among other laboratories (https://www.ncbi.nlm.nih.gov/clinvar/variation/182969/) and so was included, in addition to other hypomorphic *TP53* variants as per Page 6, Lines 2-6.

Minor comments
2. The numbers of tumors analyzed in the manuscript (p.5 l.20) don't match up with Fig. S1 (how many tumors in how many patients, 15 or 16 surrounding normal tissues?).

Thank you for pointing this out. You are correct, there is an inconsistency between the manuscript and Figure S1. The correct number is 15, not 16, as stated in Supplementary Figure 1. We will ensure this correction is made in the revised version. We appreciate your attention to detail.

3. The S1 legend states that the LFS BC cohort contained nine HER2+ IDC, whereas the Figure states 12 HER2+ IDC. Please check and verify.

Thank you again for highlighting this discrepancy. The correct breakdown is 12 HER2+ IDC cases, with 9 being ER+HER2+ and 3 being ER-HER2+. We have corrected this error in the Supplementary Figure 1 legend in the revised version. We appreciate your careful review.

4. P.6 l.14: In the TCGA-LFS cohort, 58% of tumors 'with TP53 P/LP variants' had LOH and 13% 15 had an acquired TP53 somatic mutation (71% biallelic loss rate).

These numbers are accurate.

5. P.7 l.17: Please check the normalized ratios 0.25-16 and <1 vs. >1

The ratios provided have been verified and are correct as presented.

6. Additionally, the following spelling errors were noted:
P.8 l.14: nonLFS-BC
P.9 l.1: acquired TP53 mutations

Thank you for bringing the spelling errors to our attention. We have corrected "nonLFS-BC" and "acquired TP53 mutations" in the manuscript. These revisions will be reflected in the updated version. We appreciate your careful review.

C. Reviewer #3 (Remarks to the Author)

1. In the manuscript entitled 'Distinct genomic and immunologic tumor evolution in germline TP53-driven breast cancers' LFS – li Fraumeni syndrome harbors germline pathogenic TP53 variant and increases risk of developing cancer with 90%. The authors aim to address an important and potentially clinically interesting question: is Li Fraumeni syndrome (LFS-BC) TP53 associated breast cancer different phenotypically from early onset sporadic breast cancer? The authors point to a particularly interesting observation, namely that despite carrying TP53 alterations, which is often associated with triple negative breast cancer (TNBC), the LFS-BC are almost exclusively ER positive. Did the authors reply to these questions? If the aim is to study the impact of germline TP53 pathogenic variants on breast cancer vs acquired TP53 mutations, then the authors should have compared the LF-BC to only those sporadic cases that also harbored somatically acquired nutation and used the rest as some type of wt control. I do not see such analysis, all sporadic cancers are analyzed in one group.

The goal of this study was to compare breast cancer development in individuals with a germline *TP53* mutation compared to age-matched individuals without a germline *TP53* mutation. We have clarified this in the Introduction on Page 5, Lines 1-3.

> "To date, there are no published data analyzing differences in the genomic, transcriptomic, or immune profiles of germline mutant p53 driven BC compared to breast cancer in premenopausal women without inherited *TP53* mutations."

Unfortunately, in our nonLFS-BC cohort, we had limited numbers of p53 mutant cancers outside of TNBC. We did however recapitulate the findings that aneuploid segments and specifically short amplified aneuploid segments (AAS) were enriched in LFS-BC compared to p53 mutant nonLFS-BC and have added these findings to Figure S4 and in the text on Page 9, Lines 10-13.

> "These findings were numerically recapitulated when number of allelic imbalances and AAS were compared between LFS-BC to nonLFS-BC with an acquired *TP53* mutation, and the size of AAS was also significantly smaller in LFS-BC compared to nonLFS-BC with an acquired *TP53* mutation (Figure S4a)."

Further, aneuploid segments were specifically enriched compared to other genomic instability measures in premenopausal p53 mutant HER2+ BC compared to p53 WT in the TCGA data. We have added these findings to Figure S4 and in the text on Page 9, Lines 15-18.

> "Comparing premenopausal HER2+ BC with and without acquired *TP53* mutations, aneuploidy score, HRD score and LST score were similar, but segmental allelic imbalances and LOH were higher in p53 mutant compared to p53 WT HER2+ premenopausal BC."

2. The manuscript is well-organized with clear sections. The introduction provides solid background, but some key references are missing (PMID: 29147603). For example the association of TP53 to immune infiltration has been addressed in the context of breast cancer directly PMID: 25351767 and the significance of immune infiltration in TP53mut cases on ER+

and ER- background is specifically addressed. The methods employed for genomic, transcriptomic, and immune profiling are robust and well-detailed, ensuring reproducibility.

Thank you for your positive feedback and for highlighting the missing references. We have added more background on prior data suggesting an importance of factors such as p53 status on immune infiltrates in breast cancer into the Introduction on Page 4 Lines 24-26.:

> "Prior work has suggested the importance of age, estrogen levels and p53 mutation status on immune infiltration in breast cancer; however, these studies only addressed sporadic p53 mutant tumors[20,21]."

3. Readability can be improved <u>by summarizing key findings in tables or by highlighting</u> the most significant findings and their implications more clearly. Some sections could benefit from a brief introductory sentence to set the context.

We thank the Reviewer for this excellent suggestion. We have created a new Table 2 (Page 34) that summarizes the genomic and immunological data presented in Figures 1,2 and 4. In order to better summarize the RNAseq data, we have conducted a new analysis of differential gene expression in LFS-BC versus nonLFS-BC using Ingenuity Pathway Analysis which we have summarized in new Supplemental Figure 9 and on Page 12, Lines 1-6:

> "In order to incorporate the directionality of RNA expression differences between LFS-BC and nonLFS-BC, we conducted an analysis with Ingenuity Pathway Analysis (IPA) software. This analysis demonstrated that the p53 pathway was significantly altered in LFS-BC, with key genes regulating cell cycle arrest, *CDKN1A*, and apoptosis, *BAX*, being down-regulated in LFS-BC and genes involved in suppressing angiogenesis being up-regulated (Figure S9)."

4. Apart from that it points to that structural losses (bialletic) in the TP53 gene, while point mutations are more often observed in the sporadic cases, I do not see the value of Figure 1: it creates an illusion of great dichotomy, but in fact it is mostly contributed by the supervised division of cases (LFS-BC vs sporadic) and the biallelic loss, exclusively in LFS-BC. All other differences are difficult to catch (too many colors) and estimate the significance of: are these unsupervised clusters? Is there indeed significance in distribution of pathways? As I said at the start, interesting to highlight the mutated sporadic breast tumors compared to those arising from the germline. Also, many of the suggested trends are in fact not significant (Figure 2).

We appreciate the Reviewer's comment. In order to display the data in alternative formats, we have created a new Table 2 (see above), and we believe that Figure 1 continues to have value as a pictorial representation of the data in the tables.

5. The discussion can be improved by reflecting more of the results presented. Currently, some critical findings are not adequately discussed. For instance, the unique oncogenic alterations identified in LFS-BC, including the lower frequency of recurrent oncogenic mutations and the specific focal amplifications observed (ERBB2, CDK12) can be discussed to clearly underline the relevance of these findings.

This suggestion is similar to Reveiwer #5 points #11 and #12, and we have added additional discussion points to stress the relevance of our findings as per below.

6. Additionally, the prevalence of specific mutational signatures (SBS1, SBS3, SBS5, SBS18) in LFS-BC and their potential impact on tumor biology and treatment response should be included in the discussion section.

Thank you for your suggestion. We agree that discussing the prevalence of specific mutational signatures LFS-BC and their potential impact on tumor biology and treatment response would enhance the discussion. We have added two new sentences to the Discussion. On Page 15, Lines 20-23:

> "Our copy number mutational signature results support the hypothesis that development of aneuploid segments are an important early step in tumor development as we found that chromothripsis (ie CN5) and focal LOH-Diploid or chromosomal Instability (CN9) were common in LFS-BC."

In addition, On Page 17, Lines 8-11:

> "However, our analyses of mutational signatures and immunological differences in LFS-BC versus non-LFS BC may suggest some potentially targetable avenues for treatment. For example, nearly all LFS-BC show signatures of HRD (10/12) which may suggest that treatment with PARP inhibitors could be beneficial in LFS-BC patients[38]."

7. Why are these mutational signatures not investigated and compared to non-LFS BC?

We agree with the Reviewer that a comparison of mutational signatures by WGS in non-LFS BC would be a beneficial experiment, and this is the subject of future directions for this work.

8. Furthermore, the differentially expressed genes and pathways, particularly for the downregulated p53 target genes (eg BAX) and MYC pathway in LFS-BC should be elaborated in the context of the p53 biallelic loss.

We did not perform any 'omic comparisons between LFS-BC with and without biallelic loss given that the majority of tumors had biallelic loss.

9. Mutations in ESR signaling pathway was found in more than 50% of non-LFS compared to 14% in LFS BC. This finding also warrants more in-depth discussion. Is the ESR signaling associated to germline TP53 mutation driving the early onset of BC in LFS?

We thank the Reviewer for this insightful comment and agree that our data suggests that LFS-BC does not rely on estrogen mediated pathways for tumor growth. We have added a comment to the discussion on Page 16 Lines 8-12:

> "Genomic and expression profiling in our analysis showed that LFS-BC had less frequent activating alterations in the ESR1 pathway and, consistently, failed to upregulate estrogen responsive pathways as occurs in nonLFS-BC. Therefore, it is possible that p53 dysfunction does not rely on aberrant estrogen signaling as would be consistent with analyses in sporadic metastatic breast cancers[29]."

10. Page 7, line6: Non LFS BC reported to have recurrent oncogenic mutations in PIK3CA, GATA3 and MAP3K1. MAP3K1 is not to be seen as recurrent in figure S2C / figure 1a

Thank you for your careful review and for pointing out the discrepancy regarding the recurrent oncogenic mutations in non-LFS breast cancer. We removed MAP3K1 from the Results on Page 7 Line 16.

11. Page 7, line 14 : Authors report amplifications in LFS-BC for ERBB2 and CDK12 and point to figure S2d. These amplifications in LFS BC are nicely presented in figure 1, but not in supplementary figure S2d?

Thank you for pointing out this omission, we have added a graph showing the *CDK12* amplification status in LFS-BC to Supplementary Figure 2d.

12. Page 7 line 19: Authors claim that all LFS BC has co-amplification of ERBB2 and CDK12 (Table S2). According to supplementary table 2 this is true for 12 out of 21 LFS-BC. CDK12 is listed as amplified for 12 LFS-BC, not all in the table S2.

We agree this is incorrect and we have changed the wording from “all” to “majority” on Page 8 Line 4.

13. In figure 2b, panel chromosomal instability scores the lines indicating ‘ns’ are misplaced.
Thank you for your valuable feedback regarding Figure 2b. We have corrected this Figure in the revised manuscript.

14. Gene expression – bulk RNA seq. Page 9 line 24/25: authors claim clustering based on hormonal receptor subtypes. True for normal and DCIS, however, this is not apparent for HER2+/- . Authors should consider to re-phrase

We agree that the text as written was inaccurate and have rephrased to point out that while TNBCs form a subcluster, there is not clustering of the other HR subtypes on Page 10, Line 18.

Reviewer #4 (Remarks to the Author): Early-Career Researcher co-reviewer
I co-reviewed this manuscript with one of the reviewers who provided the listed reports. This is part of the Nature Communications initiative to facilitate training in peer review and to provide appropriate recognition for Early Career Researchers who co-review manuscripts.

Thank you for your valuable feedback on the manuscript. Your insights and comments, along with those from the other reviewer, have greatly contributed to enhancing the quality of this manuscript. Thank you once again for your collaborative approach to this peer review process.

E. Reviewer #5

1. The authors used multiplex IF panels in order to further characterize the immune profile and lymphocytic activation status. They briefly describe the methodology but they need to expand more on some details in the methods part. How was the imaging and signal analysis was performed? How many FOVs were analysed for each patient/slides? For which analyses have the authors used the inForm software and how did they combine with AQUA? How was the expression in the different tissue compartments analyzed (DAPI for all cells/whole tissue expression or only for tumor tissue as it is stated)?

We thank the reviewer for this important comment, and we have addressed it by adding detailed methodology to the Methods in the pre-existing “Multiplex immunofluorescence (mIF)” section and added two additional sections “Image acquisition and analysis using quantitative immunofluorescence (QIF)” and “Image analysis using single-cell phenotyping data” on Page 24, Lines 24-26, Page 25, Lines 1-20, Page 26, Lines 13-26 and Page 27, Lines 1-20.

> “Whole-tissue sections slides were deparaffinized and subjected to antigen retrieval using EDTA buffer (Sigma-Aldrich) pH= 8.0 and boiled for 1 hour at 96 °C in a pressure-boiling container (PT module, Lab Vision). Slides were then incubated with dual endogenous peroxidase block (#S2003: Dako) for 10 minutes at room temperature. Non-specific antigens were blocked by a 30-minute incubation in 0.3% BSA in TBST. The sequential multiplexed immunofluorescence protocol was performed using isotype-

specific primary antibodies. The primary antibodies for the tumor infiltrating lymphocytes was performed to detect epithelial tumor cells (Cytokeratin Alexa-488 conjugated, clone AE1/AE3, eBioscience), helper T-cells (CD4 Rabbit monoclonal IgG, 1:100, clone SP35, SpringBio), cytotoxic T-cells (CD8 Mouse monoclonal IgG1k, 1:250, clone C8/144B, Dako), and B-cells (CD20 Mouse monoclonal IgG2a, 1:150, clone L26, Dako) as reported[71,72]. The activation panel was performed to detect epithelial tumor cells (Cytokeratin Alexa-488 conjugated, clone AE1/AE3, eBioscience), T-cells (CD3 Rabbit monoclonal IgG, 1:100, clone SP7, Novus), cell proliferation (Ki-67 Mouse Monoclonal IgG1, 1:100, clone MIB-1, Dako) and T-cell activation (Granzyme B Mouse Monoclonal IgG2a, clone 4E6, Abcam as reported[25]. Secondary antibodies and reagents used were anti-rabbit Envision (K4003, Dako), with biotinylated tyramide/Streptavidine-Alexa750 conjugate (PerkinElmer), anti-mouse IgG1k antibody (1:100, eBioscience) with Cy3-tyramide (PerkinElmer) and anti-mouse IgG2a antibody (1:200, Abcam) with Cy5-tyramide (PerkinElmer). Nuclei were highlighted using 4',6-Diamidino-2-Phenylindole (Dapi). All multiplexed panels included elimination of residual horseradish peroxidase activity between incubations with secondary antibodies by exposing the slides twice for seven minutes to a solution containing benzoic hydrazide (0.136mg) and hydrogen peroxide (50μl). Finally, slides were mounted with ProlongGold. Both panels have been previously validated[25,58,59,62.]

Image acquisition and analysis using quantitative immunofluorescence (QIF)
The entire tumor and surrounding stromal areas were scanned using a Vectra Polaris multispectral imaging station (Akoya Biosciences). Depending on the biopsy size, each patient's sample was represented between 4 and 409 independent 20X magnification fields of view (FOVs). These images were later analyzed by quantitative measurement of the fluorescent signal using the AQUA® QIF platform (Navigate BioPharma) that enables objective and sensitive measurement of targets within user-defined tissue compartments. The QIF score of each marker was calculated by dividing the marker pixel intensity by the area of the corresponding compartment defined by the cytokeratin positivity (e.g. tumor compartment), absence of cytokeratin (e.g. stroma compartment) of the Dapi-positive cells (e.g. total tissue including tumor and stroma compartments). Scores were normalized to the exposure time at which the images were captured, allowing scores collected at different exposure times to be comparable. Finally, all the stained slides were examined and cases with staining artifacts were excluded. This data was used to measure the levels of all markers in LFS vs non-LFS and to compare DCIS vs IDC.

Image analysis using single-cell phenotyping data
The images obtained from the Vectra Polaris scan were analyzed using InForm (V.2.6.0, PerkinElmer), an automated image analysis software. For each case, a tissue/cell segmentation algorithm was created using 5–10 representative FOVs selected by a pathologist. These images were used to train the software to define background, tumor and stromal compartments based on the marker staining of these areas. Then, cell segmentation parameters were selected to identify and segment individual cells based on nuclear DAPI staining. The cell segmentation performance was controlled by direct visualization of samples by a trained observer. Next, the software was trained to recognize cell subtypes based on marker expression (CD8 for effector T cells, CD4 for helper T cells, CD20 for B-lymphocytes, cytokeratin for tumor epithelial cells and absence of these markers for stromal cells). Once the algorithm was created, it was applied to the rest of the images of each individual case. Finally, the images were reviewed using visual inspection to ascertain reproducibility and modify or exclude those

with suboptimal phenotyping. Once all the images were analyzed, the information was processed using Akoya's R package PhenoReports (https://akoyabio.github.io/phenoptrReports/index.html). This tool merges the data from all the individual field of view images of a particular case and creates a data file containing information related to individual cell events, density in each tissue compartment (number of positive cells per mm2), marker-based phenotype and spatial location within the specimen (X and Y coordinates)."

2. The authors need to provide details on the primary and secondary antibodies used (clones, dilution, fluorophores) in the text and also in the reporting summary.

As above, the antibodies details were added to the Methods section "Multiplex immunofluorescence (mIF)" specifically on Page 25, Lines 3-20:

> "The primary antibodies for the tumor infiltrating lymphocytes was performed to detect epithelial tumor cells (Cytokeratin Alexa-488 conjugated, clone AE1/AE3, eBioscience), helper T-cells (CD4 Rabbit monoclonal IgG, 1:100, clone SP35, SpringBio), cytotoxic T-cells (CD8 Mouse monoclonal IgG1k, 1:250, clone C8/144B, Dako), and B-cells (CD20 Mouse monoclonal IgG2a, 1:150, clone L26, Dako) as reported[71,72]. The activation panel was performed to detect epithelial tumor cells (Cytokeratin Alexa-488 conjugated, clone AE1/AE3, eBioscience), T-cells (CD3 Rabbit monoclonal IgG, 1:100, clone SP7, Novus), cell proliferation (Ki-67 Mouse Monoclonal IgG1, 1:100, clone MIB-1, Dako) and T-cell activation (Granzyme B Mouse Monoclonal IgG2a, clone 4E6, Abcam as reported[25]. Secondary antibodies and reagents used were anti-rabbit Envision (K4003, Dako), with biotinylated tyramide/Streptavidine-Alexa750 conjugate (PerkinElmer), anti-mouse IgG1k antibody (1:100, eBioscience) with Cy3-tyramide (PerkinElmer) and anti-mouse IgG2a antibody (1:200, Abcam) with Cy5-tyramide (PerkinElmer). Nuclei were highlighted using 4',6-Diamidino-2-Phenylindole (Dapi). All multiplexed panels included elimination of residual horseradish peroxidase activity between incubations with secondary antibodies by exposing the slides twice for seven minutes to a solution containing benzoic hydrazide (0.136mg) and hydrogen peroxide (50µl). Finally, slides were mounted with ProlongGold."

3. The authors need to provide more details on how they performed the immune cell quantification (e.g. which areas) in LFS-DCIS. How did this quantification related/correlate (if it does) to the recommendations from the International Immuno-Oncology Biomarker Working Group on Breast Cancer?

We thank the reviewer for this comment. The immune cell quantification was based on AQUA® analysis of CD4 and CD8 positive cells in the total tissue, stromal or tumor areas. The QIF score of each marker/fluorophore was calculated by dividing the marker pixel intensity by the area of the corresponding compartment using fluorescence co-localization strategies. This methodology is quantitative, objective and allows to compare different T-cell subsets across cases with different sample size and tumor/stromal proportions or content. The assessment of TILs by *the International Immuno-Oncology Biomarker Working Group on Breast Cancer* is based on the visual estimation of the percentage of all mononuclear leukocytes with morphology similar to T-cells that can include NK/NKT cells and other cell subpopulations using Hematoxylin-Eosin preparations. Given the substantial differences between our QIF studies and the H&E based estimations and the expected differences, we did not compare the results from these two methodologies.

4. For the IHC quantification analyses, the authors could provide the specific QuPath parameters and possibly some representative images in the Supplementary Material, as well as some previous reference on quantifying these markers with this software.

Thank you for your constructive suggestion regarding the IHC quantification analyses. We agree that providing specific QuPath parameters and representative images would enhance the clarity and reproducibility of our methods. In the revised manuscript, we will include a detailed description of the QuPath parameters used for quantification in the Methods section on Page 24, Lines 6-11 and include a new Supplementary Figure 15 with an example image.

> "In QuPath, we first annotated the main region of interest tumor/normal as annotated by Breast pathologists. Then we used the cell detection command to identify cells within the tumor boundary. Next, we annotated regions containing different cell types tumor/normal cells/ stroma, trained a cell classifier based on these annotations, saved the classifier, and applied it to the cells. Finally, we applied intensity classification to assess staining and view the results (Figure S15)"

5. The authors demonstrated that LFS-BC had lower CD8+ T-cell infiltration compared to sporadic BC yet higher levels of proliferating cytotoxic T-cells. They can also test the transcriptomic immune profiling (using e.g. CIBERSORT, MCPCounter) in tumors with TP53 germline vs somatic TP53 mutations in the TCGA.
6. Given the previously described role of the somatic TP53 mutations and tumor microenvironment in breast cancer (Zhou X et al. Br J Cancer 2022), the authors could consider demonstrate the immune response and components in TCGA (using e.g. CIBERSORT, MCPcounter) in patients with TP53 mut vs TP53 wt tumors.

Prior studies have been performed using CIBERSORT on TCGA breast cancer data stratified by p53 mutation status and are consistent with the results of our study, specifically in the observation that LFS breast cancer have enrichment of Tregs and macrophages. We have added this observation to the Discussion on Page 16 Lines 23-26:

> "Our finding of enrichment of FoxP3+ Tregs and CD68+ macrophages is consistent with a prior CIBERSORT analysis of TCGA data showing that acquired *TP53* mutations promoted infiltration of Tregs, T helper calls and M0-type macrophages[35]"

6.The authors could also consider investigate the correlation of CNA burden and immune response in those tumors (although it is will be in a small number of patients).

We agree that determining whether CNA is associated with the immune response is an intriguing question. We have analyzed our data and did not see a significant correlation between CAN burden and immune cell population enrichment; however, as this could be due to small sample size, this is an important future direction. We have added this into the Discussion on Page 17 Lines 3-5:

> "It is possible that these observations are related to copy number burden or aneuploidy; however, our sample size limited this analysis."

7. The observation that LFS-BC -compared to sporadic BC- had higher levels of proliferating cytotoxic T-cells, despite a lower CD8+ T-cell infiltration is interesting, and the authors can further explore if any other (immune-related) genes contribute to this phenotype (e.g. GSEA in these tumors).
Our GSEA analysis of LFS-BC compared to nonLFS-BC did not show enrichment of any immune related HALLMARK pathways (Table S15). LFS-BC tumor did show upregulation of interferon alpha and interferon gamma response pathways, but this was also seen in sporadic

TP53 mutant breast cancer (Table S16) and therefore is not likely specific to LFS related breast cancers.

8. When evaluating the tumor microenvironment profiling in DCIS, why did the authors choose to analyze separately the node-positive versus node-negative IDC?

The purpose of this analysis was to investigate whether immune microenvironment changes were associated with increasing stage, therefore, we separated node negative versus node positive breast cancer.

9. Given that the authors have confined the DCIS analysis in LFS, they could consider comparing the TME between LFS-DCIS and non-LFS DCIS (and probably also paired analyses with non-LFS IDC).

Thank you for your suggestion regarding the comparison of the tumor microenvironment (TME) between LFS-DCIS and non-LFS DCIS, as well as the potential for paired analyses with non-LFS IDC. Unfortunately, we did not have access to the necessary samples to conduct these comparisons.

10. The authors need to provide a summary sentence of the main findings at the end of each part, since this is going to improve the flow and clarity of the text

We thank the Reviewers for this excellent suggestion to improve readability. We have added/edited the following summary sentences to each section:

Oncogenic Alterations in LFS versus non-LFS breast cancer (Page 8, Lines 4-6)

> "In summary, LFS-BC nearly always underwent biallelic loss of *TP53*, rarely had oncogenic mutations in other genes but were enriched for focal amplifications, particularly in *ERBB2* (Table 2)."

Genomic instability in LFS breast cancer (Page 9, Lines 18-21)

> "These findings demonstrate that enrichment of all levels of genomic instability occurs with acquired *TP53* mutations across BC; however, there is a specific enrichment of aneuploid segments in premenopausal p53 mutant tumors, specifically short AAS in LFS-BC tumors (Table 2).

Gene expression patterns in LFS breast cancer (Page 12, Lines 1-6)

> "In order to incorporate the directionality of RNA expression differences between LFS-BC and nonLFS-BC, we conducted an analysis with Ingenuity Pathway Analysis (IPA) software. This analysis demonstrated that the p53 pathway was significantly altered in LFS-BC, with key genes regulating cell cycle arrest, *CDKN1A*, and apoptosis, *BAX*, being down-regulated in LFS-BC and genes involved in suppressing angiogenesis being up-regulated (Figure S9)."

Tumor microenvironment in LFS breast cancers (Page 13, Lines 22-24)

> ***"***Combining these cohorts recapitulated the findings of lower CD8+ and CD20+ cells and higher levels of Ki67 and granzymeB in LFS-BC versus nonLFS-BC tumors (Figure S13a-d)."

11. The authors need to discuss the results on the immunological aspects and microenvironment in light of the recently recognized role of germline alterations in

immunoediting (Houlahan et al. Science 2024, Samayan Immunity) and relate these aspects to their findings (e.g. germline variants could be also a source of immunogenic epitopes). The authors could consider discuss more on the role of CNA burden, aneuploidy and immune response, as they briefly touch upon this aspect when discussing that LFS-BC had lower CD8+ T-cells than non-LFS-BC (Davoli T et al Science 2017). Indeed, a growing body of literature support the inverse link of higher somatic CNA with dampened immune response in BC (Karn T et al. JAMA Oncology 2017; Zerdes I et al. npc Breast Cancer 2021).

We acknowledge the Reviewers' suggestion and agree that the role of germline alterations in immunoediting and the relationship between aneuploidy and immune response is highly relevant to our study. We have added a discussion of this concept along with the suggested references to the Discussion, Page 16, Lines 19-26.

> "Another intriguing finding of our work is that LFS-BC have lower lymphocyte infiltration as compared to non-LFS BC, a finding that is consistent with prior studies showing reduced TILs in LFS-BC[9,14], likely due to higher levels of aneuploidy in LFS-BC[31–33]. In addition, patients with LFS may have a higher germline epitope burden which has recently been suggested to lead to increased immunoediting and immune depleted tumors[34]. Our finding of enrichment of FoxP3+ Tregs and CD68+ macrophages is consistent with a prior CIBERSORT analysis of TCGA data showing that acquired TP53 mutations promoted an immune suppressive environment with infiltration of Tregs, T helper calls and M0-type macrophages[35]."

12. The authors could also add on the aspects of the immune microenvironment on progression from DCIS to invasive cancer in sporadic BC and the immunosuppressive TME (Gil Del Alcazar CR et al. Cancer Discovery 2017 , Strell C. et al. Eur J Cancer 2022)

We agree that this is an important finding, as it suggests that this aspect of BC progression may not be unique to LFS patients. We have added a comment to the Discussion on Page 16 Line 26 to Page 17, Line 1-3.

> "Further, our finding that immune infiltration decreases with progression from DCIS to IDC is consistent with prior work in sporadic BC[36,37], suggesting the necessity of promoting an immune suppressive environment is consistent between LFS and nonLFS patients."

Response to Reviewers
We would like to thank the Editors and Reviewers of *Nature Communications* for their enthusiastic comments and thoughtful review of our manuscript NCOMMS-24-23316R1. We address each of the comments and suggestions in point by-point responses below. Page (P) and Line numbers (L) refer to the Tracked Changes version of the manuscript. We feel that the suggested changes have considerably improved the paper.

Reviewer #1 (Remarks to the Author)

The authors have addressed my specific points well but ignored my general point about reorganising the manuscript. as there are 4 other reviewers and these have not picked up on this point i am prepared to let this ride.

Reviewer #2 (Remarks to the Author)

Remarks to be addressed:

1. *As the quality of the cohort and, thus, the entire study depends on the actual pathogenicity of the detected germline TP53 variants, it is essential for the manuscript to add the information that pathogenicity status of TP53 germline variants was assessed via ClinVar and that variants with conflicting interpretations were included. You could easily add this information in the methods section - Cohort ascertainment (line 447) - "Inclusion criteria for LFS-BC were 1) clinically confirmed TP53 PGV as per ClinVar assessment, including those with conflicting interpretations;...".*
The Reviewer is correct that we included Clinvar likely pathogenic variants with conflicting interpretations. We have added this clarification to the revised manuscript, Page 18, Line 26 and Page 19, Line 1:

> "Inclusion criteria for LFS-BC were 1) clinically confirmed *TP53* PGV as per ClinVar assessment, including those with conflicting interpretations…"

2. lines 126-131: "Tumors with sufficient material for p53 IHC and targeted NGS analysis were available for 21 LFS-BC, 15 surrounding normal and 11 contralateral normal specimens (Figure S1). Among these, eleven cases had DNA binding domain mutations, including known dominant negative loss of function mutations (G245S, R248Q, R110L) and putative hypomorphic mutations (T125M, R181H, P151S, C141Y), seven had loss of function mutations, and four had tetramerization domain mutations (G334R, R337C, R337H) (Figure 1A, Table S1). ----
1) Please specify in this section that all tumor samples were derived from LFS individuals with verified GERMLINE TP53 variant, e.g. "Among these, eleven cases had confirmed germline DNA binding domain mutations...".
2) line 131: "...and four had tetramerization domain mutations (G334R, R337C, R337H)" ---- Please add that two of the latter variants are also putative hypomorphs (in addition to the previously named ones in the DBD).
3) 21 LFS-BC --> 11 + 7 + 4 = 22 variants (unless one individual had two germline variants, this doesn't add up -- if this should be the case, please specify somewhere in the manuscript). Figure 1A suggests that 10 (instead of 11) cases had DBD variants. Please check.
4) Please also detail in this section the number of patients from which the tumors were acquired. Please clarify if by "cases" you refer to tumor samples or to patients.
lines 454-456: "Specimens from twenty-six LFS-BC (46%) from 19 patients were obtained, of which surrounding normal breast tissue (SNBT) and contralateral normal breast tissue (CNBT) were available for analysis in 15 cases and 11 cases respectively (Figure S1, Table S1)."
--- These numbers don't match up with Table S1 and Figure S1. Table S1 relates to 28 LFS-BC

from 22 patients, and only 19 LFS-BC from 16 patients seem to have had sufficient material for IHC and targeted NGS analysis as per columns O and R. Figure S1 relates to 27 LFS-BC from 21 patients. Figure 1A and the results section line 127 refers to 21 LFS-BC.
Figure S1 legend: "The LFS BC cohort contained six ER+Her2- IDC, 12 HER2+ IDC, nine DCIS, 16 (!!) surrounding normal, 11 contralateral normal tissue samples from 21 patients; blood specimens were available from all 22 (!!) patients.The early onset non LFS BC cohort contained 14 ER+Her2 BC, five HER2+ BC and 10 TNBC and 25 surrounding normal tissue samples from 29 (!!) patients with matched normal blood samples."
---- Numbers included in Figure S1 don't match up with numbers in the legend below. Legend still states 16 (not 15) surrounding normal samples. 21 vs. 22 patients with available blood specimens and PGV in LFS group. 27 vs. 29 patients in control group. Also, discrepancies with the main manuscript results and methods section (see above).
Altogether, the numbers urgently need to be clarified and to be consistent throughout the manuscript, which could potentially necessitate reassessment of statistical evaluation.
It needs to become clear to the reader 1) how many LFS-BC from 2) how many individuals with 3) how many different TP53 variants were part of the final analysis. Please also indicate in Table S1 which of the LFS-BC samples were included in the final analysis and which were left out due to insufficient material.

We appreciate the Reviewer's attention to detail regarding sample numbers and mutation status. We have added a table describing the number of tissues and number of patients that were utilized in at least one assay to **Supplementary Figure 1**. The specific samples that underwent each assay can be found in Columns O-S of **Supplementary Table 1**. In addition, we have created a separate section in the manuscript entitled "Multi-omic analysis of LFS breast cancer" to describe the entire cohort number of tissues and patients that underwent assays on Page 5 Line 26 and Page 6 Lines 1-11:

> "Multi-omic analysis of LFS breast cancer
> Breast tumors including both invasive ductal carcinoma (IDC) and ductal carcinoma in situ (DCIS), surrounding normal breast tissue, contralateral normal breast tissue, and blood specimens were obtained for 28 breast cancers from 22 patients with LFS and 29 breast cancers from 28 patients from early-onset non-LFS patients and subjected to multi-omic studies (Figure S1, Table S1). Ten patients with 14 tissues had germline DNA binding domain mutations, including known dominant negative loss of function mutations (G245S, R248Q, R110L) and putative hypomorphic mutations (T125M, R181H, P151S, P151T, C141Y), eight patients with nine tissues had germline loss of function mutations (large deletions, c.993+1G>A, p.C123X, p.T125T, p.R196X), and four patients with five tissues had germline tetramerization domain mutations (putative hypomorphic mutations, G334R, R337H and loss of function mutation, R337C) (Figure S1, TableS1)."

In addition, we corrected the numbers in the Methods on Page 19, Lines 9-11.

Reviewer #4 (Remarks to the Author)

We jointly agree that the authors have replied satisfactory to the raised questions, both by us and the other reviewers.

Reviewer #5 (Remarks to the Author)

The authors have successfully addressed my comments and provided additional information that overall improved the manuscript, results' presentation and readability.
